# Genomic Analyses of Breast Cancer Progression Reveal Distinct Routes of Metastasis Emergence

**DOI:** 10.1038/srep43813

**Published:** 2017-03-09

**Authors:** Anne Bruun Krøigård, Martin Jakob Larsen, Charlotte Brasch-Andersen, Anne-Vibeke Lænkholm, Ann S. Knoop, Jeanette Dupont Jensen, Martin Bak, Jan Mollenhauer, Mads Thomassen, Torben A. Kruse

**Affiliations:** 1 Department of Clinical Genetics, Odense University Hospital, Odense, Denmark; 2 Human Genetics, Institute of Clinical Research, University of Southern Denmark, Odense, Denmark; 3 Department of Pathology, Slagelse Hospital, Slagelse, Denmark; 4 Department of Oncology, Rigshospitalet, Copenhagen, Denmark; 5 Department of Oncology, Odense University Hospital, Odense, Denmark; 6 Department of Pathology, Odense University Hospital, Odense, Denmark; 7 Lundbeckfonden Center of Excellence NanoCAN, Odense, Denmark; 8 Molecular Oncology Group, Institute of Molecular Medicine, University of Southern Denmark, Odense, Denmark

## Abstract

A main controversy in cancer research is whether metastatic abilities are present in the most advanced clone of the primary tumor or result from independently acquired aberrations in early disseminated cancer cells as suggested by the linear and the parallel progression models, respectively. The genetic concordance between different steps of malignant progression is mostly unexplored as very few studies have included cancer samples separated by both space and time. We applied whole exome sequencing and targeted deep sequencing to 26 successive samples from six patients with metastatic estrogen receptor (ER)-positive breast cancer. Our data provide support for both linear and parallel progression towards metastasis. We report for the first time evidence of metastasis-to-metastasis seeding in breast cancer. Our results point to three distinct routes of metastasis emergence. This may have profound clinical implications and provides substantial novel molecular insights into the timing and mutational evolution of breast cancer metastasis.

Next generation sequencing (NGS) has been extensively applied to catalogue the mutational landscapes of large numbers of primary breast tumors^1^,^2^,^3^,^4^,^5^ but the mutational evolution through breast cancer progression and the timing of metastasis have remained elusive. In pancreatic^6^ and colorectal cancer^7^, distant metastasis has been reported a late event in molecular time. Two studies of single breast cancer patients have provided support for a linear progression model, where metastases emerge from late occurring advanced clonal subpopulations^8^,^9^. Opposed to this, the parallel progression model suggests early metastasis seeding and independent acquisition of mutations in the primary tumor and the disseminated cells^10^. The parallel progression model implies that cancer is a systemic disease at an early time point and for this progression pattern systemic adjuvant treatment is necessary^11^. In general, close genetic ties between primary tumor and metastasis have been considered indicative of linear progression, whereas genetic divergence is interpreted as evidence of parallel progression^12^.

In agreement with Naxerova *et al*.^12^, we consider linear progression as metastasis seeding from the most advanced clone of the primary tumor. By contrast, parallel progression, is considered to have taken place if more advanced clones within the primary tumor can be found in addition to the clone giving rise to the metastasis.

A third mechanism, the concept of metastasis-to-metastasis seeding, the cascade hypothesis^13^, was proposed in 1975. Until recently, this phenomenon has not been proven by high resolution studies. In prostate cancer, very recent NGS studies including four^14^ and ten^15^ primary tumor-metastasis pairs identified a subset of tumors with evidence for metastasis-to-metastasis seeding.

In this study, we provide evidence of three distinct routes of breast cancer progression and find diverse patterns of progression among and even within patients.

## Results

### Large variation in mutational concordance between the different steps of metastatic progression

We analyzed a set of six breast cancers with matched premalignant, synchronous axillary lymph node metastases (sALN) and/or asynchronous distant metastases (aDM) (Table 1, Supplementary Table S1). aDM from four of the patients were biopsied with a relapse time between 1.82–4.05 years. Paired-end exome sequencing (Supplementary Table S2) was applied to the 26 samples, followed by identification of potential somatic mutations by using the union of results from nine different somatic variant callers. Targeted deep sequencing (200–613×coverage) (Supplementary Table S3) was used to increase data quality followed by stringent filtering of somatic mutations in the coding region (44–531 somatic mutations per patient). A full table of somatic mutations and their allele frequency is included (Supplementary Tables S4–S9).

Large variation in mutational concordance was observed between the different steps of malignant progression of the studied patients (Supplementary Fig. S1). Furthermore, deep sequencing enabled us to detect small subclonal populations within each tumor sample, visualized by frequency plots.

Bubble Tree analysis^16^ was performed to estimate tumor purity, ploidy and clonality, inferred from exome sequencing data (Supplementary Figs S2–S21). The tumor purity of the samples, based on these analyses, ranges between 0.24–0.95 (Supplementary Table S2).

### Linear progression towards metastasis

**Figure 1:**
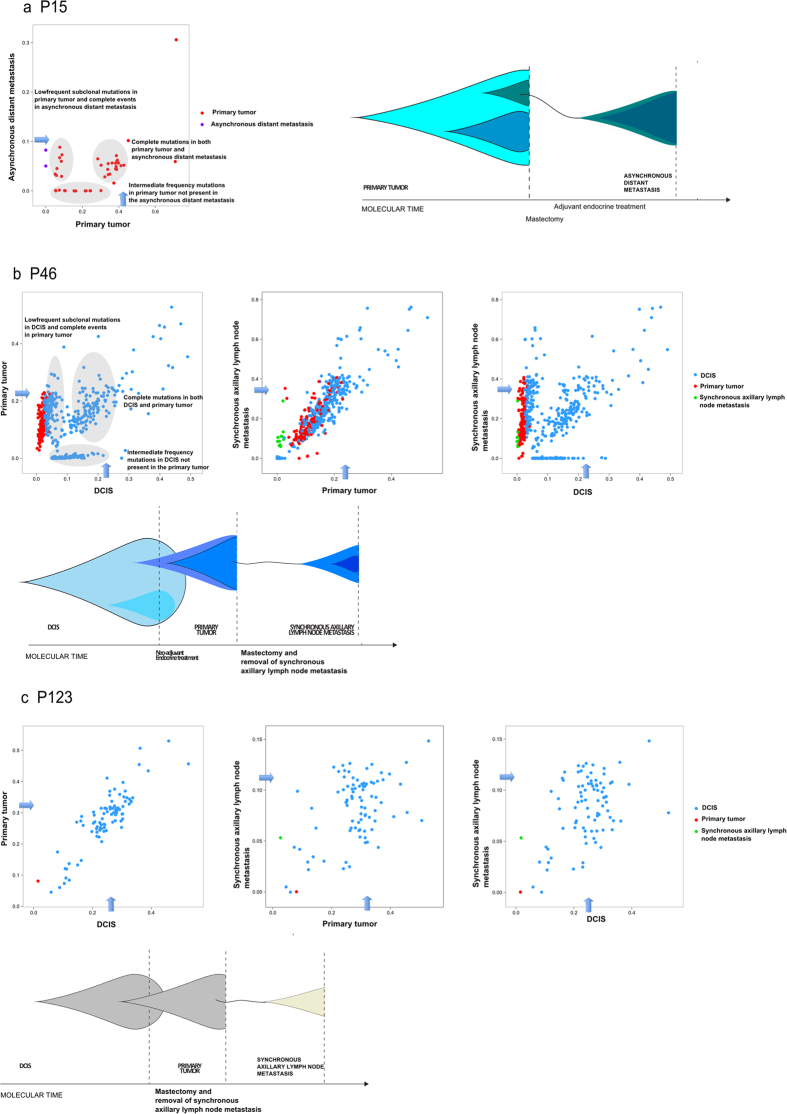
Linear progression of metastases. Allele frequency plots and phylogenetic trees for (**a**) P15, (**b**) P46 and (**c**) P123. Allele frequency plots, based on targeted deep sequencing data, comparing frequencies of validated somatic mutations in the coding region between different steps of malignant progression. Each dot represents a somatic non-synonymous, splicing or synonymous somatic mutation. The color code indicates the step at which the mutation appears for the first time. Dots in blue, red, green and purple depict mutations appearing for the first time in pre-invasive tissue, primary tumor, synchronous axillary lymph node metastases (sALN) and asynchronous distant metastasis (aDM), respectively. The arrows indicate the level corresponding to the diploid, heterozygous allele frequency of the sample, derived from Bubble Tree analysis. Phylogenetic trees depict the clonal evolution through cancer progression. An increase in color intensity reflects the acquisition of additional somatic mutations.

Patients P15, P46 and P123 displayed 42, 430 and 86 mutations in the primary tumors, of which 29 (69%), 426 (99%) and 80 (93%), respectively were shared with the matching metastases (Supplementary Fig. S1). All three cases showed relatively high genetic concordance between successive steps of cancer progression and thus, the cancer genomes of these patients presented fairly stable. Phylogenetic analyses, depicted by mutational frequency plots, provided straight forward evidence that metastases in these three cases originated from the most recent and advanced subclone of the primary tumor in accordance with the linear progression model (Fig. 1a–c). This is supported by the purification of subclonal mutations i.e. increased mutation frequencies in the metastases of P 15 and 46, while no subclones appeared in P123.

Interestingly, the pre-invasive sample of P46 has a number of mutations that are not found in the primary tumor. These subclonally occurring mutations in DCIS are most likely present in a different subclone than the one giving rise to the invasive lesion, depicted as the turquoise subclone within the DCIS in Fig. 1b.

### Co-occurrence of linear and parallel progression towards metastasis

**Figure 2:**
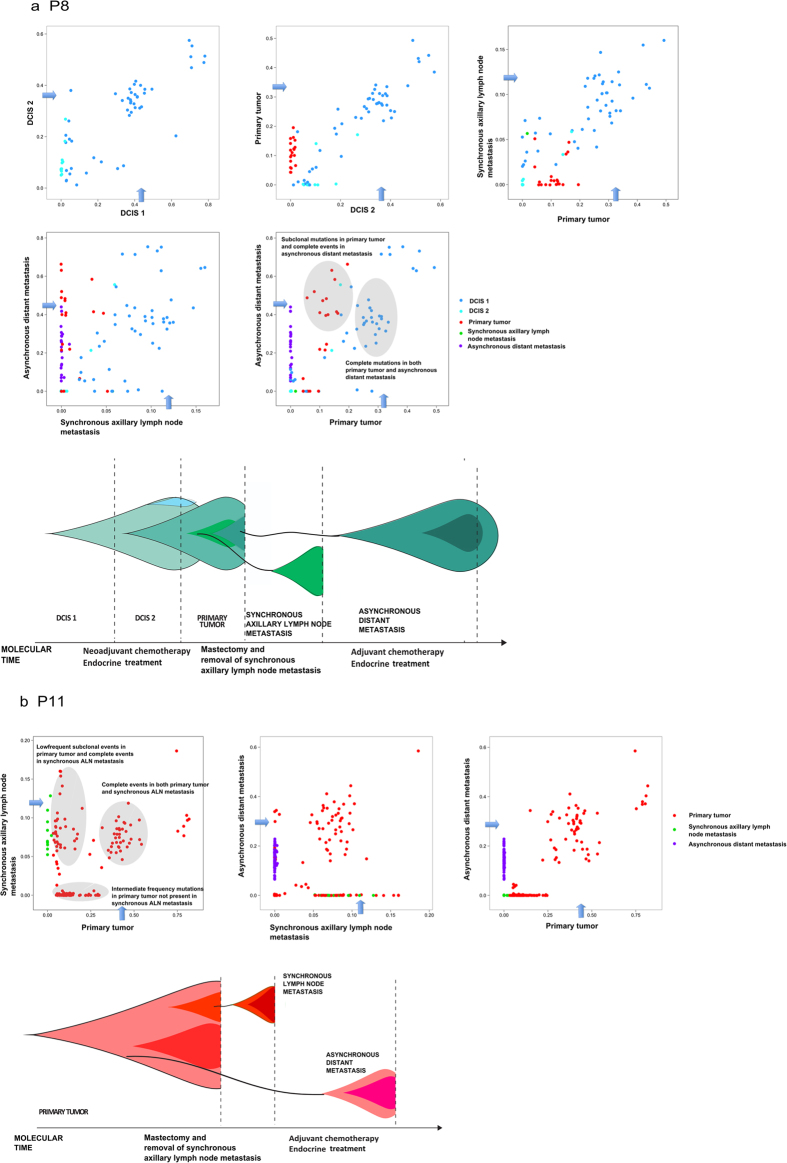
Diverse patterns of progression within patients. Allele frequency plots and phylogenetic trees for (**a**) P8 (**b**) P11. Allele frequency plots, based on targeted deep sequencing data, comparing frequencies of validated somatic mutations in the coding region between different steps of malignant progression. Each dot represents a somatic non-synonymous, splicing or synonymous somatic mutation. The color code indicates the step at which the mutation appears for the first time. Dots in blue/turquoise, red, green and purple depict mutations appearing for the first time in pre-invasive tissue, primary tumor, sALN and aDM, respectively. The arrows indicate the level corresponding to the diploid, heterozygous allele frequency of the sample, derived from Bubble Tree analysis. Phylogenetic trees depict the clonal evolution through cancer progression. An increase in color intensity reflects the acquisition additional somatic mutations.

Patient P8 and P11 displayed diverse models of metastatic spread (Fig. 2a,b). In both patients, one metastasis arose in accordance with the linear progression model and the other in accordance with the parallel progression model. In P8, the aDM emerged linearly, i.e. from the most recent subclone within the primary tumor. By contrast, the sALN disseminated in accordance with the parallel progression model, as the metastasis originated from an earlier stage of primary tumor evolution as a number of mutations subclonally occurring in the primary tumor are not present within the sALN. Subclonal mutations in the primary tumor constituted complete clonal events in the metastases. This purifying effect, revealed by mutation frequencies, confirms the subclonal origin of the metastases. In P11 the sALN emerged linearly, while the aDM developed in accordance with the parallel progression model. The aDM contained only a few of the subclonally occurring mutations in the primary tumor. It harbored predominantly the complete, early mutations as well as 77 new mutations, which are exclusive to this metastasis, revealing parallel progression of this metastasis (Fig. 2b). Thus, the two metastases originated from two different cell populations within the primary tumor, supported by the fact that no mutations are shared solely by the two different metastases. In both P8 and P11 a substantial amount of additional mutations were found in the metastases. Parallel progression of metastasis, indicated by high mutational discordance, bears profound clinical implications as clonal divergence between primary tumors and metastases opens potentially broad windows for therapy escape.

### Metastasis-to-metastasis seeding

**Figure 3:**
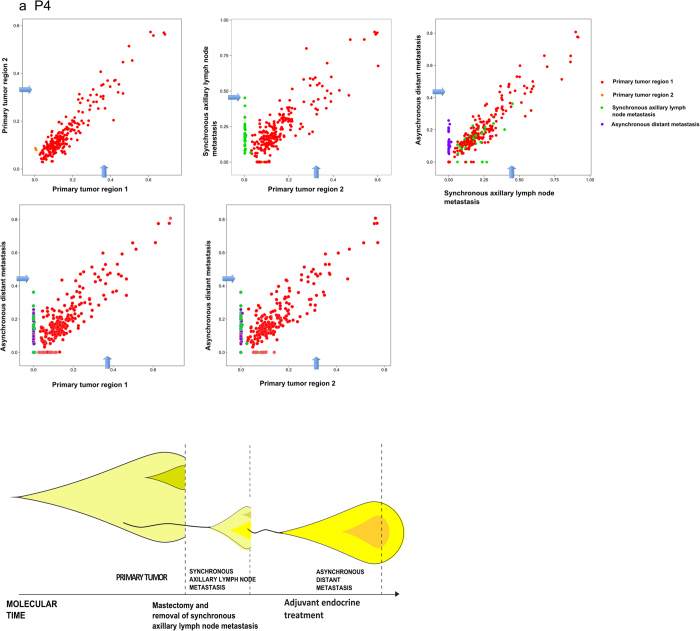
Metastasis to metastasis seeding. Allele frequency plot and phylogenetic tree for (**a**) P4. The allele frequency plots, based on targeted deep sequencing data, compare frequencies of validated somatic mutations in the coding region between different steps of malignant progression. Each dot represents a somatic non-synonymous, splicing or synonymous somatic mutation. The color code indicates the step at which the mutation appears for the first time. Dots in red/orange, green and purple depict mutations appearing for the first time in primary tumor, sALN and aDM, respectively. The arrows indicate the level corresponding to the diploid, heterozygous allele frequency of the sample, derived from Bubble Tree analysis. The phylogenetic tree depicts the clonal evolution through cancer progression. An increase in color intensity reflects the acquisition additional somatic mutations.

Most remarkably, patient P4 displayed somatic mutational patterns revealing seeding of the aDM from the sALN as 36 mutations, not detectable in the primary tumor, were shared by the two metastases (Fig. 3). Further, 26 additional mutations are found in the aDM, revealing high mutational discordance between primary tumor and metastatic lesions in this case. The founder cell of the aDM disseminated from the sALN, supported by the fact that all mutations shared between the aDM and the primary tumor are also found in the sALN. To our knowledge, this is the first time that metastasis-to-metastasis seeding is reported for breast cancer.

Rather surprisingly, some of the mutations originating from the primary tumor in P4 appeared with a similar frequency pattern in primary tumor and metastases. Such pattern could emerge as a result of complex copy number aberrations. To explore this hypothesis we conducted micro array analysis on the data (Supplementary Figs S22 and S23). Indeed, this revealed very few regions to be diploid. Hence, this cancer cell population has a more complex pattern of somatic copy number mutations and hyperploidy with varying number of point mutations within the different alleles explaining the retained mutation frequencies in the metastases. Another explanation to the phenomenon could be that not a single cell but a cluster of non-identical primary tumor cells founded the metastatic lesion as recently suggested by others^17^,^18^.

### Mutations found exclusively in metastases

We finally analyzed the mutant gene sets derived from the studies. Mutations found exclusively in the metastases (Supplementary Table S10) affected the *CREBBP* and *PPP2R1A* genes which are already included as cancer-related genes in the Catalogue Of Somatic Mutations In Cancer (COSMIC) Cancer Gene Census list (http://cancer.sanger.ac.uk/cancergenome/projects/census/). The *CREBBP* gene is an epigenetic modifier acting as a transcriptional coactivator through acetylation of histone proteins and has been suggested as tumor suppressor^19^. Further, *BCL6B* and *ZNF185* were among the genes with exclusive mutations in the metastases. The *BCL6B* gene has recently been reported as novel tumor and metastasis suppressor in hepatocellular carcinoma^20^. Stable expression of the *BCL6B* gene in hepatocellular cell lines is found to suppress cell migration and invasion and significantly reduce the incidence and severity of lung metastases in a mouse model^20^. The *ZNF185* gene has been suggested to function as a tumor suppressor and has been associated with metastatic progression in colon and prostate cancer^21^,^22^.

## Discussion

We are to the best of our knowledge, the first to report a detailed description of the mutational evolution through successive steps of breast cancer progression with establishment of the clonal origin of metastases, revealing complex patterns of metastatic spread. Linear progression from the primary tumor is a common event observed in 5 out of 6 cases. However, two of these cases showed evidence for the co-occurrence of parallel progression from early seeding events. It cannot be ruled out that such co-occurrence is more frequent than suggested by the presented cases, because for each of the three cases in which exclusively linear progression was observed only a single metastasis was analyzed. Further, it cannot be ruled out that additional low-frequency subclones, below our detection limit, may have escaped our attention, especially in the few samples with a tumor content around 25%. However, in these samples the malignant cells have been sequenced at a depth of around 20× and somatic variants have been called by a non-conservative, sensitive method followed by deep sequencing. This allow us to detect medium frequency subclones. We provide evidence of parallel progression of two sALN metastases (P4 and P8) and two aDM (P4 and P11) and these findings are in contrast to the two previous reports of single matched pairs, which only uncovered support for linear progression in breast cancer metastasis emergence^8^,^9^.

Metastasis-to-metastasis seeding is the best fitting evolutionary model considering the available data, however, alternative evolution routes cannot be definitively excluded. As the complete mass of the primary tumor was not sequenced it is possible that the two metastases originated from an undetected subclone within the primary tumor.

The main difference between the linear and parallel progression model is the *timing* of dissemination from the primary tumor as continuous genome evolution within a cancer cell population entail that the primary tumor will evolve further after early dissemination of a parallel progression metastasis founder cell. Thus, from a mutational point of view, the question is whether a metastasis is seeded from the most advanced clone of the primary tumor. However, it is important to stress that different selection processes act on primary tumor and metastatic cells, as tumorigenic and metastatic features are not identical. Rather, selective forces are timing and site dependent. Metastasis can be viewed as an evolutionary process in itself and different genes are believed to be involved in different steps of the metastatic process^23^. Thus, the most advanced clone of the primary tumor is not necessarily the one to give rise to metastases, which is in agreement with our results.

Early acquired mutations, present in all malignant cells in copy number neutral regions are expected to present a mutation allele frequency around tumor purity x 0.5. This pattern is in fact present in the frequency plots, based on point mutation data inferred from deep sequencing data. Alternative estimates, based on Bubble Tree analysis, inferred from exome sequencing data, and visualized by the blue arrows in Figs 1, 2, 3 in most cases support this. The Bubble Tree analysis becomes inaccurate at very low tumor purity levels and therefore overestimate the diploid heterozygous level in a few cases.

The phylogenetic trees of clonal evolution presented here are inferred from the mutational frequency based on deep sequencing data and our conclusions are based on copy number neutral heterozygous positions. Notably, the purifying effect seen from one sample to the next, i.e. subclonally occurring mutations in one sample that are seen to be purified to constitute complete events in the consecutive tumor step are informative of the subclonal origin of metastases.

For P8, P46 and P123 the availability of the ductal carcinoma *in situ* (DCIS) allowed us to trace back the evolution of the primary tumor and consecutive metastases to a subclone already emerging at the stage of this precursor lesion. Clonal heterogeneity was found in most tumor samples. The fact that the mutations found exclusively in the aDM of P4, P8, P11 and the sALN of P46 occur subclonally reveal that mutations of the metastasis founder cell from the primary tumor were sufficient to establish the metastatic lesion, but that metastasis-specific mutations may confer additional clonal advantages, e.g. hypoxia resistance or angiogenesis.

To our knowledge, we present the first evidence of seeding of a distant metastasis from a lymph node metastasis in breast cancer, in accordance with the very recent observations in prostate cancer. Thus, breast cancer metastases may emerge via three different routes: linear progression with late dissemination from the primary tumor, early dissemination of the metastasis founder cell leading to parallel progression of primary tumor and metastasis and importantly, metastasis-to-metastasis seeding. The genetic disparity between different metastases within the same patient and metastasis-to-metastasis seeding in breast cancer has profound clinical implications with regard to therapeutic strategies and emphasizes the potential relevance of circulating tumor cell and circulating tumor DNA-based surveillance. Our study reveals diverse models of metastasis among patients and even within individual patients and emphasize that we have yet to arrive at a complete understanding of the metastatic process.

## Materials and Methods

### Patient material

The study includes successive tumor samples from six breast cancer patients with estrogen receptor (ER) positive invasive ductal carcinoma. Supplementary Table S1 displays clinical information of the patients. All patients had synchronous axillary lymph node (sALN) metastases at the time of diagnosis and material from primary tumors and sALN metastases from five of the patients were secured during primary surgery and stored at −80 °C until sample preparation. In three cases, also pre-invasive stages, Ductal Carcinoma in Situ (DCIS) were secured during primary surgery. In one case, we had access to two different regions of DCIS (P8) and in one case, two different regions of primary tumor (P4). In spite of adjuvant therapy, four of the patients experienced recurrence of the disease, with a median relapse time of 3.08 years, and asynchronous metastases were biopsied from bone, lymph node and in two cases liver, respectively. Haematoxylin-eosin sections of all tissue samples were reviewed by a certified pathologist, ensuring the diagnosis. A start amount of 20–30 mg fresh frozen tissue (asynchronous metastasis 5 mg) was used for the purification process. Tissue disruption and homogenization was performed using TissueLyser (Qiagen) and purification of DNA was performed using AllPrep DNA/RNA Mini Kit (Qiagen). Matched normal tissue and the primary tumor of PT ID 8 were stored as formalin-fixed paraffin-embedded (FFPE) tissue. The FFPE blocks were cut in 30–40 sections of 10 μm and DNA extracted using AS1000 Maxwell 16 (Promega, USA).

Parts of the analysis of P8 have previously been described in ref. 9.

### Ethics statement

Informed consent was obtained from all patients. All experimental protocols were approved by the Ethical Committee of Region Syddanmark and notified to the Danish Data Protection Agency. The methods were carried out in accordance with the approved guidelines.

### Library construction and exome sequencing

One microgram of genomic DNA from each sample was randomly fragmented by focused acoustic shearing (Covaris inc.) according to Illumina’s protocol. The fragment length was measured by Bioanalyzer (Agilent Technologies 2100), confirming a fragment length of 150–300 bp. Exome enrichment was performed with Illumina’s TruSeq DNA Sample Preparation. Paired end sequencing of 2 × 100 bases was performed on the Illumina HiSeq 1500 platform. FASTQ files were aligned to the human reference genome GRCh37 (feb. 2009) using the Novoalign v. 3 algorithm (www.novocraft.com) at default parameters. Removal of duplicate reads, recalibration and local realignment around indels were performed using Best Practices pipeline v. 2.7^24^. The result was a mean coverage rate in the exome region of 11–155×(Supplementary Table S2).

### Detection of putative somatic mutations

#### Detection of putative somatic mutations for deep sequencing

On the exome sequencing data, somatic variant calling was performed using nine publicly available somatic variant callers: EB Call^25^, Mutect^26^. Seurat^27^, Shimmer^28^, Indelocator (http://www.broadinstitute.org/cancer/cga/indelocator), Somatic Sniper^29^. Strelka^30^, Varscan 2^31^ and Virmid^32^. All putative somatic mutations reported by at least one somatic variant caller, except positions in intronic, intergenic, downstream and non-coding RNA intronic areas, were used to select chromosomal candidate regions for targeted deep sequencing.

#### Targeted deep sequencing of candidate regions

Target enrichment was performed using SureSelect DNA enrichment methodology (Agilent). A custom SureSelect enrichment kit was designed using the Agilent SureDesign application. Library construction and SureSelect enrichment were performed according to manufacturer’s protocol and sequenced on the Illumina HiSeq 1500 platform with paired end sequencing 2 × 100 bases. Deep sequencing resulted in a mean coverage of 200–613× of the targeted positions (Supplementary Table S3). Alignment and data preprocessing were performed as described above. Variant calling were performed using Varscan 2^31^ version 2.3.6 (multisample setting). For each patient the following criteria were used: normal sample B Allele Frequency (BAF) less than 0.02, all samples should have a read depth of min. 50× and BAF in one of the tumor samples should be 0.05 at minimum. For positions meeting those criteria, a mutation found with a BAF of 0.025 at minimum was included in other tumor samples with if read depth exceeded 200×. The variants were annotated with Annovar and only exonic and splicing variants were included for further analysis. Known SNPs with a population allele frequency >1% were excluded.

Subsequently, all identified somatic mutations in the coding region were manually curated, by visual inspection of the BAM files to remove false positive calls. Variants located in a repetitive area and variants located in SNV clusters were excluded, as they most likely result from systematic misalignment. Furthermore, unrelated BAM files were compared to the patient BAM files in order to identify error prone regions.

#### Data presentation

Variant allele frequency plots based on targeted deep sequencing data were created revealing the timing and distribution of mutations occurring during progression. Each tumor sample was compared to previous steps of progression. In spite of varying degrees of normal cell admixture, the frequency plots allow distinction between different clusters of mutations. The phylogenetic trees of the clonal evolution models represent the most likely explanation. A threshold of at least two mutations was set to define a new subclone.

#### Estimation of tumor purity and establishment of the 2n heterozygous allele level

To estimate tumor cell content, tumor ploidy, allele-specific copy number and clonality we utilized the bioinformatics framework Bubble Tree^16^ which presents the data as intuitive graphs (Supplementary Figs S2–S21). The analysis is based on the exome sequencing data and takes both copy number variation and germline heterozygous SNP data into account.

Based on the resulting purity estimates, the level corresponding to the diploid, heterozygous allele frequency was added to the variant allele frequency plots (Figs 1, 2, 3), indicated by an arrow, which makes the BAF measurements between the samples more comparable.

#### Microarray

DNA from tumor samples of P4 were analyzed using Affymetrix Cytoscan HD array (Affymetrix, inc. Santa Clara, CA, USA), (Supplementary Figs S22 and S23). Data analysis on the array data was performed using the Nexus 7.5 software (Biodiscovery).

## Additional Information

**How to cite this article:** Krøigård, A. B. *et al*. Genomic Analyses of Breast Cancer Progression Reveal Distinct Routes of Metastasis Emergence. *Sci. Rep.* **7**, 43813; doi: 10.1038/srep43813 (2017).

**Publisher's note:** Springer Nature remains neutral with regard to jurisdictional claims in published maps and institutional affiliations.

## Supplementary Material

Supplementary Material

## Figures and Tables

**Table 1:** Samples included in the study.

P	Number of tumor samples				Time to relapse
	DCIS	Primary tumor	sALN	aDM	
4		2	1	1 (liver)	1.82 years
8	2	1	1	1 (contr. LN)	4.05 years
11		1	1	1 (liver)	2.57 years
15		1		1 (bone)	3.90 years
46	1	1	1		
123	1	1	1		

P: patient. DCIS: ductal carcinoma *in situ*. sALN: synchronous axillary lymph node metastasis. aDM: asynchronous distant metastasis. Contr. LN: contralateral lymph node metastasis.
