# Supplementary material for: Genomic Analyses of Breast Cancer Progression Reveal Distinct Routes of Metastasis Emergence
Source: Sci Rep. 2017 Mar 9;7:43813. doi: 10.1038/srep43813 (PMC5343450; doi:10.1038/srep43813)
Supplement: Supplementary Material [file srep43813-s1.pdf]

Supplementary Material

Genomic Analyses of Breast Cancer Progression Reveal Distinct Routes of Metastasis Emergence

Authors

Anne Bruun Krøigård[1,2], Martin Jakob Larsen[1,2], Charlotte Brasch-Andersen[1,2], Anne-Vibeke Lænkholm[3], Ann S. Knoop[4], Jeanette Dupont Jensen[5], Martin Bak[6], Jan Mollenhauer[7,8], Mads Thomassen[1,2,7], Torben A. Kruse[1,2,7]

Affiliations

1. Department of Clinical Genetics, Odense University Hospital, Odense, Denmark.
2. Human Genetics, Institute of Clinical Research, University of Southern Denmark, Odense, Denmark.
3. Department of Pathology, Slagelse Hospital, Slagelse, Denmark.
4. Department of Oncology, Rigshospitalet, Copenhagen, Denmark.
5. Department of Oncology, Odense University Hospital, Odense, Denmark.
6. Department of Pathology, Odense University Hospital, Odense, Denmark.
7. Lundbeckfonden Center of Excellence NanoCAN.

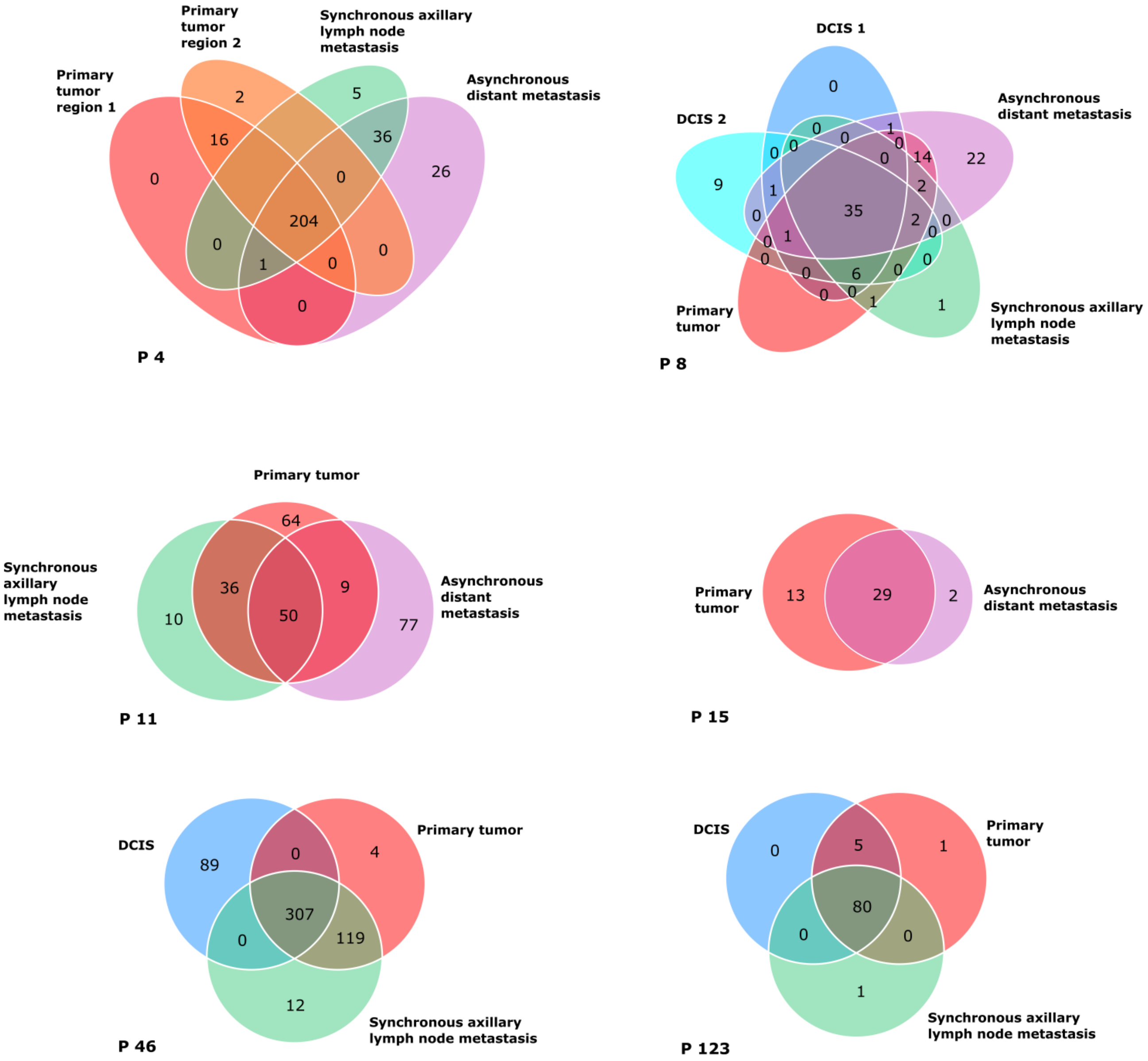

Supplementary Figure S1. Venn diagrams of the mutational concordance of non-synonymous, splicing and synonymous somatic mutations between the different steps of malignant progression of the six studied patients.

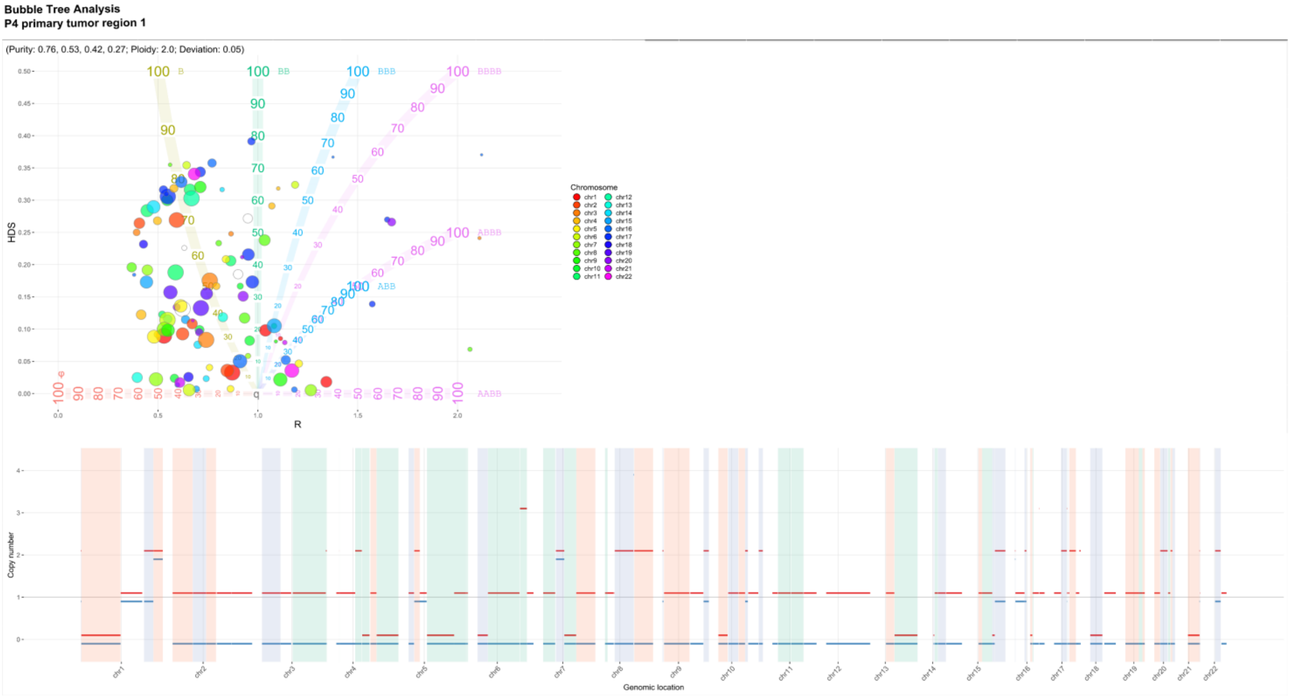

Supplementary Figure S2. BubbleTree analysis graph and copy number plot of P4 primary tumor region 1. The BubbleTree analysis is based on exome sequencing data. In the upper panel, the *R*-HDS plot, the *R* score indicates the copy number ratio between the tumor and matched normal sample, and HDS is the heterozygous-deviation score. The tree branches represent the integer allele-specific copy numbers. The lower panel depicts the copy number events. The tumor cell content of each sample is estimated, indicated by the first figure in the purity estimate. The following figures represent the frequency of major subclones.

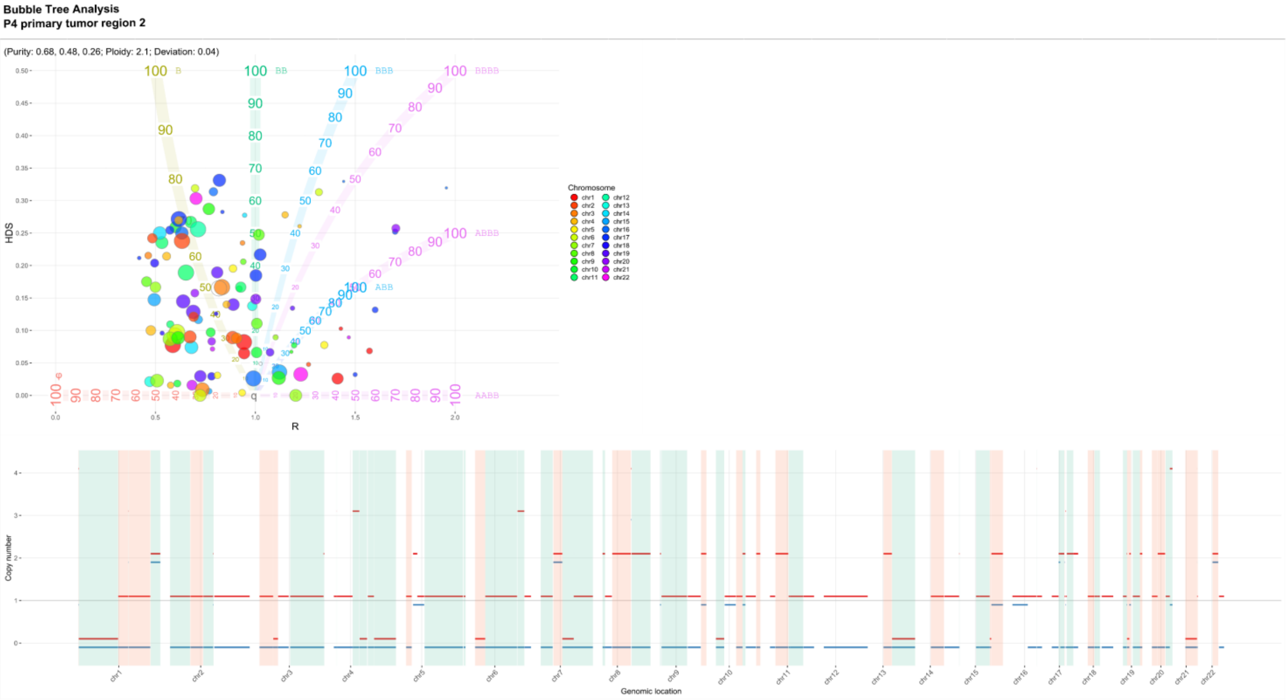

Supplementary Figure S3. BubbleTree analysis graph and copy number plot of P4 primary tumor region 2. The BubbleTree analysis is based on exome sequencing data. In the upper panel, the *R*-HDS plot, the *R* score indicates the copy number ratio between the tumor and matched normal sample, and HDS is the heterozygous-deviation score. The tree branches represent the integer allele-specific copy numbers. The lower panel depicts the copy number events. The tumor cell content of each sample is estimated, indicated by the first figure in the purity estimate. The following figures represent the frequency of major subclones.

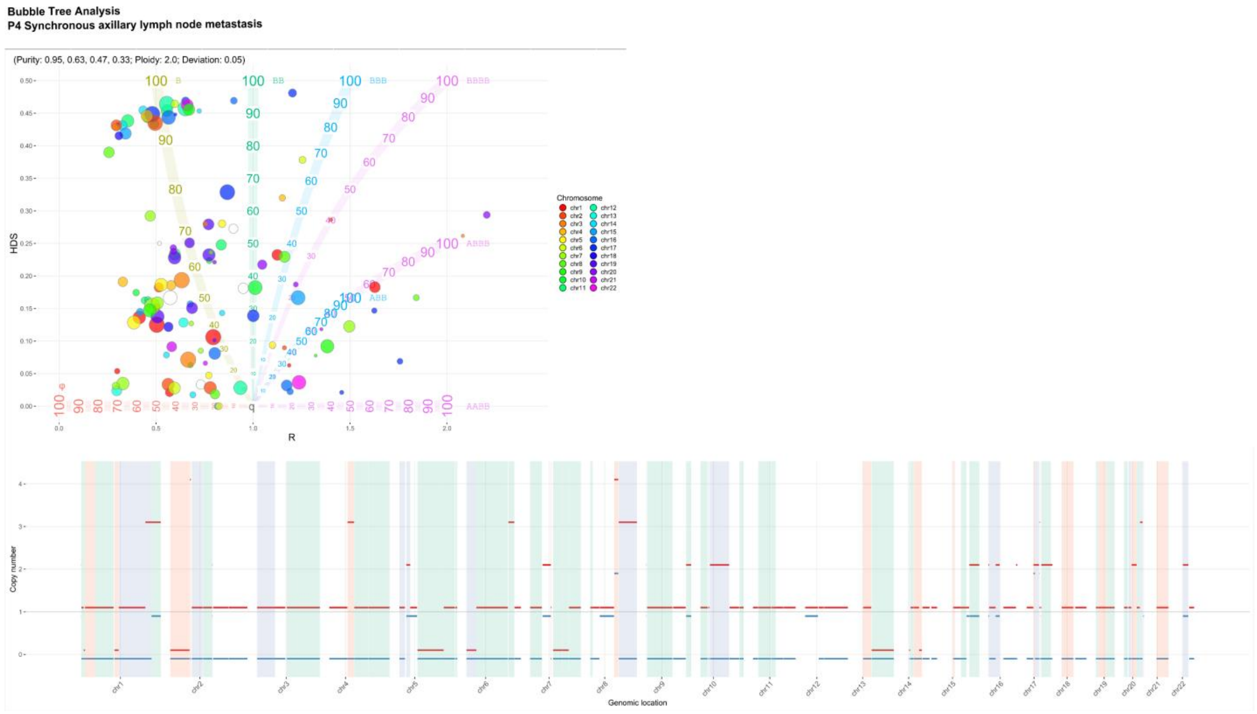

Supplementary Figure S4. BubbleTree analysis graph and copy number plot of P4 synchronous axillary lymph node metastasis. The BubbleTree analysis is based on exome sequencing data. In the upper panel, the *R*-HDS plot, the *R* score indicates the copy number ratio between the tumor and matched normal sample, and HDS is the heterozygous-deviation score. The tree branches represent the integer allele-specific copy numbers. The lower panel depicts the copy number events. The tumor cell content of each sample is estimated, indicated by the first figure in the purity estimate. The following figures represent the frequency of major subclones.

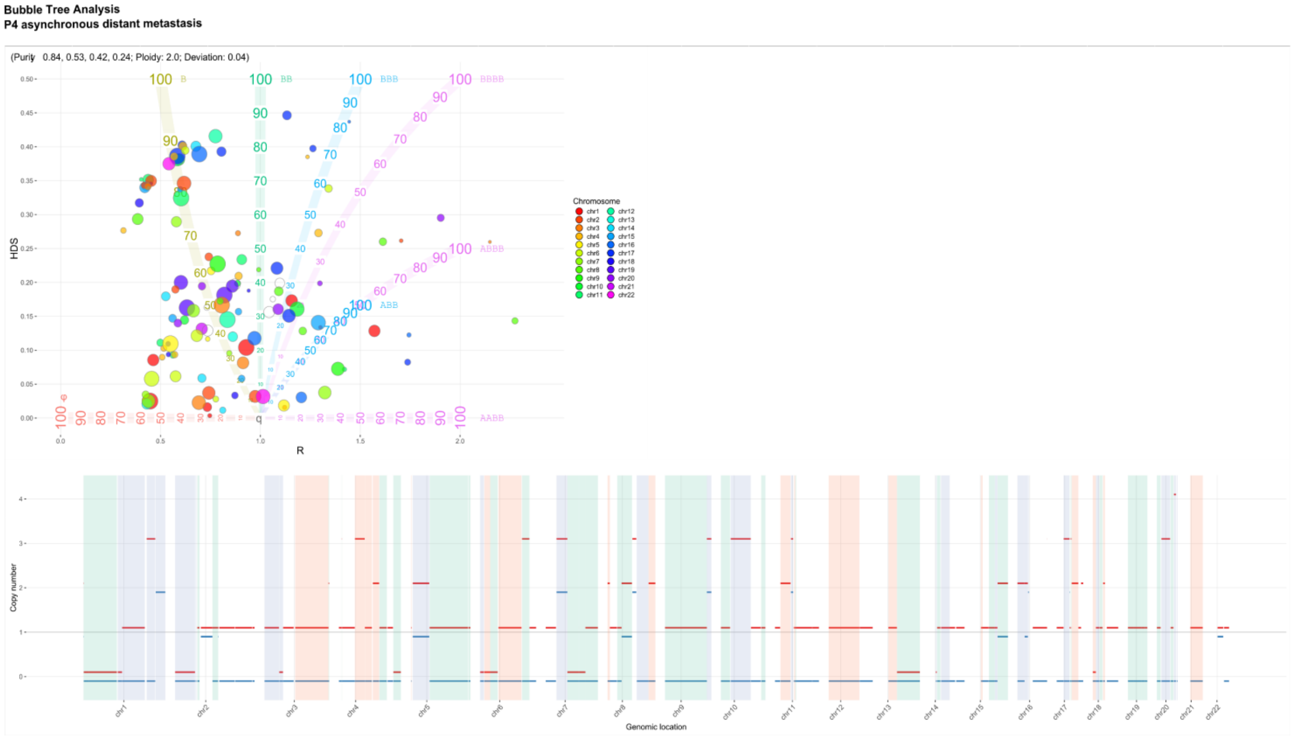

Supplementary Figure S5. BubbleTree analysis graph and copy number plot of P4 asynchronous distant metastasis. The BubbleTree analysis is based on exome sequencing data. In the upper panel, the *R*-HDS plot, the *R* score indicates the copy number ratio between the tumor and matched normal sample, and HDS is the heterozygous-deviation score. The tree branches represent the integer allele-specific copy numbers. The lower panel depicts the copy number events. The tumor cell content of each sample is estimated, indicated by the first figure in the purity estimate. The following figures represent the frequency of major subclones.

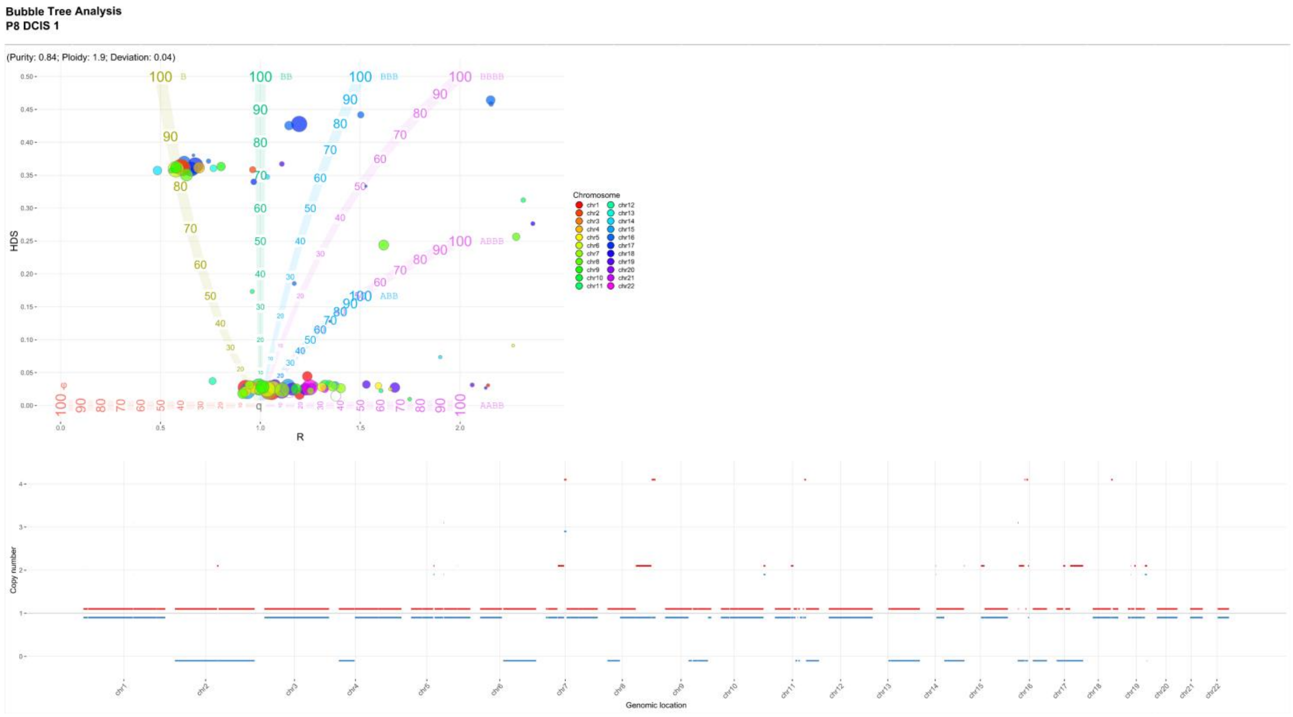

Supplementary Figure S6. BubbleTree analysis graph and copy number plot of P8 DCIS 1. The BubbleTree analysis is based on exome sequencing data. In the upper panel, the *R*-HDS plot, the *R* score indicates the copy number ratio between the tumor and matched normal sample, and HDS is the heterozygous-deviation score. The tree branches represent the integer allele-specific copy numbers. The lower panel depicts the copy number events. The tumor cell content of each sample is estimated, indicated by the first figure in the purity estimate. The following figures represent the frequency of major subclones.

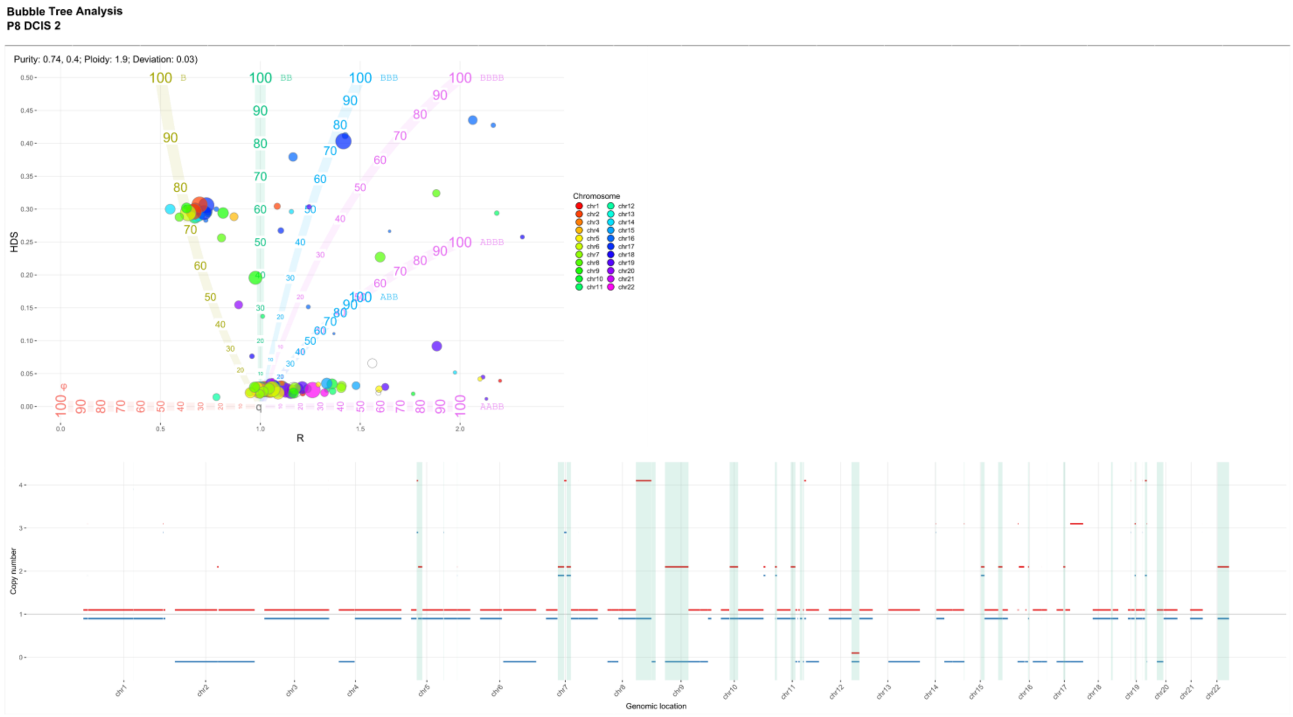

Supplementary Figure S7. BubbleTree analysis graph and copy number plot of P8 DCIS 2. The BubbleTree analysis is based on exome sequencing data. In the upper panel, the *R*-HDS plot, the *R* score indicates the copy number ratio between the tumor and matched normal sample, and HDS is the heterozygous-deviation score. The tree branches represent the integer allele-specific copy numbers. The lower panel depicts the copy number events. The tumor cell content of each sample is estimated, indicated by the first figure in the purity estimate. The following figures represent the frequency of major subclones.

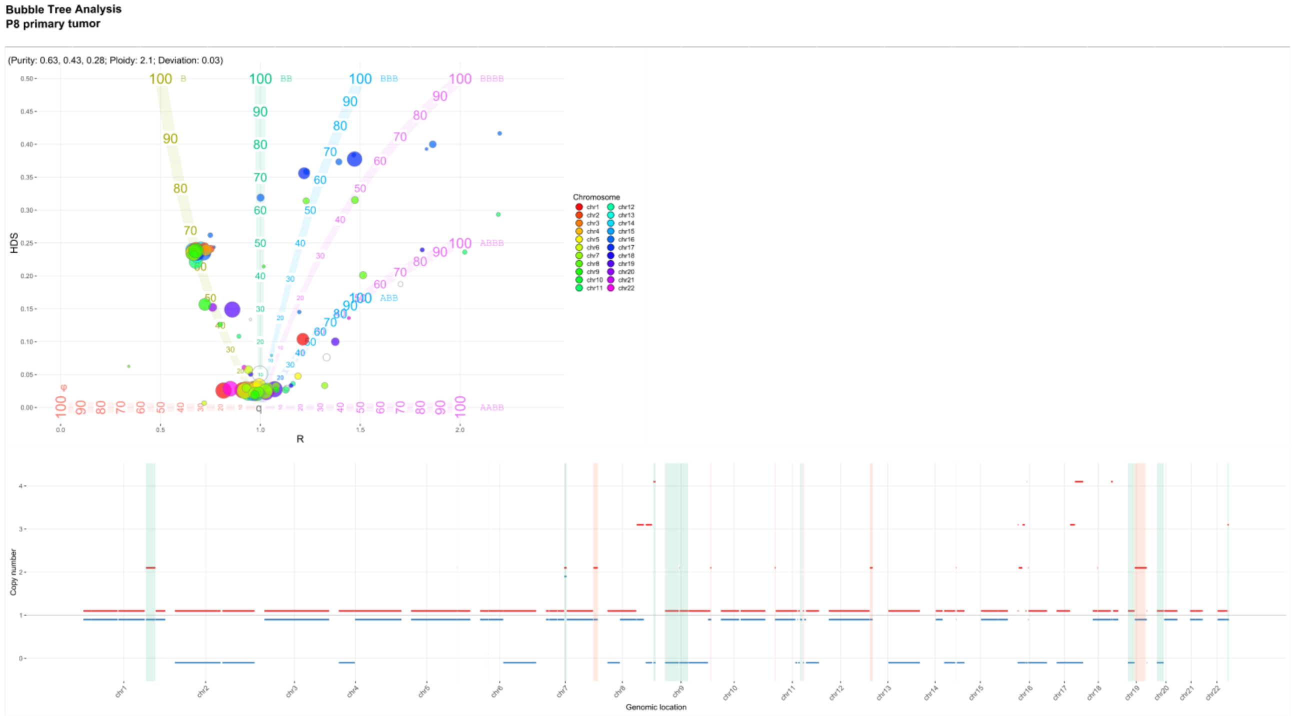

Supplementary Figure S8. BubbleTree analysis graph and copy number plot of P8 primary tumor. The BubbleTree analysis is based on exome sequencing data. In the upper panel, the *R*-HDS plot, the *R* score indicates the copy number ratio between the tumor and matched normal sample, and HDS is the heterozygous-deviation score. The tree branches represent the integer allele-specific copy numbers. The lower panel depicts the copy number events. The tumor cell content of each sample is estimated, indicated by the first figure in the purity estimate. The following figures represent the frequency of major subclones.

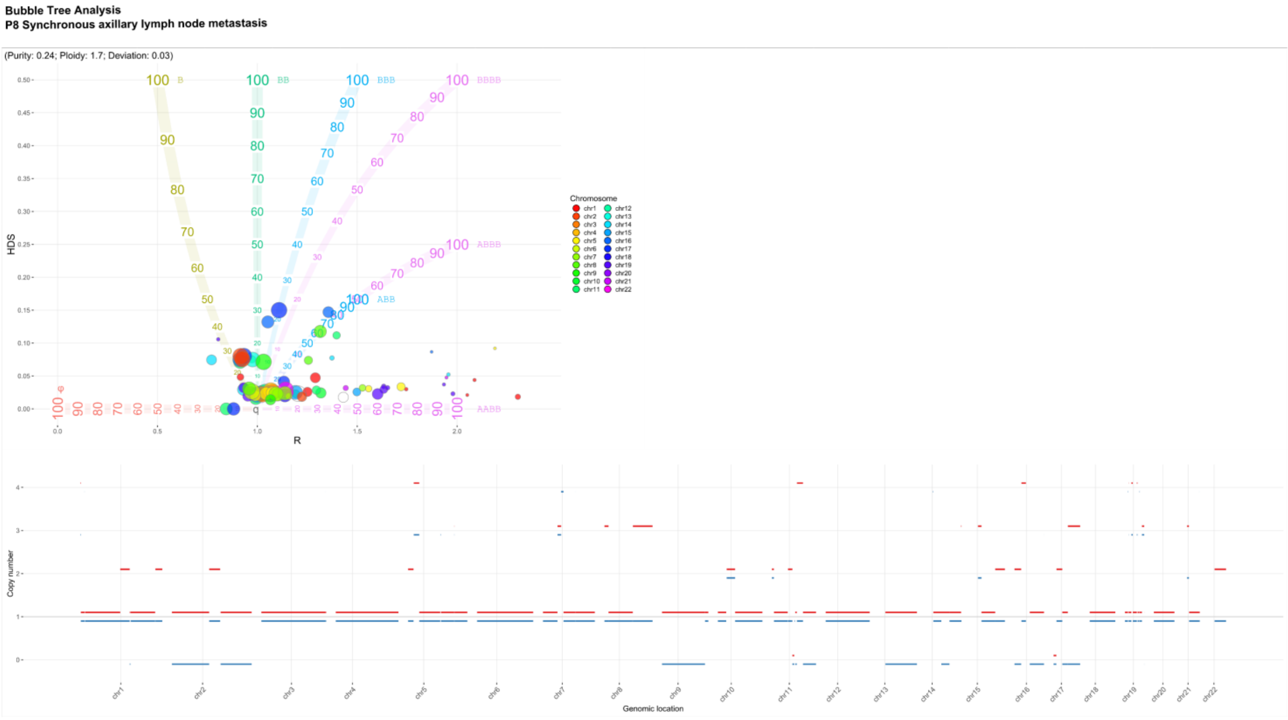

Supplementary Figure S9. BubbleTree analysis graph and copy number plot of P8 synchronous axillary lymph node metastasis. The BubbleTree analysis is based on exome sequencing data. In the upper panel, the *R*-HDS plot, the *R* score indicates the copy number ratio between the tumor and matched normal sample, and HDS is the heterozygous-deviation score. The tree branches represent the integer allele-specific copy numbers. The lower panel depicts the copy number events. The tumor cell content of each sample is estimated, indicated by the first figure in the purity estimate. The following figures represent the frequency of major subclones.

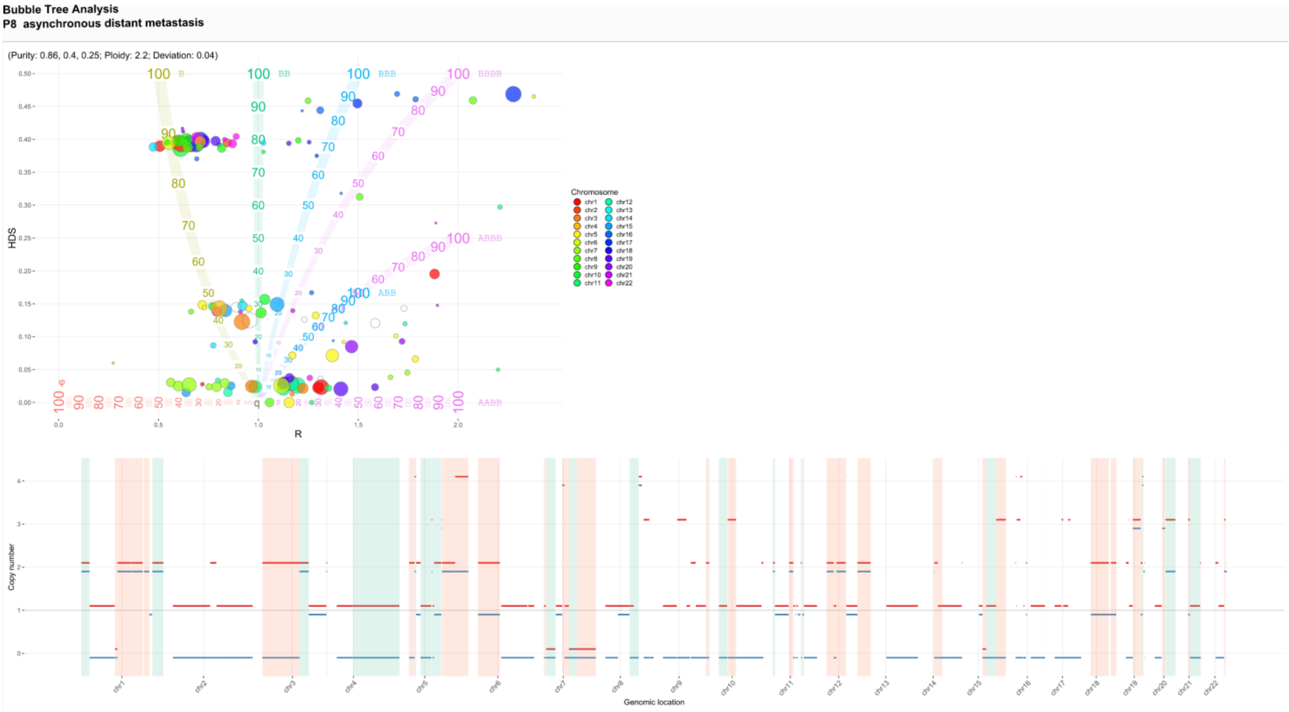

Supplementary Figure S10. BubbleTree analysis graph and copy number plot of P8 asynchronous distant metastasis. The BubbleTree analysis is based on exome sequencing data. In the upper panel, the *R*-HDS plot, the *R* score indicates the copy number ratio between the tumor and matched normal sample, and HDS is the heterozygous-deviation score. The tree branches represent the integer allele-specific copy numbers. The lower panel depicts the copy number events. The tumor cell content of each sample is estimated, indicated by the first figure in the purity estimate. The following figures represent the frequency of major subclones.

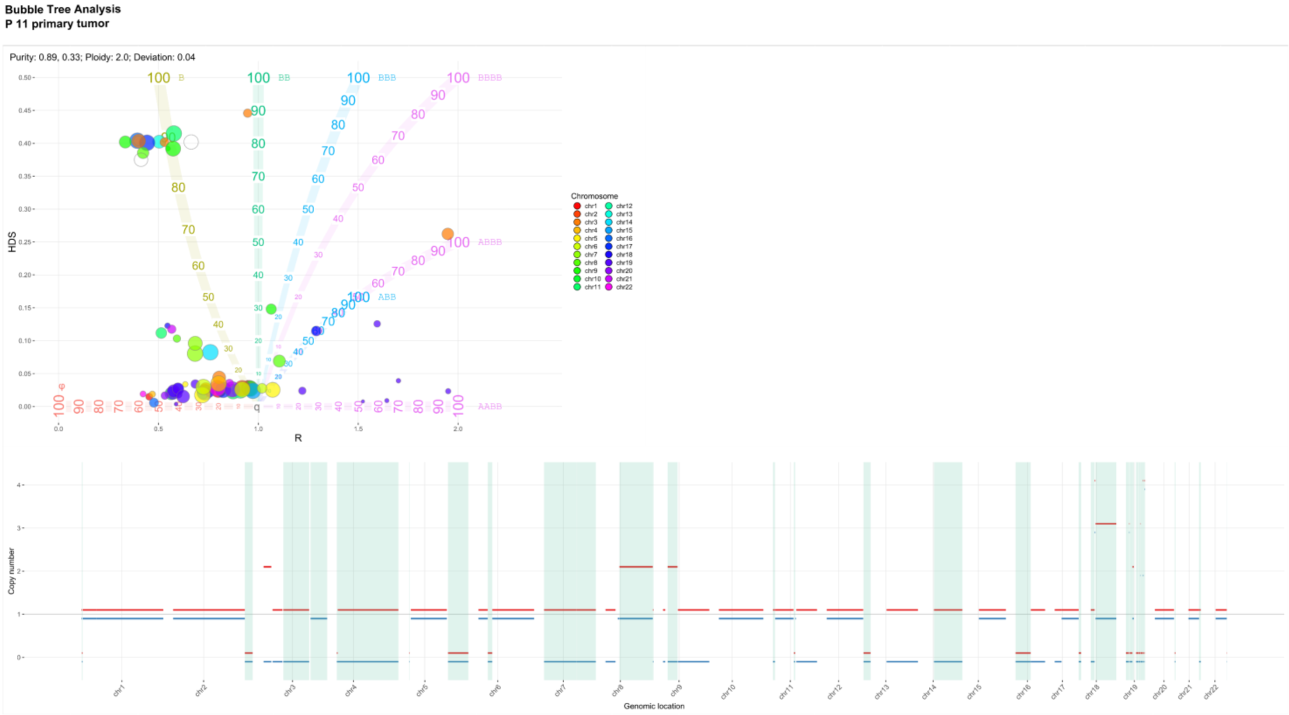

Supplementary Figure S11. BubbleTree analysis graph and copy number plot of P11 primary tumor. The BubbleTree analysis is based on exome sequencing data. In the upper panel, the *R*-HDS plot, the *R* score indicates the copy number ratio between the tumor and matched normal sample, and HDS is the heterozygous-deviation score. The tree branches represent the integer allele-specific copy numbers. The lower panel depicts the copy number events. The tumor cell content of each sample is estimated, indicated by the first figure in the purity estimate. The following figures represent the frequency of major subclones.

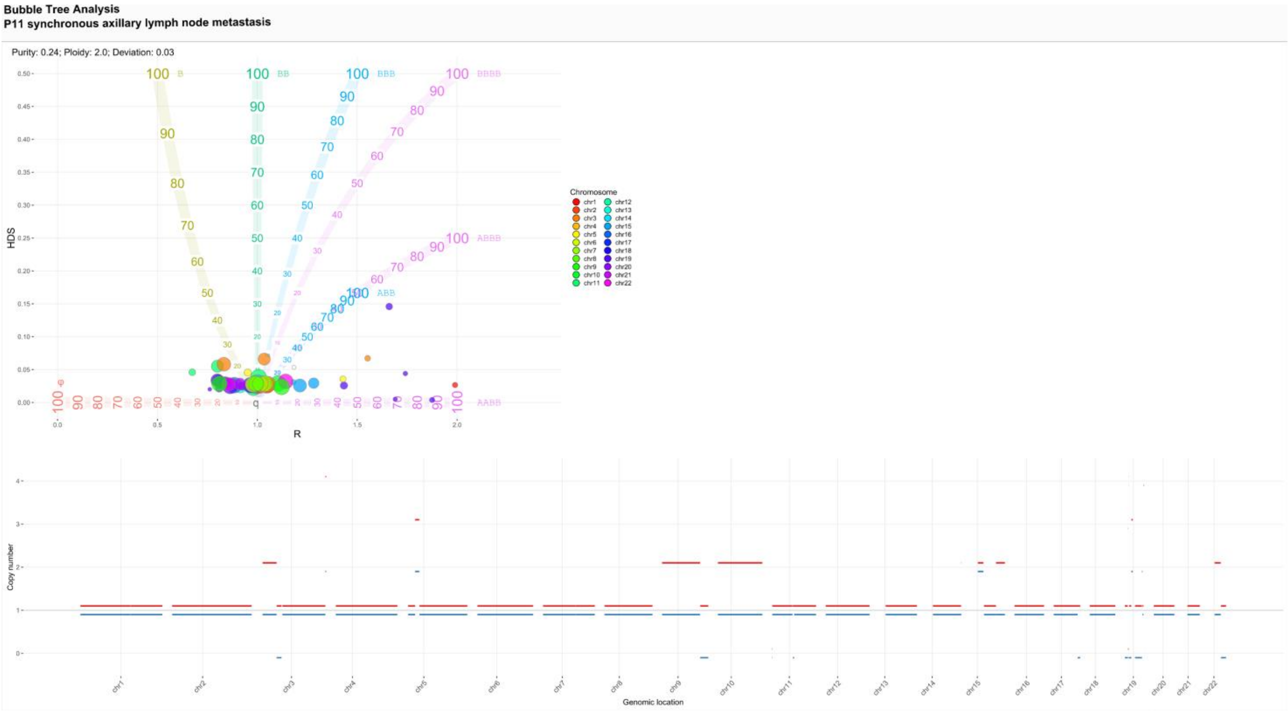

Supplementary Figure S12. BubbleTree analysis graph and copy number plot of P11 synchronous axillary lymph node metastasis. The BubbleTree analysis is based on exome sequencing data. In the upper panel, the *R*-HDS plot, the *R* score indicates the copy number ratio between the tumor and matched normal sample, and HDS is the heterozygous-deviation score. The tree branches represent the integer allele-specific copy numbers. The lower panel depicts the copy number events. The tumor cell content of each sample is estimated, indicated by the first figure in the purity estimate. The following figures represent the frequency of major subclones.

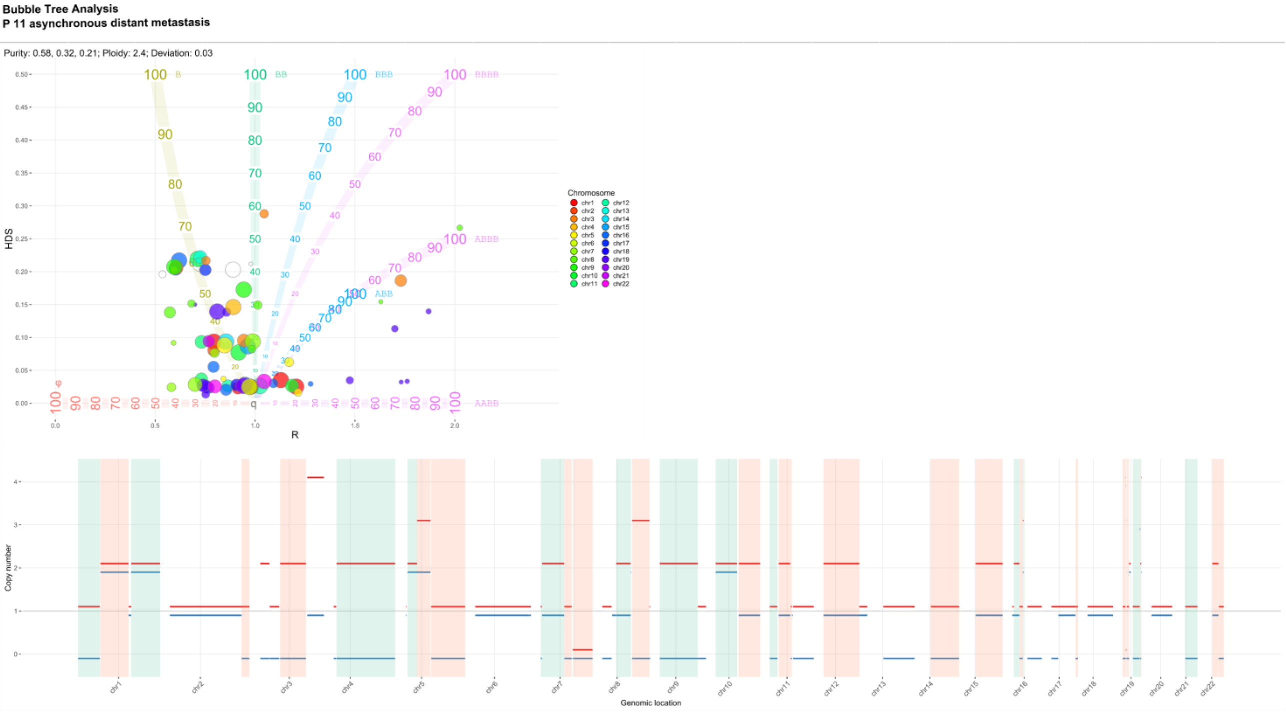

Supplementary Figure S13. BubbleTree analysis graph and copy number plot of P11 asynchronous distant metastasis. The Bubble Tree analysis is based on exome sequencing data. In the upper panel, the *R*-HDS plot, the *R* score indicates the copy number ratio between the tumor and matched normal sample, and HDS is the heterozygous-deviation score. The tree branches represent the integer allele-specific copy numbers. The lower panel depicts the copy number events. The tumor cell content of each sample is estimated, indicated by the first figure in the purity estimate. The following figures represent the frequency of major subclones.

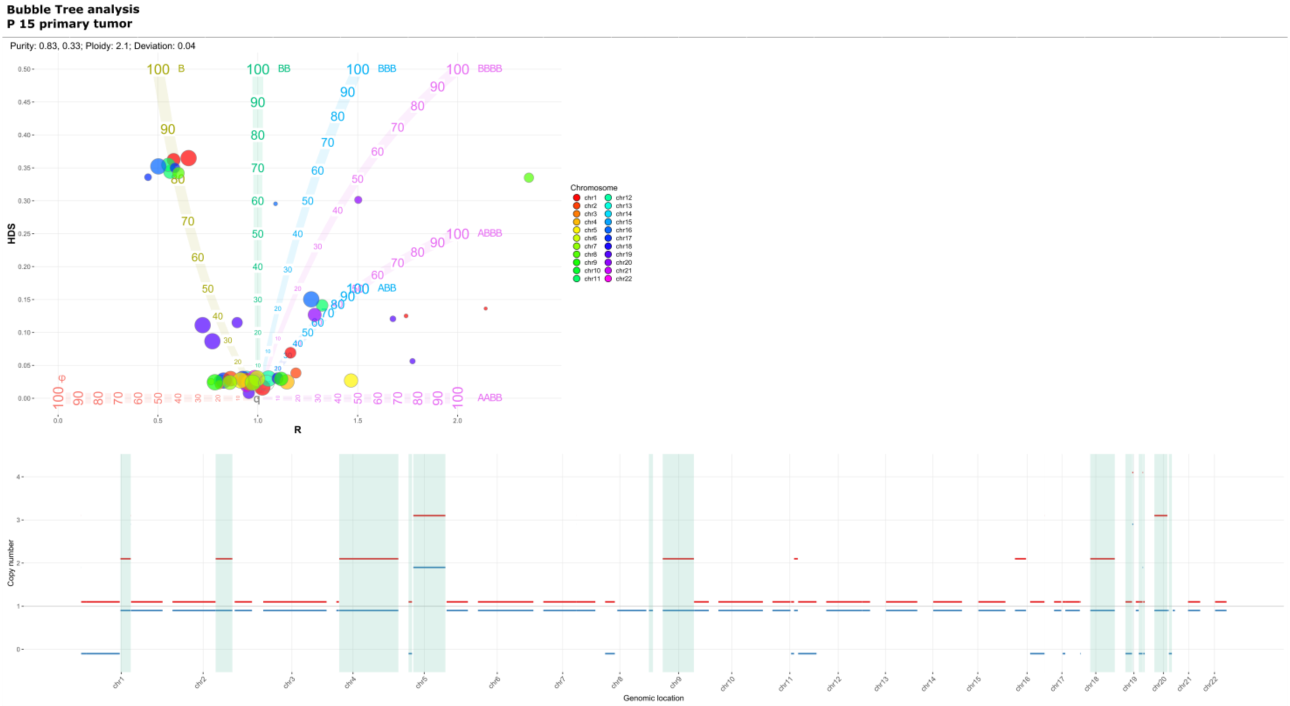

Supplementary Figure S14. BubbleTree analysis graph and copy number plot of P15 primary tumor. The BubbleTree analysis is based on exome sequencing data. In the upper panel, the *R*-HDS plot, the *R* score indicates the copy number ratio between the tumor and matched normal sample, and HDS is the heterozygous-deviation score. The tree branches represent the integer allele-specific copy numbers. The lower panel depicts the copy number events. The tumor cell content of each sample is estimated, indicated by the first figure in the purity estimate. The following figures represent the frequency of major subclones.

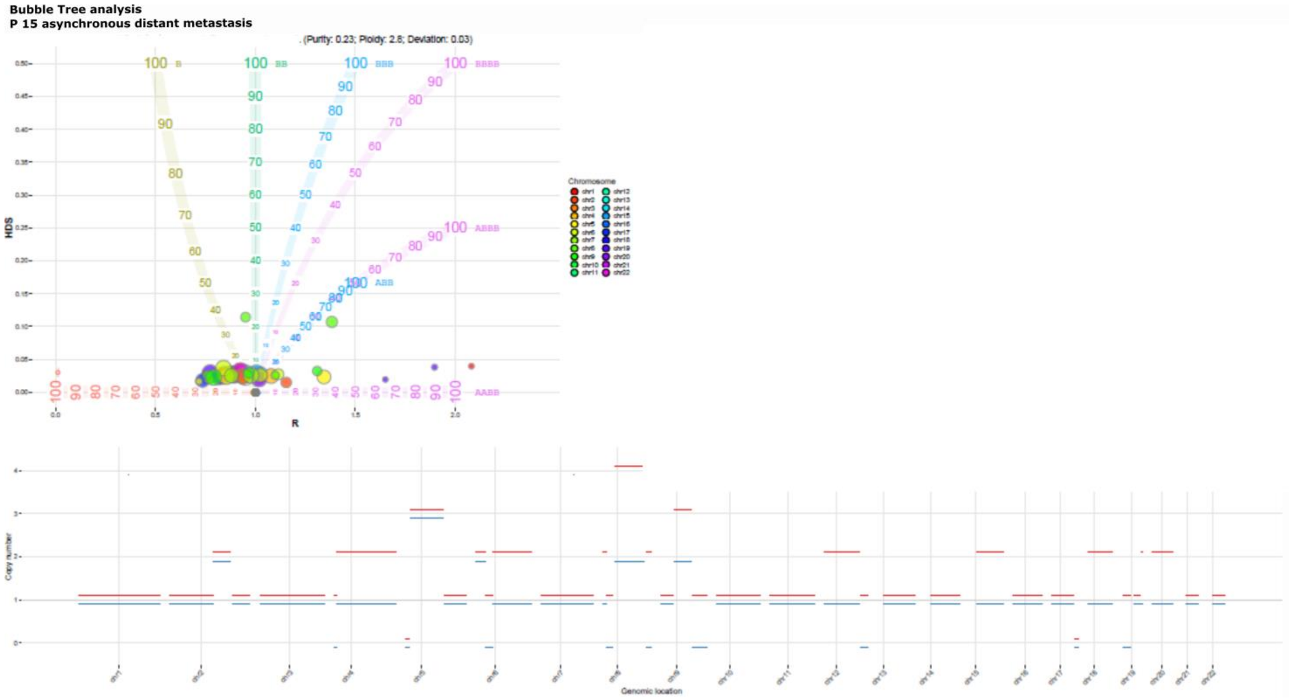

Supplementary Figure S15. BubbleTree analysis graph and copy number plot of P15 asynchronous distant metastasis. The BubbleTree analysis is based on exome sequencing data. In the upper panel, the *R*-HDS plot, the *R* score indicates the copy number ratio between the tumor and matched normal sample, and HDS is the heterozygous-deviation score. The tree branches represent the integer allele-specific copy numbers. The lower panel depicts the copy number events. The tumor cell content of each sample is estimated, indicated by the first figure in the purity estimate. The following figures represent the frequency of major subclones.

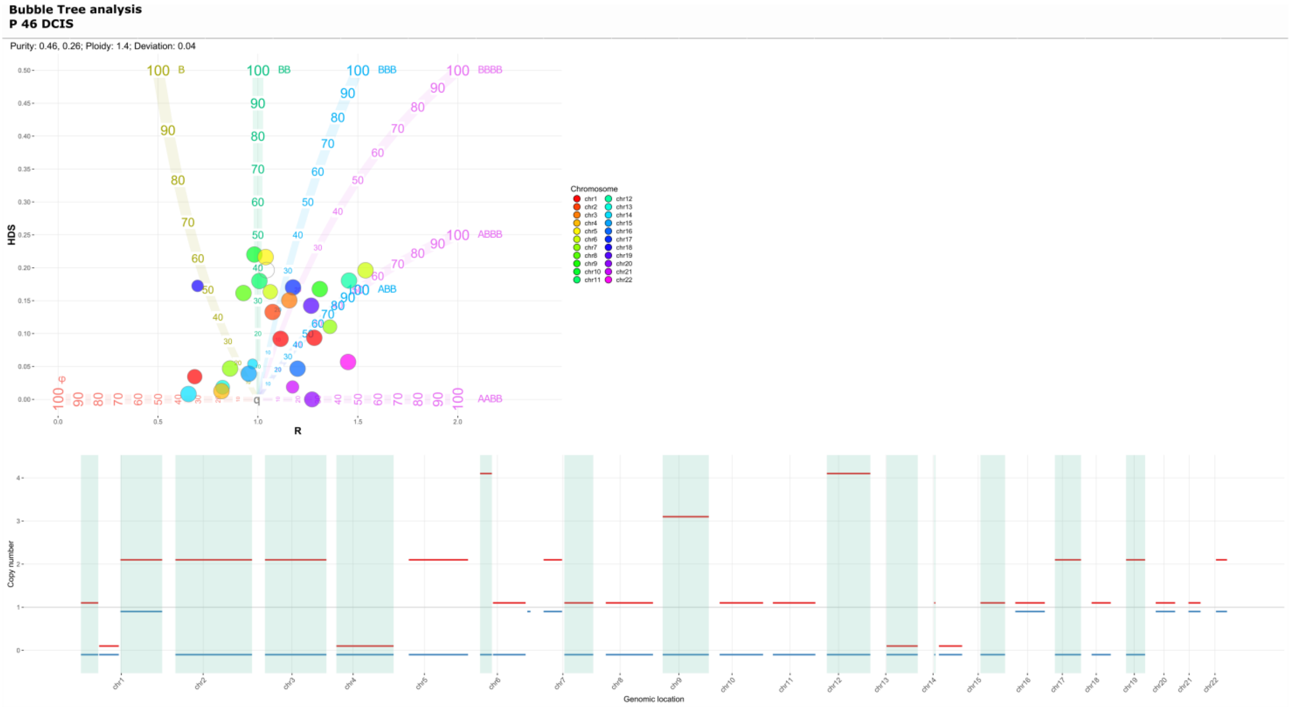

Supplementary Figure S16. BubbleTree analysis graph and copy number plot of P46 DCIS. The BubbleTree analysis is based on exome sequencing data. In the upper panel, the *R*-HDS plot, the *R* score indicates the copy number ratio between the tumor and matched normal sample, and HDS is the heterozygous-deviation score. The tree branches represent the integer allele-specific copy numbers. The lower panel depicts the copy number events. The tumor cell content of each sample is estimated, indicated by the first figure in the purity estimate. The following figures represent the frequency of major subclones.

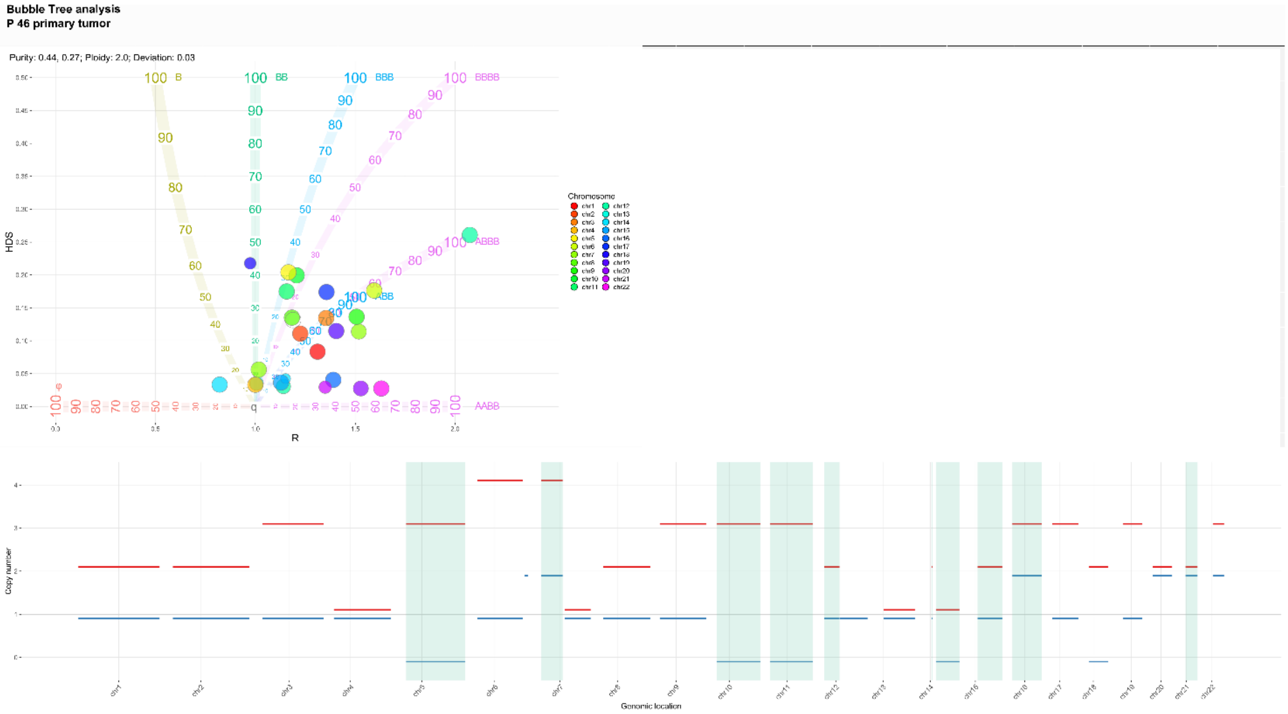

Supplementary Figure S17. BubbleTree analysis graph and copy number plot of P46 primary tumor. The BubbleTree analysis is based on exome sequencing data. In the upper panel, the *R*-HDS plot, the *R* score indicates the copy number ratio between the tumor and matched normal sample, and HDS is the heterozygous-deviation score. The tree branches represent the integer allele-specific copy numbers. The lower panel depicts the copy number events. The tumor cell content of each sample is estimated, indicated by the first figure in the purity estimate. The following figures represent the frequency of major subclones.

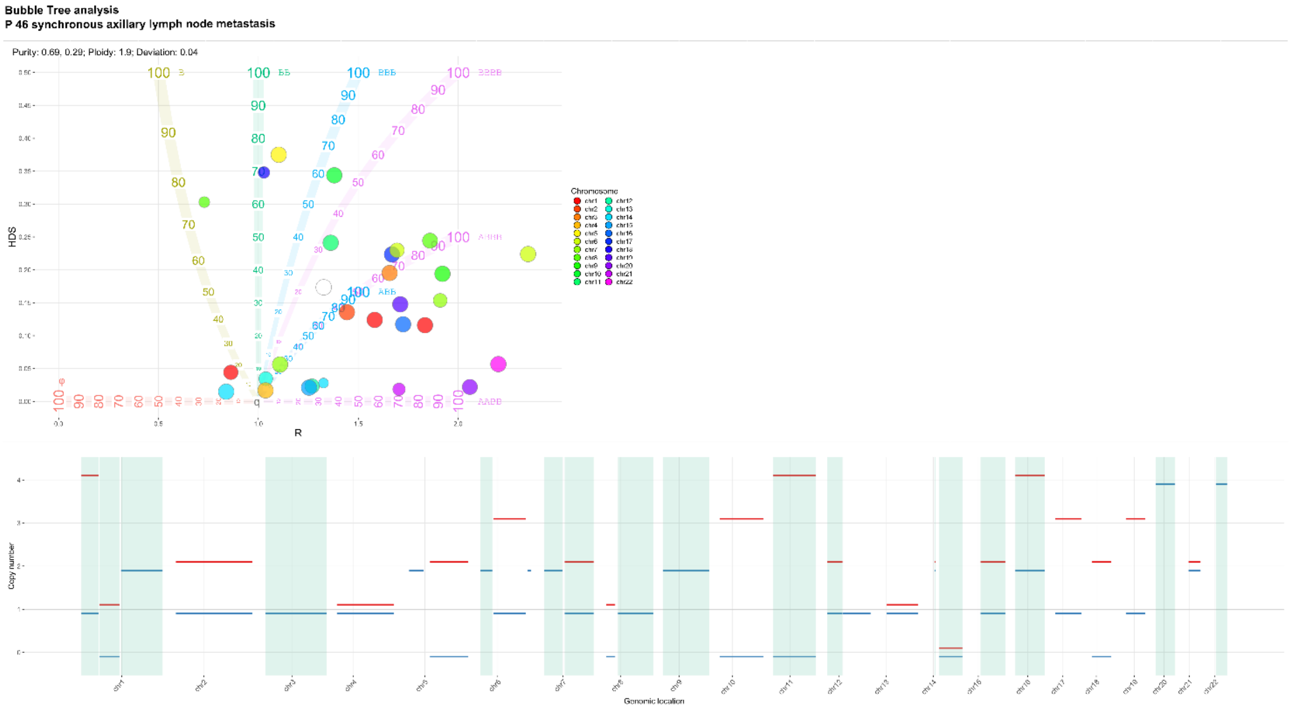

Supplementary Figure S18. BubbleTree analysis graph and copy number plot of P46 synchronous axillary lymph node metastasis. The BubbleTree analysis is based on exome sequencing data. In the upper panel, the *R*-HDS plot, the *R* score indicates the copy number ratio between the tumor and matched normal sample, and HDS is the heterozygous-deviation score. The tree branches represent the integer allele-specific copy numbers. The lower panel depicts the copy number events. The tumor cell content of each sample is estimated, indicated by the first figure in the purity estimate. The following figures represent the frequency of major subclones.

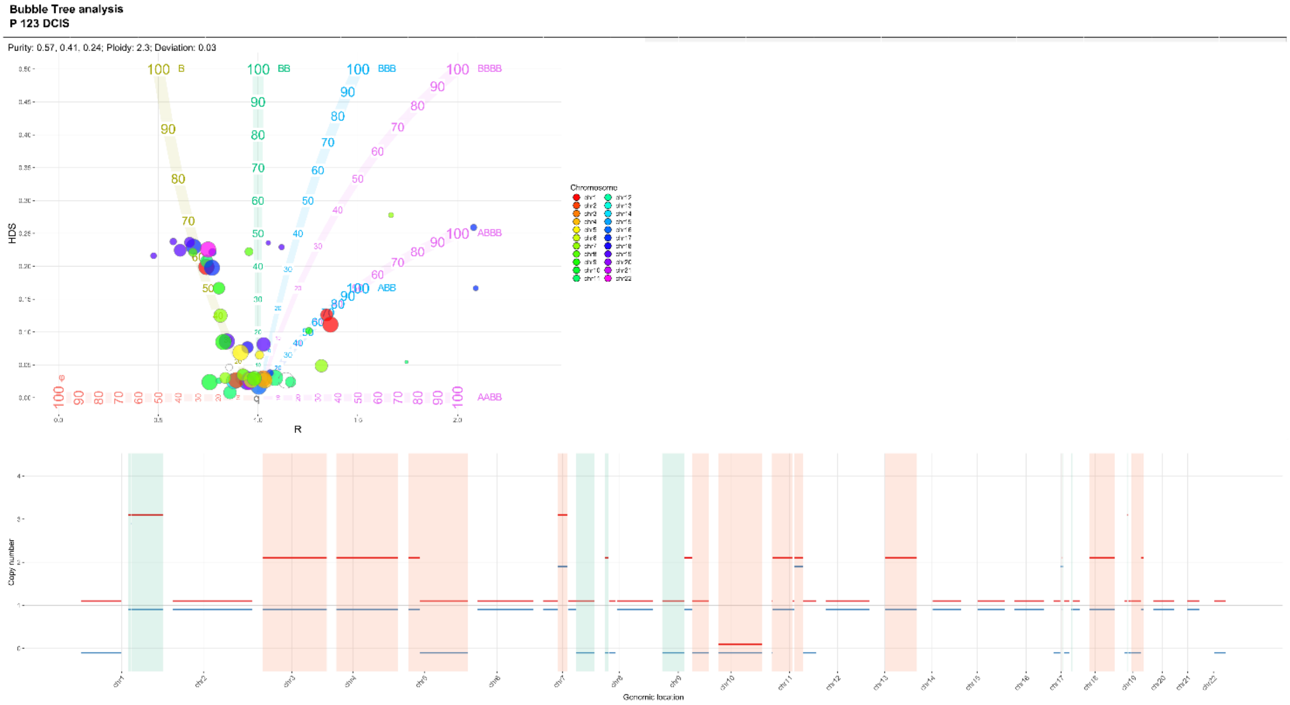

Supplementary Figure S19. BubbleTree analysis graph and copy number plot of P123 DCIS. The BubbleTree analysis is based on exome sequencing data. In the upper panel, the *R*-HDS plot, the *R* score indicates the copy number ratio between the tumor and matched normal sample, and HDS is the heterozygous-deviation score. The tree branches represent the integer allele-specific copy numbers. The lower panel depicts the copy number events. The tumor cell content of each sample is estimated, indicated by the first figure in the purity estimate. The following figures represent the frequency of major subclones.

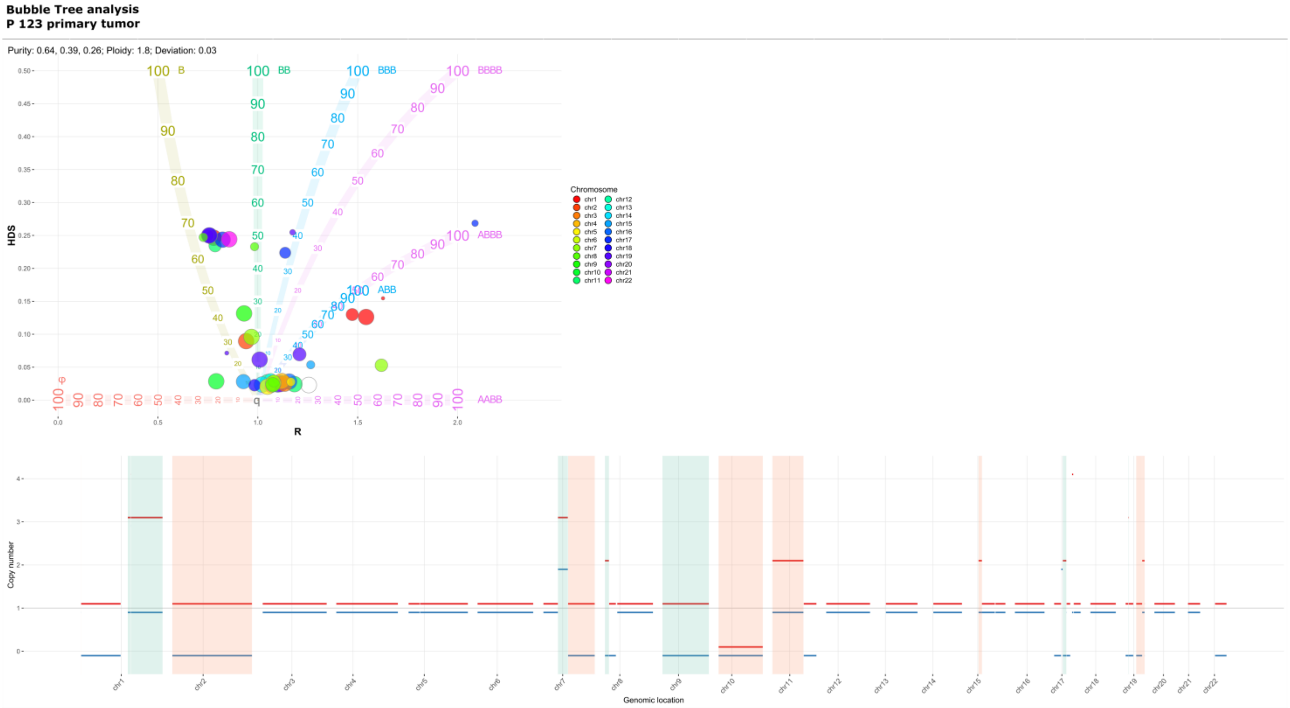

Supplementary Figure S20. BubbleTree analysis graph and copy number plot of P123 primary tumor. The BubbleTree analysis is based on exome sequencing data. In the upper panel, the *R*-HDS plot, the *R* score indicates the copy number ratio between the tumor and matched normal sample, and HDS is the heterozygous-deviation score. The tree branches represent the integer allele-specific copy numbers. The lower panel depicts the copy number events. The tumor cell content of each sample is estimated, indicated by the first figure in the purity estimate. The following figures represent the frequency of major subclones.

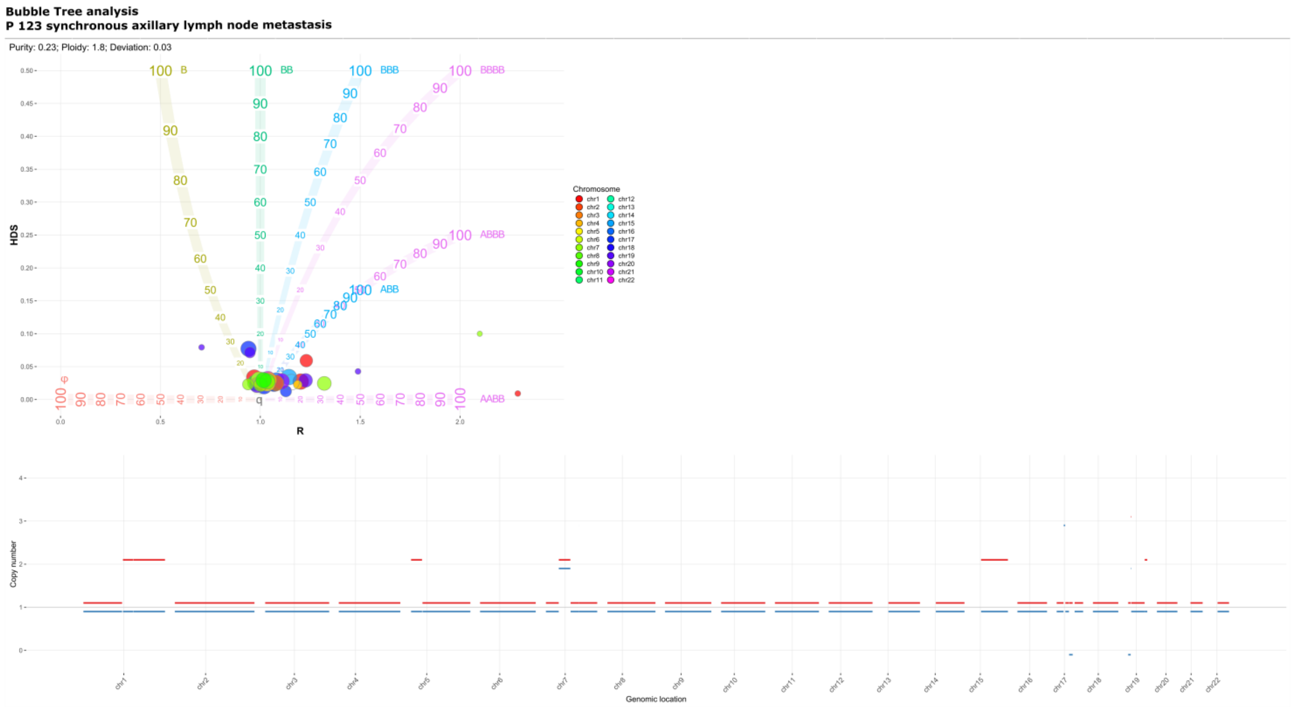

Supplementary Figure S21. BubbleTree analysis graph and copy number plot of P123 synchronous axillary lymph node metastasis. The BubbleTree analysis is based on exome sequencing data. In the upper panel, the *R*-HDS plot, the *R* score indicates the copy number ratio between the tumor and matched normal sample, and HDS is the heterozygous-deviation score. The tree branches represent the integer allele-specific copy numbers. The lower panel depicts the copy number events. The tumor cell content of each sample is estimated, indicated by the first figure in the purity estimate. The following figures represent the frequency of major subclones.

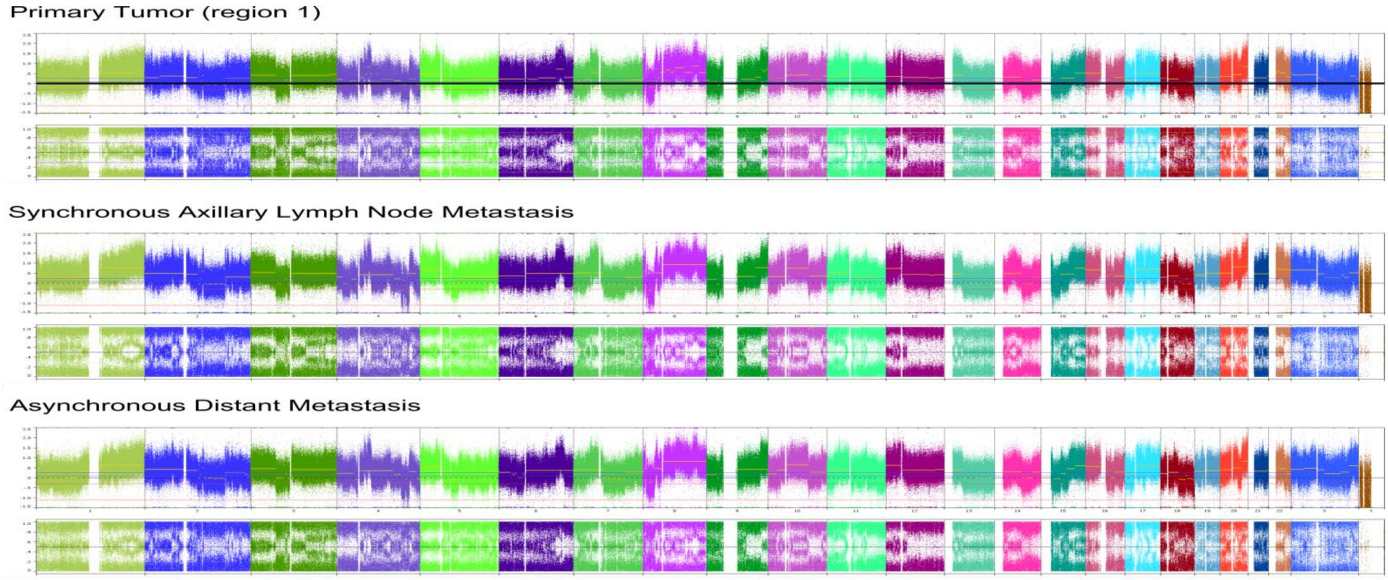

Supplementary Figure S22. SNP array tracks on the primary tumor (region 1), synchronous axillary lymph node metastasis and asynchronous distant metastasis of P4.

Primary Tumor (region 1)

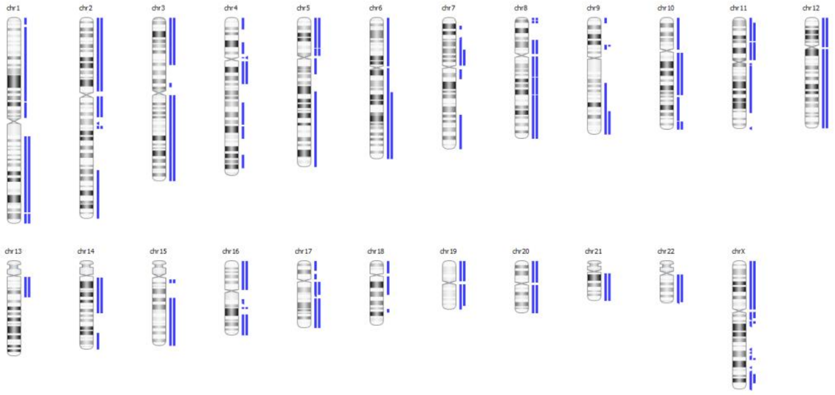

Synchronous Axillary Lymph Node Metastasis

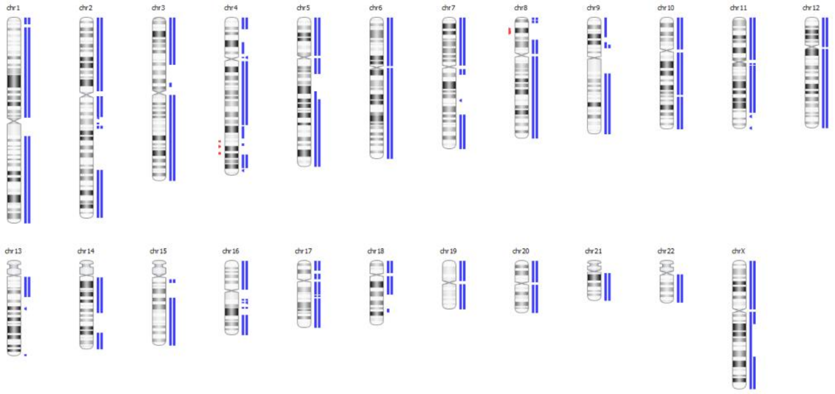

Asynchronous Distant Metastasis

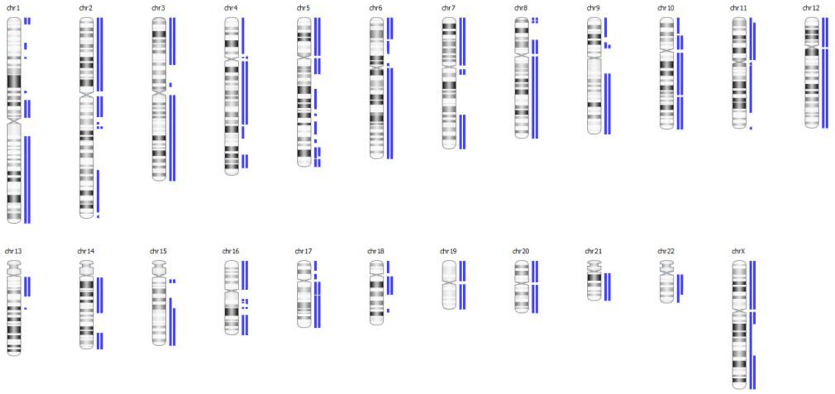

Supplementary Figure S23. Overview of copy number aberrations based on micro array analysis on the primary tumor (region 1), synchronous axillary lymph node metastasis and asynchronous distant metastasis of P4.

Supplementary Table S1. Patient characteristics. Age: age at diagnosis. IDC: Invasive ductal carcinoma. MG: Malignancy grade. ER: Estrogen receptor status. PR: Progesterone receptor status. N: normal. A: amplified. CEF: Cyclofosfamid, Epirubicin, 5- Flouracil. LN: lymph nodes.

**Pt ID**	**Age**	**Type**	**Primary tumor size**	**ER**	**PR**	**HER 2**	**MG**	**# pos LN**	**Treatment**
**P 4**	72 years	IDC	14 mm	pos	neg	A	II	1/10 LN	Surgery,<br>Adjuvant Letrozole.
**P 8**	58 years	IDC	50 mm	pos	pos	N	II	15/15	5 series of neo-adj. CEF. Surgery.<br>4 series of Taxotere/Gemcitabine.<br>Tamoxifen 2.5 years, then Arimidex.<br>Radiation therapy
**P 11**	46 years	IDC	25 mm	pos	pos	N	III	1/15 LN	Surgery. Adjuvant 7 series of CEF, Tamoxifen. Radiation therapy.
**P 15**	66 years	IDC	17 mm + 15 mm = multifokal	pos	pos	N	III	17/18 LN	Surgery. Adjuvant Letrozole.<br>Radiation therapy.
**P 46**	79 years	IDC	10 mm and diffusely spread 110 mm	pos	neg	N	III	5/20 LN	Neo-adj.<br>Letrozole. Surgery.
**P 123**	67 years	IDC	23 mm	pos	neg	A	II	5/11 LN	Surgery.

Supplementary Table S2. Exome sequencing data. GB: Gigabases.

Sample ID	Reads	Raw yield GB	Yield > Q30 GB	% duplicate reads	On target %	Mean quality score	Mean coverage	Tumor purity
**P 4 normal**	137998082	13.9	12.7	30.2	49	35	76.3	
**P 4 primary tumor region 1**	202241820	20.4	17.5	35.2	42	34	89.1	0.76
**P 4 primary tumor region 2**	214612358	21.69	18.45	37.6	40.8	34	89.0	0.68
**P 4 axillary LN metastasis**	228636662	23.1	19.7	37.7	41	34	95.3	0.95
**P 4 asynchronous metastasis**	182965674	18.5	15.9	36.2	42	34	79.4	0.84
**P 8 normal**	265430620	26.8	24.8	30.5	50	36	148.6	
**P 8 DCIS 1**	243928670	24.6	20.9	40.8	42	34	98.7	0.84
**P 8 DCIS 2**	215827498	21.8	18.4	38.1	41	34	89.2	0.74
**P 8 primary tumor**	157726216	15.9	14.5	39.2	48	35	74.2	0.63
**P 8 axillary LN metastastasis**	214122460	21.6	18.4	39.3	42	34	89.8	0.24
**P 8 asynchronous metastasis**	268675802	27.1	23.0	41.8	44	34	110.7	0.86
**P 11 normal**	140623584	14.2	13.0	40.2	48	35	64.9	
**P 11 primary tumor**	184097102	18.5	15.6	41.4	41	34	72.2	0.89
**P 11 axillary LN metastasis**	216085566	21.8	18.3	44.5	41	34	79.8	0.24
**P 11 asynchronous metastasis**	183796550	18.6	15.7	44.2	44	34	72.8	0.58
**P 15 normal**	141189470	14.2	13.1	55.1	48	35	49.5	

**P 15 primary tumor**	220176516	22.2	18.7	48.6	40	34	73.9	0.83
**P 15 asynchronous metastasis**	250377642	25.3	21.5	51.5	43	34	84.3	0.23
**P 46 normal**	25544032	2.5	2.3	41.4	48	35	11.5	
**046 DCIS**	161940808	16.4	13.4	40.8	42	33	65.5	0.46
**P 46 primary**	174537472	17.6	14.3	39.8	40	33	68.9	0.44
**P 46 axillary LN metastasis**	190894534	19.3	15.7	40.9	41	33	75.3	0.69
**P 123 normal**	147519622	14.9	12.8	61.2	48	34	45.0	
**P 123 DCIS**	193871748	19.6	16.5	44.4	43	34	74.8	0.57
**P 123 primary tumor**	224591476	22.6	19.3	48.4	44	34	83.4	0.64
**P 123 axillary LN metastasis**	246589864	24.9	20.9	48.2	44	34	91.7	0.23

Supplementary Table S3. Mean coverage of validated positions.

Sample ID	Mean coverage in validated positions
P 4 normal	407 x
P 4 primary tumor region 1	486 x
P 4 primary tumor region 2	458 x
P 4 axillary lymph node metastasis	457 x
P 4 asynchronous metastasis	441 x
P 8 normal	399 x
P 8 DCIS 1	493 x
P 8 DCIS 2	377 x
P 8 primary tumor	436 x
P 8 axillary lymph node metastasis	440 x
P 8 asynchronous metastasis	372 x
P 11 normal	317 x
P 11 primary tumor	592 x
P 11 axillary lymph node metastasis	548 x
P 11 asynchronous metastasis	569 x
P 15 normal	200 x
P 15 primary tumor	597 x
P 15 asynchronous metastasis	505 x
P 46 normal	384 x
P 46 DCIS	602 x
P 46 primary tumor	613 x
P 46 axillary lymph node metastasis	580 x
P 123 normal	463 x
P 123 DCIS	477 x
P 123 primary tumor	477 x
P 123 axillary lymph node metastasis	449 x

Supplementary Table S4. List of somatic mutations and allele frequencies for P4.

ALN: axillary lymph node; Ref: reference allele; Alt: alternative allele; RD: read depth; BAF: alternative allele frequency.

				Normal		Primary tumor 1		Primary tumor 2		ALN metastasis		Asynchronous metastasis	
**Chr**	**Start**	**Ref**	**Alt**	**RD**	**BAF**	**RD**	**BAF**	**RD**	**BAF**	**RD**	**BAF**	**RD**	**BAF**
1	9931281	G	C	186	0.00	144	**0.22**	125	0.15	73	**0.43**	111	**0.37**
1	19513032	A	T	373	0.00	170	**0.36**	199	**0.29**	88	**0.58**	136	**0.32**
1	25167343	G	C	533	0.00	263	**0.33**	262	**0.31**	132	**0.54**	189	**0.28**
1	25573168	CT	-	100	0.00	90	**0.23**	80	**0.16**	67	**0.37**	47	**0.40**
1	25573357	C	A	107	0.00	78	0.12	80	0.15	43	**0.34**	45	**0.32**
1	43897998	G	T	142	0.00	202	0.00	142	0.00	116	**0.17**	69	**0.22**
1	64644112	T	G	251	0.00	225	0.00	220	0.00	132	**0.24**	173	0.00
1	76215233	A	T	211	0.00	184	0.00	172	0.00	102	**0.24**	108	0.00
1	77094447	A	T	766	0.00	520	0.00	496	0.00	375	**0.27**	461	0.00
1	109856898	ATG	-	130	0.00	122	0.15	108	0.07	86	**0.21**	93	**0.18**
1	151060714	G	C	622	0.00	697	0.10	557	0.07	499	0.14	540	0.13
1	153312986	G	C	50	0.00	70	0.11	49	0.09	49	**0.28**	57	0.12
1	153657525	C	A	95	0.00	55	**0.47**	80	**0.32**	58	**0.44**	69	**0.34**
1	155631133	C	A	118	0.00	219	0.04	198	0.04	161	**0.19**	177	**0.15**
1	155721883	C	A	139	0.00	232	0.09	208	0.09	169	**0.21**	180	0.14
1	158227230	CTTCATCAT	-	753	0.00	780	0.08	825	0.07	643	0.12	830	0.08
1	161882118	C	A	760	0.00	458	**0.47**	462	**0.45**	393	**0.47**	421	**0.44**
1	169498936	C	T	1045	0.00	1269	0.09	1254	0.10	1233	0.12	1299	**0.17**
1	169498943	C	T	1036	0.00	1271	0.09	1247	0.10	1235	0.12	1294	**0.18**
1	177906564	-	C	138	0.00	201	**0.16**	202	0.09	154	**0.16**	183	**0.16**
1	180063424	C	A	614	0.00	576	0.09	457	0.05	414	0.14	366	0.13
1	202245585	GCCCTCAACCGCATCA	-	574	0.00	1067	0.06	942	0.06	1002	0.10	849	0.10

1	206329060	G	A	242	0.00	394	**0.29**	336	**0.25**	415	**0.25**	408	**0.22**
1	209803080	A	C	109	0.00	221	0.00	188	0.00	190	0.14	157	0.09
1	221875842	T	G	385	0.00	563	0.00	673	0.00	772	0.08	587	0.10
1	230846566	C	G	218	0.00	412	0.00	485	0.00	645	0.00	448	0.09
1	235318410	C	T	471	0.00	633	0.08	657	0.07	825	0.08	582	0.08
1	237895449	G	C	566	0.00	684	0.05	628	0.05	848	0.06	675	0.07
1	241850884	C	G	373	0.00	688	0.06	606	0.05	751	0.00	701	0.00
1	248616883	T	A	73	0.01	455	0.13	340	0.09	477	0.08	468	0.11
2	31624195	C	A	393	0.00	250	**0.17**	264	**0.16**	178	**0.30**	272	**0.21**
2	39417586	A	C	916	0.00	582	**0.40**	586	**0.35**	435	**0.45**	526	**0.36**
2	43980821	G	C	509	0.00	472	0.09	465	0.10	415	0.01	446	0.00
2	75928332	G	A	753	0.00	730	0.00	788	0.00	491	**0.18**	555	0.14
2	79385590	C	A	226	0.00	189	**0.44**	173	**0.35**	69	**0.70**	104	**0.59**
2	85982046	C	A	115	0.00	193	0.05	172	0.09	181	0.13	157	0.14
2	98351013	C	T	262	0.00	507	0.00	454	0.00	569	0.00	397	0.13
2	100209992	C	A	152	0.00	236	0.11	212	0.09	230	**0.16**	254	0.11
2	128480565	G	A	468	0.00	318	0.05	324	0.07	277	0.00	264	0.00
2	166897796	A	T	368	0.00	224	0.00	252	0.00	86	**0.45**	113	**0.36**
2	183608332	G	A	803	0.00	271	**0.50**	248	**0.51**	42	**0.86**	138	**0.66**
2	191300763	A	T	750	0.00	876	**0.24**	784	**0.28**	1003	**0.20**	808	**0.23**
2	197645297	G	C	897	0.00	794	0.12	763	0.11	623	**0.19**	642	**0.18**
2	206869381	G	T	654	0.00	515	**0.31**	436	**0.33**	220	**0.53**	297	**0.47**
2	210804286	G	A	447	0.00	510	0.00	410	0.00	225	**0.31**	257	**0.28**
2	233712215	-	CA<br>GC<br>AG<br>CA<br>GC<br>CG<br>CC<br>A	485	0.02	337	0.06	315	0.04	188	0.10	232	0.09
3	10379944	G	C	368	0.00	514	0.14	457	0.13	450	**0.16**	454	0.12
3	10953823	C	A	375	0.00	229	**0.44**	262	**0.32**	166	**0.55**	184	**0.46**
3	48802739	T	C	335	0.00	287	**0.15**	296	0.12	209	**0.19**	248	**0.16**
3	108672575	T	C	894	0.00	949	0.12	969	0.09	901	**0.16**	970	0.15
3	112358268	C	A	165	0.00	148	**0.35**	134	**0.41**	102	**0.50**	88	**0.60**
3	113085088	T	-	673	0.00	692	0.00	713	0.00	693	0.00	616	0.13
3	121409994	CT<br>GA<br>ATT<br>TG<br>TG<br>CT<br>GT<br>GA	-	788	0.00	781	0.04	741	0.03	536	0.08	559	0.07

		GACCTTTATTTT											
3	142142421	C	A	681	0.00	768	0.00	760	0.00	486	**0.19**	489	0.11
3	142681184	C	T	454	0.00	642	0.00	754	0.00	371	**0.18**	462	0.13
3	183027613	G	T	113	0.00	131	0.13	78	**0.20**	68	**0.26**	84	**0.18**
3	190322147	CTGTGGTT	-	802	0.00	1094	0.00	970	0.00	721	**0.15**	790	0.15
3	194362853	C	T	151	0.00	197	0.06	159	0.05	157	0.10	172	0.05
4	9217151	C	G	208	0.01	197	0.04	194	0.03	94	0.06	146	0.09
4	9246838	C	T	54	0.00	191	0.09	184	0.03	60	0.14	115	0.08
4	74365759	A	T	783	0.00	1519	0.13	1410	0.12	1766	0.14	1478	0.14
4	134073498	G	A	170	0.00	109	**0.17**	116	0.13	72	**0.26**	74	**0.35**
4	146807258	AAAGGGCACAGCTGGCACTG	-	438	0.00	208	0.13	234	0.13	170	**0.18**	183	**0.16**
5	56161670	-	CAGAAATATCACAGTAGGCGTAG	793	0.00	618	**0.20**	605	**0.16**	364	**0.33**	425	**0.30**
5	77406165	C	A	1165	0.00	679	**0.16**	703	**0.17**	361	**0.34**	491	**0.17**
5	132070016	G	T	865	0.00	531	**0.17**	504	0.15	351	**0.29**	464	**0.23**
5	137498994	C	A	624	0.00	646	0.00	548	0.00	481	0.02	413	0.13
5	140346922	G	T	437	0.00	315	**0.21**	331	**0.16**	260	**0.28**	335	**0.20**
5	140710941	TCAGGTGGTG	-	201	0.00	224	0.14	169	0.13	144	**0.23**	189	0.12

		GA											
5	149636152	A	T	89	0.00	89	0.09	85	**0.16**	80	**0.25**	72	**0.15**
5	149636153	C	A	86	0.00	91	0.09	87	**0.16**	83	**0.23**	73	**0.15**
6	24563659	T	G	325	0.00	384	0.00	406	0.00	368	**0.18**	247	0.15
6	26199103	A	C	456	0.00	338	0.10	345	0.06	247	**0.21**	219	**0.22**
6	30126306	CTC<br>TCC<br>AA<br>TT<br>GT<br>GC<br>TA<br>AG<br>AG<br>GA<br>TG<br>CTC<br>TG<br>CT<br>GT<br>TCC<br>T	-	228	0.00	175	0.08	176	0.07	155	0.09	161	0.05
6	33235910	G	C	314	0.00	135	**0.42**	195	**0.20**	100	**0.51**	130	**0.37**
6	36181973	G	C	378	0.00	360	0.14	369	0.13	292	**0.18**	295	**0.18**
6	42618064	AG<br>AA<br>TG<br>TG<br>CC<br>AA	-	733	0.00	497	0.10	469	0.08	349	**0.18**	385	**0.16**
6	52268463	T	G	367	0.00	296	0.07	301	0.08	202	**0.22**	175	**0.27**
6	158504597	T	G	359	0.00	338	**0.21**	334	**0.19**	213	**0.44**	206	**0.43**
7	12400990	A	G	369	0.00	315	**0.18**	246	**0.17**	282	**0.18**	290	**0.19**
7	44579562	A	G	628	0.00	966	**0.15**	886	0.12	1385	0.11	1053	0.12
7	48317903	G	-	480	0.00	481	0.00	413	0.00	535	0.12	408	0.11
7	92763549	C	A	561	0.00	222	**0.24**	242	**0.15**	137	**0.33**	172	**0.30**
7	128486393	G	T	320	0.00	224	**0.35**	274	**0.30**	153	**0.60**	167	**0.50**
7	150707707	G	-	172	0.00	117	**0.23**	145	**0.18**	110	**0.31**	158	**0.16**
7	154677353	G	A	283	0.00	249	**0.19**	215	**0.19**	164	**0.34**	202	**0.33**
8	7718203	C	T	279	0.00	726	0.05	640	0.04	496	0.12	501	0.15
8	19813429	C	G	362	0.00	147	**0.29**	119	**0.27**	14	**0.80**	55	**0.51**
8	27288975	A	T	614	0.00	256	**0.63**	311	**0.56**	42	**0.92**	126	**0.77**
8	28017971	C	G	297	0.00	213	**0.25**	187	**0.24**	164	**0.23**	198	**0.19**
8	31498189	T	C	89	0.00	161	0.10	181	0.09	163	0.06	152	0.13
8	57079853	G	A	319	0.00	539	0.00	479	0.00	522	0.09	566	0.11
8	65517396	G	T	446	0.00	559	0.06	534	0.09	474	0.12	536	0.12

8	105507409	C	T	481	0.00	359	**0.35**	317	**0.35**	362	**0.38**	351	**0.34**
8	108276514	T	A	428	0.00	536	0.04	468	0.07	532	0.09	492	0.08
8	117782582	C	G	578	0.00	1247	0.06	1063	0.05	1306	0.06	1478	0.05
8	124790306	C	G	690	0.00	826	**0.38**	762	**0.37**	891	**0.43**	924	**0.48**
8	143994065	G	A	258	0.01	402	0.12	340	0.14	442	**0.17**	384	**0.16**
8	144658984	GGGCCCTCACCAGCTGCTGGC	-	152	0.00	222	**0.17**	222	0.12	278	**0.18**	254	0.11
8	145676029	G	T	83	0.00	202	0.00	156	0.00	184	0.12	153	0.09
9	13190129	C	T	820	0.00	511	**0.17**	489	0.12	381	**0.23**	433	**0.15**
9	73235147	C	A	230	0.00	260	0.00	218	0.00	110	**0.32**	181	**0.24**
10	24874639	T	A	533	0.00	636	0.07	654	0.10	630	0.13	546	0.13
10	24874640	C	A	537	0.00	639	0.06	663	0.09	637	0.13	545	0.13
10	26414428	G	A	537	0.00	332	**0.25**	305	**0.30**	235	**0.43**	201	**0.48**
10	50028405	A	C	129	0.00	132	**0.16**	122	**0.22**	179	**0.19**	175	**0.19**
10	50819511	G	A	213	0.00	359	0.10	327	0.11	472	0.15	433	**0.16**
10	71392660	C	G	358	0.00	477	**0.20**	492	**0.20**	525	**0.35**	491	**0.33**
10	73450249	C	T	220	0.00	249	0.06	206	0.09	316	0.09	300	0.07
10	115950770	G	C	458	0.00	364	**0.19**	389	**0.16**	204	**0.31**	281	**0.22**
10	118137974	G	T	1027	0.00	767	**0.17**	681	0.13	469	**0.24**	438	**0.21**
10	124458861	C	T	360	0.00	683	0.08	701	0.06	549	0.13	513	0.08
10	125601974	C	G	286	0.00	511	0.00	488	0.00	281	0.11	278	0.11
11	62298281	T	C	541	0.00	404	**0.18**	379	**0.19**	277	**0.30**	316	**0.20**
11	62482998	C	T	549	0.00	420	**0.16**	464	**0.17**	286	**0.31**	334	**0.20**
11	67223661	C	A	99	0.00	102	0.00	131	0.00	108	0.00	111	0.06
11	68529105	A	C	571	0.00	351	**0.19**	394	0.13	300	**0.29**	292	**0.20**
11	76893486	G	T	320	0.00	366	0.00	379	0.00	315	0.00	284	**0.26**
11	85436215	C	T	340	0.00	427	0.00	358	0.00	329	0.00	276	0.14
11	94862108	C	T	522	0.00	409	**0.20**	334	**0.18**	246	**0.31**	308	**0.21**
11	113196360	G	A	644	0.00	411	**0.23**	396	**0.19**	296	**0.30**	331	**0.22**
11	118343220	G	C	533	0.00	173	**0.51**	220	**0.45**	27	**0.86**	92	**0.62**
11	118984804	GGACGAGTTGGC	-	306	0.00	153	**0.33**	205	**0.28**	46	**0.59**	89	**0.45**

		GC TAT CC CT											
11	125885225	A	T	840	0.00	387	**0.28**	416	**0.18**	220	**0.38**	292	**0.32**
12	6471284	G	C	204	0.00	269	0.00	262	0.00	301	**0.15**	320	0.11
12	7172533	T	C	686	0.00	584	0.14	602	0.10	808	0.14	761	0.15
12	14849186	C	G	490	0.00	475	0.00	428	0.00	637	0.00	500	0.08
12	48379588	G	C	208	0.00	312	0.12	325	0.11	266	**0.19**	291	**0.16**
12	53167409	T	C	480	0.00	614	0.00	550	0.00	419	**0.23**	468	0.12
12	54796835	C	G	143	0.00	130	0.08	130	0.12	103	**0.25**	111	**0.17**
12	67698443	G	A	446	0.02	374	**0.15**	330	**0.16**	266	**0.23**	299	**0.18**
12	105282898	T	C	940	0.00	721	0.13	685	0.13	561	**0.20**	632	**0.18**
12	112187007	T	C	216	0.00	182	0.14	184	0.08	192	**0.21**	182	**0.20**
13	32954219	T	-	658	0.00	938	0.10	862	0.08	690	**0.16**	674	0.14
13	32954222	C	A	648	0.00	916	0.10	857	0.08	665	**0.17**	640	0.14
14	20898268	C	G	236	0.00	232	**0.17**	213	**0.17**	171	**0.19**	225	**0.19**
14	31618327	C	A	334	0.00	283	0.12	234	0.09	226	**0.18**	204	**0.19**
14	32559937	C	T	822	0.00	454	**0.37**	468	**0.25**	296	**0.42**	256	**0.47**
14	35873753	C	T	367	0.00	388	**0.16**	461	0.10	520	0.13	323	**0.17**
14	45432426	G	C	667	0.00	695	0.10	682	0.12	695	0.11	487	**0.16**
14	50459522	G	T	490	0.00	465	0.13	454	0.10	457	**0.16**	399	0.11
14	52949670	A	C	1210	0.00	136 7	0.13	1146	0.12	1054	**0.17**	1029	**0.18**
14	77274414	-	CT GA GG GC CA TCT CCT GC	134	0.00	67	0.12	83	0.11	40	**0.33**	56	**0.21**
15	65256052	G	T	276	0.00	184	**0.28**	193	**0.20**	196	**0.30**	172	**0.29**
15	66679726	C	A	240	0.00	236	**0.18**	266	0.10	291	**0.16**	257	0.13
15	74427056	C	A	137	0.00	274	0.00	249	0.00	305	0.00	304	0.11
15	75656966	C	A	204	0.00	331	0.08	296	0.08	334	**0.16**	338	0.12
15	76646466	C	T	573	0.00	768	**0.19**	756	**0.16**	718	**0.32**	721	**0.28**
15	88420267	A	T	134	0.00	180	**0.21**	157	**0.22**	150	**0.38**	190	**0.27**
15	91488157	C	T	244	0.00	349	0.12	343	0.14	372	0.11	374	0.13
15	92663761	G	A	152	0.00	221	**0.23**	163	**0.29**	248	**0.21**	184	**0.29**
15	101555577	A	G	435	0.00	740	**0.21**	603	**0.20**	658	**0.25**	597	**0.23**
16	1263904	G	T	113	0.00	152	0.07	200	0.10	197	0.09	211	0.11
16	2855024	C	G	127	0.00	125	**0.29**	134	**0.31**	110	**0.34**	130	**0.25**
16	3639305	C	T	117	0.00	156	0.11	177	0.13	174	0.14	164	0.12
16	3786740	G	T	348	0.00	514	0.00	478	0.00	343	**0.15**	384	**0.16**

16	29841949	C	G	113	0.00	156	0.09	165	0.14	187	0.09	186	0.12
16	66434820	G	A	334	0.00	372	0.00	362	0.00	362	0.00	377	0.06
16	68011882	G	A	365	0.00	98	**0.69**	162	**0.56**	31	**0.90**	57	**0.81**
16	89623418	G	A	330	0.00	424	0.04	357	0.08	331	**0.16**	298	0.15
17	4841102	GG TG TG GG GG CC CA GG CG	-	705	0.00	550	0.13	683	0.12	484	**0.18**	566	**0.17**
17	6330276	G	C	74	0.00	39	**0.43**	49	**0.37**	32	**0.60**	35	**0.47**
17	6930145	CTT TG TA CA GG TA CG GA GC CA GC CTC C	-	127	0.00	108	0.00	97	0.00	68	0.12	87	0.10
17	29220795	G	C	945	0.00	837	**0.25**	892	**0.20**	719	**0.38**	693	**0.39**
17	36483817	T	C	361	0.00	507	0.09	469	0.11	455	**0.26**	545	0.12
17	37263703	G	A	147	0.00	286	0.10	195	0.10	275	0.11	238	0.12
17	38122115	A	T	218	0.00	469	0.00	368	0.00	497	0.09	424	0.08
17	39019544	G	T	186	0.00	424	0.08	463	0.08	468	0.09	421	0.12
17	39538239	A	T	181	0.00	376	0.10	353	0.07	324	0.09	369	0.12
17	43006259	C	A	189	0.00	674	0.00	617	0.00	554	0.08	470	0.10
17	78305836	CA	-	446	0.00	592	0.09	493	0.10	579	**0.18**	566	**0.18**
18	9279577	C	T	407	0.00	236	0.08	248	0.06	211	0.00	166	0.00
18	11610490	T	A	280	0.01	308	0.06	284	0.05	299	0.04	273	0.03
18	13055846	C	T	533	0.00	439	0.13	448	0.09	342	**0.18**	342	**0.17**
18	21141429	GG AG TCC CA GG G	-	330	0.00	258	0.13	241	0.14	195	**0.16**	200	**0.16**
18	31226270	A	G	798	0.00	441	**0.17**	417	0.14	283	**0.28**	325	**0.25**
18	43314354	A	T	209	0.00	145	0.00	152	0.00	61	**0.40**	67	**0.20**

19	5245852	T	C	365	0.00	498	0.05	485	0.09	406	**0.21**	397	**0.21**
19	6381736	C	G	429	0.00	521	0.00	599	0.00	579	**0.18**	571	**0.17**
19	14569112	G	T	76	0.00	108	**0.16**	127	0.07	81	**0.17**	74	**0.28**
19	16890209	G	T	192	0.00	267	0.00	325	0.00	268	0.11	247	0.14
19	18305657	C	G	134	0.00	143	0.00	181	0.00	159	**0.16**	175	**0.16**
19	36046599	G	A	437	0.00	532	**0.16**	489	0.14	646	**0.18**	552	**0.17**
19	37733955	C	G	449	0.00	291	0.14	272	0.11	256	0.15	284	0.13
19	40321141	C	G	195	0.00	199	0.09	208	0.13	170	**0.20**	189	**0.15**
19	41084386	G	T	462	0.01	399	0.07	386	0.10	360	**0.21**	291	**0.23**
19	43234070	C	A	346	0.00	434	0.00	425	0.00	308	**0.25**	324	**0.21**
19	47865906	G	T	315	0.01	331	0.07	363	0.09	259	**0.25**	246	**0.22**
19	52569634	C	A	506	0.00	334	0.15	251	0.15	248	**0.18**	239	**0.15**
19	52693377	C	A	223	0.00	219	0.00	311	0.00	292	0.00	209	**0.21**
19	53762223	G	A	286	0.00	311	0.00	292	0.00	279	0.00	192	**0.20**
20	43653679	G	A	662	0.00	632	0.12	584	0.10	580	0.13	474	0.14
20	47739670	T	C	303	0.00	405	0.00	382	0.00	341	0.00	256	**0.19**
20	49458391	C	A	205	0.00	472	0.11	482	0.07	608	0.10	495	0.10
20	58559759	C	A	1170	0.00	959	**0.61**	987	**0.57**	820	**0.68**	796	**0.66**
21	30313570	A	T	796	0.00	631	0.00	651	0.00	479	**0.20**	501	**0.20**
21	33711193	G	T	321	0.00	374	0.12	314	0.14	380	**0.15**	324	0.11
21	36206827	-	A	171	0.00	100	**0.28**	97	**0.29**	91	**0.41**	73	**0.32**
22	50277979	AG	-	459	0.00	501	0.01	436	0.08	412	0.00	342	0.00
22	50893771	A	C	220	0.00	231	0.00	235	0.00	277	0.00	154	0.11
X	2951334	A	T	206	0.00	279	0.11	228	0.14	271	**0.18**	317	0.00
X	19392690	G	T	315	0.00	487	0.00	416	0.00	334	**0.18**	260	**0.15**
X	24006642	T	A	598	0.00	768	0.09	768	0.03	598	**0.19**	442	**0.26**
X	44922988	C	G	651	0.00	576	0.08	589	0.06	643	0.00	576	0.00
X	47918729	C	T	472	0.00	495	0.13	427	0.12	496	0.13	485	0.14
X	48792107	T	A	616	0.00	733	0.03	779	0.04	817	0.13	754	0.13
X	48925416	T	A	89	0.00	132	0.12	146	0.13	128	**0.16**	145	0.12
X	125954893	A	T	151	0.00	146	0.00	161	0.01	128	0.01	127	**0.23**
X	133379549	A	T	643	0.00	378	0.10	299	0.11	244	**0.25**	271	**0.21**
X	135443707	G	T	660	0.00	483	0.13	445	0.10	367	0.00	545	0.00
X	152138030	T	A	537	0.00	530	0.00	551	0.00	430	**0.20**	593	**0.16**
X	152482856	G	T	234	0.00	211	**0.22**	268	0.12	229	**0.24**	330	0.15
X	153130911	T	G	128	0.01	127	**0.23**	99	**0.31**	124	**0.37**	117	**0.47**
6	28963272	C	T	447	0.00	341	0.00	295	0.09	305	0.00	270	0.00
11	15096326	G	C	376	0.00	470	0.05	471	0.05	492	0.00	436	0.00
11	58477469	G	C	598	0.00	519	**0.18**	540	**0.19**	347	**0.29**	414	**0.26**
13	39264986	G	C	631	0.00	866	0.07	881	0.05	765	0.00	721	0.00
13	39265203	A	T	643	0.00	951	0.06	904	0.06	842	0.00	805	0.00
13	39265204	G	C	649	0.00	951	0.06	898	0.06	844	0.00	796	0.00
16	31470915	G	T	201	0.00	331	0.06	300	0.11	394	0.12	359	0.12

16	31470916	G	T	200	0.00	325	0.06	296	0.11	397	0.12	357	0.12
17	34493506	C	T	157	0.01	246	0.11	204	0.11	361	0.05	266	0.09
19	44661602	A	G	740	0.00	546	0.03	534	0.06	530	0.00	531	0.00
19	54725745	A	G	151	0.00	230	0.05	245	0.05	310	0.04	255	0.06
19	55711745	C	G	264	0.00	311	0.04	260	0.06	341	0.00	316	0.00
20	2375239	G	A	170	0.00	241	0.03	247	0.07	191	0.00	230	0.00
1	12939854	T	G	271	0.01	386	0.05	337	0.04	303	0.04	217	0.06
1	144863355	G	A	765	0.02	960	0.11	854	0.08	789	0.14	845	0.09
1	152383294	T	G	106	0.00	130	**0.16**	147	0.06	79	**0.29**	113	0.14
1	177901894	T	C	348	0.00	399	0.09	385	0.09	437	0.00	451	0.00
1	177906566	A	T	138	0.00	201	0.15	208	0.09	157	**0.16**	182	**0.16**
1	197101460	A	G	602	0.00	818	0.00	737	0.00	720	0.00	598	0.12
1	205307648	T	G	158	0.00	231	0.05	141	0.10	182	**0.18**	142	0.13
1	216108016	A	G	566	0.00	783	0.10	714	0.08	924	0.08	665	0.11
1	233489625	T	A	303	0.00	594	0.10	534	0.08	632	0.12	512	0.11
2	55831198	A	G	501	0.00	394	0.13	364	0.10	384	0.13	330	0.12
2	240969517	G	A	140	0.01	253	0.10	233	0.07	189	0.08	176	0.07
3	17202587	G	T	324	0.00	421	0.00	403	0.00	289	0.12	420	0.00
3	122133833	C	T	783	0.00	632	0.08	540	0.11	405	**0.18**	451	**0.16**
3	124538655	T	G	505	0.00	566	0.11	524	0.13	391	**0.21**	424	**0.17**
4	9246840	C	T	58	0.00	245	0.00	198	0.02	70	0.07	126	0.05
4	23886399	G	A	640	0.00	337	0.14	308	**0.19**	191	**0.36**	226	**0.44**
5	45695892	G	A	274	0.00	314	0.12	309	0.12	224	**0.20**	270	**0.15**
5	66449402	A	C	457	0.00	294	**0.17**	282	**0.18**	204	**0.29**	268	**0.16**
5	132435344	A	G	403	0.00	195	**0.42**	209	**0.37**	152	**0.48**	148	**0.53**
5	137498986	G	A	626	0.00	629	0.00	541	0.00	486	0.02	424	0.12
5	140740440	G	T	455	0.00	326	**0.20**	314	**0.20**	253	**0.29**	282	**0.25**
6	10687346	G	A	420	0.00	504	0.05	368	0.06	273	**0.27**	322	**0.25**
6	26411799	C	A	478	0.00	284	**0.23**	306	**0.24**	272	**0.27**	192	**0.36**
6	30585669	G	A	358	0.00	343	0.13	283	**0.20**	270	**0.23**	160	**0.25**
6	37452635	C	T	304	0.00	382	0.00	323	0.00	349	0.00	248	**0.21**
6	74117273	C	A	442	0.00	277	**0.36**	285	**0.30**	226	**0.38**	192	**0.41**
6	129498957	C	T	252	0.00	200	**0.31**	205	**0.28**	101	**0.54**	127	**0.43**
6	153316401	C	T	481	0.00	459	0.15	337	**0.15**	315	**0.19**	315	0.08
7	39446285	C	A	199	0.01	521	0.00	436	0.00	494	0.06	431	0.11
7	44578717	G	A	592	0.00	940	0.08	856	0.06	1311	0.00	925	0.00
7	48317904	G	A	466	0.00	476	0.00	415	0.00	527	0.12	397	0.11
7	48431695	G	A	447	0.00	387	**0.34**	395	**0.25**	317	**0.58**	301	**0.44**
7	55240790	G	A	96	0.00	143	0.11	142	0.07	178	**0.16**	187	0.00
7	122635203	T	C	431	0.00	307	0.14	313	0.13	208	**0.27**	246	**0.24**
8	144659007	C	T	177	0.00	232	**0.15**	261	0.11	307	**0.17**	274	0.11
8	145661993	C	T	99	0.00	190	0.10	185	0.07	199	**0.25**	158	**0.25**
9	33528743	C	T	641	0.00	442	**0.36**	423	**0.35**	298	**0.50**	375	**0.53**

9	96025965	T	A	100	0.00	59	**0.17**	69	0.10	51	**0.27**	63	**0.20**
9	103004904	C	T	624	0.00	526	0.00	482	0.00	499	0.00	444	0.05
10	63170167	C	T	382	0.01	510	0.12	413	0.11	556	0.13	590	0.11
10	69648677	C	G	1034	0.00	1076	0.09	960	0.09	1192	0.11	1125	0.12
11	726625	G	A	61	0.00	74	0.00	75	0.00	62	0.00	54	0.10
11	64899782	G	T	791	0.00	742	0.13	703	**0.15**	603	**0.20**	611	**0.21**
11	66131722	C	A	220	0.00	298	0.14	277	**0.15**	276	**0.16**	254	**0.21**
11	118377152	T	G	650	0.00	447	**0.26**	516	**0.19**	249	**0.39**	323	**0.30**
11	120980024	A	C	476	0.00	321	**0.23**	298	**0.19**	186	**0.38**	196	**0.33**
12	6426740	A	C	154	0.00	208	0.00	154	0.00	133	**0.26**	170	**0.22**
12	51566200	G	T	786	0.00	324	**0.68**	418	**0.57**	78	**0.91**	179	**0.78**
12	53722599	G	T	175	0.00	173	0.07	187	0.13	157	**0.24**	140	**0.25**
12	56568506	C	T	423	0.00	496	0.00	449	0.00	272	**0.24**	321	**0.18**
12	103240709	C	G	469	0.00	450	**0.15**	434	0.13	349	**0.21**	433	**0.16**
12	120530896	C	T	242	0.00	286	0.00	290	0.00	274	0.00	277	**0.19**
13	41650316	G	A	390	0.00	383	0.00	403	0.00	201	**0.19**	197	**0.17**
13	114321950	G	A	299	0.00	211	0.00	216	0.00	152	0.06	166	0.00
14	105639361	C	T	353	0.00	299	**0.20**	275	**0.20**	239	**0.29**	332	**0.19**
16	11773243	C	T	609	0.00	1017	0.00	934	0.00	955	0.00	733	0.08
17	3716452	A	T	352	0.00	304	0.08	351	0.09	340	0.00	265	0.00
17	10450909	G	A	498	0.00	434	0.00	419	0.00	273	**0.25**	339	**0.20**
17	65337163	G	A	516	0.01	540	0.11	482	0.11	416	0.15	464	0.10
17	76113912	G	C	176	0.00	198	0.00	196	0.00	280	0.00	249	0.11
19	4317222	C	T	251	0.00	328	0.13	270	0.12	223	**0.18**	247	**0.21**
19	4510993	G	C	458	0.00	848	0.00	699	0.00	816	0.00	672	0.10
19	43998911	G	T	455	0.00	334	0.00	352	0.00	387	0.00	345	0.08
20	31040798	C	T	195	0.01	253	0.11	262	0.09	306	0.12	293	0.11
20	40052250	T	G	812	0.00	1133	0.00	999	0.00	1268	0.01	958	0.10
22	25124156	C	T	700	0.00	1041	0.09	915	0.10	1010	0.12	815	0.00
X	68382404	C	T	251	0.01	246	**0.20**	227	**0.17**	180	**0.29**	205	**0.26**

Supplementary Table S5. List of somatic mutations and allele frequencies for P8.

Ref: reference allele; Alt: alternative allele; RD: read depth; BAF: alternative allele frequency.

				Normal		**DCIS 1**		**DCIS 2**		**Primary tumor**		**Axillary lymph node metastasis**		**Asynchronous metastasis**	
**Chr**	**Start position**	**Ref**	**Alt**	**RD**	**BAF**	**RD**	**BAF**	**RD**	**BAF**	**RD**	**BAF**	**RD**	**BAF**	**RD**	**BAF**
1	25780839	C	G	483	0.00	260	**0.40**	204	**0.31**	381	**0.25**	330	0.12	217	**0.39**
1	91818691	G	C	451	0.00	271	**0.40**	211	**0.40**	325	**0.23**	360	0.09	280	0.01
1	153512658	G	A	250	0.00	171	**0.37**	114	**0.28**	143	**0.23**	202	0.12	117	**0.32**
1	155449416	C	T	196	0.00	140	**0.36**	87	**0.30**	125	**0.26**	164	0.09	76	**0.37**
1	165513913	T	C	702	0.00	1113	0.00	801	0.02	646	0.13	924	0.00	393	**0.40**
1	206773121	C	T	491	0.00	703	0.03	513	0.03	665	**0.18**	536	0.05	760	**0.26**
1	222716940	G	A	779	0.00	625	**0.40**	510	**0.36**	577	**0.28**	841	0.10	460	**0.42**
2	15615983	A	G	513	0.00	317	0.00	310	0.00	395	0.00	332	0.00	137	**0.42**
2	26696066	C	T	214	0.00	186	0.00	132	0.00	228	0.02	197	0.06	127	0.00
2	74129583	G	A	683	0.01	164	**0.62**	329	**0.20**	427	0.10	568	0.06	405	0.00
2	74762428	AG	-	201	0.00	41	**0.70**	51	**0.58**	88	**0.38**	208	0.10	29	**0.75**
2	101591350	C	T	280	0.00	50	**0.78**	101	**0.51**	149	**0.42**	235	**0.15**	42	**0.64**
2	125281951	C	A	189	0.00	159	0.01	131	0.11	142	0.00	135	0.00	94	0.00
2	133430880	T	C	309	0.00	203	0.00	146	0.00	202	0.00	250	0.00	96	**0.29**
3	15515527	G	T	494	0.00	729	0.00	461	0.05	467	0.00	576	0.00	414	0.00
3	15756165	-	G	554	0.00	470	0.06	299	**0.18**	445	**0.17**	360	0.06	178	**0.40**
3	40528870	G	A	545	0.00	681	0.00	458	0.07	510	0.00	478	0.00	436	0.00
3	57647253	G	C	580	0.00	331	**0.38**	263	**0.37**	461	**0.22**	392	0.11	294	**0.22**
3	123512526	G	A	470	0.00	478	0.00	378	0.00	441	0.10	433	0.00	209	**0.41**
3	128292524	A	G	255	0.00	250	**0.43**	186	**0.40**	268	**0.25**	335	0.12	213	**0.36**
3	167090661	G	A	1214	0.00	1011	**0.38**	778	**0.34**	937	**0.28**	1298	0.10	836	**0.38**
4	54231471	A	G	254	0.00	304	0.00	246	0.08	267	0.00	339	0.00	203	0.00
4	126238304	C	A	230	0.00	354	0.00	232	0.00	274	0.00	262	0.00	160	**0.23**
5	82834322	G	C	225	0.00	276	0.01	195	0.00	214	0.00	244	0.00	142	**0.26**
5	140784813	A	C	309	0.00	413	0.03	247	**0.18**	284	**0.19**	338	0.04	252	**0.30**
5	176868788	C	A	314	0.00	278	**0.22**	238	0.10	376	0.06	261	0.07	384	0.00
6	10687245	G	A	704	0.00	1168	0.00	835	0.00	702	0.00	942	0.00	638	**0.17**
6	157510871	G	T	227	0.00	182	0.00	135	0.00	153	0.00	232	0.00	78	**0.26**
7	57528700	G	A	530	0.00	384	**0.38**	344	**0.33**	474	**0.29**	558	0.09	212	**0.30**
7	99461188	G	T	753	0.00	610	**0.38**	456	**0.34**	442	**0.33**	709	0.08	279	**0.31**
7	135099929	G	A	520	0.00	297	**0.43**	228	**0.31**	331	**0.29**	335	0.10	167	**0.25**
7	141794570	C	G	387	0.00	490	0.00	353	0.07	400	0.00	442	0.00	239	0.00

8	43147885	G	A	480	0.00	1010	0.00	662	0.00	743	0.00	805	0.00	579	0.12
8	56436261	C	G	295	0.00	471	0.00	349	0.01	295	**0.16**	368	0.05	115	**0.41**
8	70744119	C	T	522	0.00	392	**0.31**	475	0.08	591	0.05	492	0.06	416	0.00
9	26905934	T	G	368	0.00	290	**0.33**	209	**0.34**	191	**0.34**	324	0.08	52	**0.71**
9	69423496	A	G	207	0.00	349	0.00	214	0.00	234	0.00	286	0.00	192	0.07
9	82336673	C	G	528	0.00	378	0.00	315	0.00	341	0.14	517	0.00	86	**0.63**
9	115931870	G	C	394	0.00	366	0.01	365	0.01	273	**0.19**	468	0.00	84	**0.66**
9	118949753	A	T	195	0.00	157	0.00	139	0.00	140	0.00	217	0.01	77	**0.25**
9	125562561	C	A	96	0.00	67	0.00	70	0.00	79	0.00	118	0.00	43	**0.34**
9	139992296	A	T	505	0.00	833	0.00	677	0.01	648	0.11	697	0.00	272	**0.47**
10	50038901	C	T	319	0.00	532	0.02	309	**0.27**	324	**0.17**	465	0.06	135	**0.56**
10	51768950	C	G	125	0.00	279	0.00	165	0.02	89	**0.15**	173	0.03	42	**0.58**
10	102987488	G	C	462	0.00	816	0.00	652	0.00	686	0.00	661	0.00	260	**0.31**
11	100211267	G	A	535	0.00	500	**0.41**	409	**0.35**	435	**0.28**	573	0.11	315	**0.36**
12	29936456	C	A	303	0.00	406	0.00	292	0.01	320	0.12	405	0.00	204	**0.40**
12	55524156	T	C	618	0.00	423	**0.45**	426	**0.37**	465	**0.30**	695	0.08	385	**0.39**
12	105534804	T	G	831	0.00	573	**0.40**	515	**0.33**	516	**0.31**	767	0.11	422	**0.38**
12	123481960	C	T	243	0.00	331	0.00	298	0.00	341	**0.16**	295	0.04	158	**0.41**
14	24868612	C	T	583	0.00	725	0.04	413	**0.19**	579	**0.26**	612	0.04	400	**0.24**
16	1748934	C	T	656	0.00	1301	0.00	898	0.00	1590	0.00	757	0.00	959	0.14
16	27802715	G	T	113	0.01	444	0.00	329	0.00	349	0.00	226	0.00	402	0.05
17	2203303	G	T	293	0.00	197	0.05	116	**0.38**	242	**0.31**	221	0.07	42	**0.72**
17	7577539	G	A	295	0.00	43	**0.71**	68	**0.55**	121	**0.44**	200	0.11	34	**0.73**
17	8053562	C	A	349	0.00	276	0.02	153	**0.18**	317	0.00	326	0.01	209	0.00
17	26370129	C	T	381	0.00	235	0.00	227	0.00	343	0.00	302	0.00	121	**0.26**
17	39432007	A	C	375	0.01	126	0.14	162	0.06	237	0.00	527	0.02	101	0.10
17	66267559	G	A	474	0.00	722	0.00	516	0.02	665	0.10	522	0.01	584	**0.22**
18	34298019	G	C	207	0.00	133	**0.41**	90	**0.42**	147	**0.25**	189	0.06	103	**0.35**
19	2413795	G	A	182	0.00	290	0.00	231	0.00	225	0.04	243	0.05	150	0.00
19	11347076	G	T	269	0.00	389	0.00	302	0.00	298	0.00	309	0.00	124	**0.42**
19	11728165	C	G	555	0.00	624	0.00	415	0.05	502	0.00	465	0.00	284	0.00
19	37210748	C	A	404	0.00	438	0.00	312	0.00	385	0.06	347	0.00	380	0.00
20	47887307	C	A	759	0.00	1052	0.00	790	0.00	689	0.00	935	0.00	595	**0.30**
20	62076678	C	G	440	0.01	743	0.00	498	0.01	521	0.12	535	0.00	374	**0.48**
22	23401759	G	T	202	0.00	297	0.00	244	0.02	267	0.07	252	0.00	133	0.00
22	32831768	C	T	630	0.00	536	**0.36**	375	**0.35**	370	**0.28**	539	0.11	338	0.00
X	14933827	C	G	600	0.00	381	**0.34**	358	0.09	507	0.05	438	0.04	335	0.00
X	48931607	C	A	162	0.01	195	0.00	167	0.00	234	0.00	169	0.00	179	0.14
X	76874279	C	G	654	0.00	636	0.00	440	0.07	626	0.00	524	0.00	445	0.00
X	86067999	A	G	275	0.00	264	0.00	220	0.00	188	0.00	231	0.00	92	**0.29**
X	100513435	G	A	583	0.00	800	0.03	490	**0.20**	709	**0.21**	599	0.06	206	**0.55**
X	128975839	C	T	126	0.00	93	**0.30**	57	**0.37**	83	**0.27**	112	0.08	48	**0.44**

X	149787601	GCTCACAGTCTGGTAAATTCCAGT	-	641	0.00	973	0.00	984	0.00	1007	0.04	1002	0.02	1292	0.07
15	41862464	G	A	851	0.00	1091	0.00	769	0.01	1058	0.12	950	0.00	440	**0.25**
5	140476395	T	G	53	0.00	117	0.06	85	0.08	85	0.00	90	0.05	78	0.11
19	56736057	C	T	169	0.01	371	0.03	262	0.03	312	0.01	266	0.03	596	0.05
2	120005734	G	A	173	0.01	138	0.01	125	0.00	169	0.00	159	0.00	56	**0.44**
4	22422530	T	C	661	0.00	113	**0.70**	208	**0.51**	239	**0.43**	606	0.11	130	**0.63**
5	140222517	G	A	74	0.00	82	**0.49**	83	**0.39**	91	**0.27**	116	0.15	87	**0.44**
5	140559466	T	C	154	0.01	304	0.03	261	0.07	226	0.01	250	0.07	266	0.05
6	30920123	C	T	357	0.00	321	**0.37**	205	**0.39**	243	**0.30**	338	0.08	225	**0.33**
7	150647323	G	A	197	0.00	178	**0.41**	145	**0.36**	207	**0.31**	244	0.09	86	**0.39**
8	2830685	C	T	385	0.00	88	**0.71**	113	**0.47**	196	**0.34**	408	0.11	64	**0.75**
8	24813265	G	A	133	0.00	24	**0.78**	45	**0.49**	70	**0.49**	142	**0.16**	23	**0.65**
8	70515844	A	G	584	0.00	433	**0.38**	440	**0.29**	443	**0.27**	568	0.12	228	**0.48**
8	92008947	G	T	182	0.00	299	0.01	194	0.10	220	0.14	202	0.03	159	**0.21**
9	69420331	A	G	178	0.00	217	**0.18**	165	0.12	173	0.12	226	0.02	136	**0.18**
11	1264977	C	T	266	0.00	478	0.04	226	**0.26**	244	**0.23**	344	0.07	173	**0.45**
11	86126188	C	A	473	0.00	396	0.01	258	0.10	289	0.00	397	0.00	277	0.00
12	96076553	G	A	526	0.00	341	**0.44**	282	**0.32**	311	**0.29**	504	0.10	278	**0.35**
15	82574728	T	C	626	0.00	740	0.01	576	0.00	548	0.08	624	0.00	216	**0.52**
17	39431997	C	T	371	0.01	130	0.08	159	0.01	227	0.00	494	0.02	92	0.06
17	48433589	C	T	479	0.00	808	0.00	736	0.00	785	0.00	656	0.00	1188	0.08
19	24116700	G	A	74	0.00	99	0.00	73	0.00	75	0.10	84	0.00	86	0.00
19	30935048	C	T	252	0.00	189	**0.46**	169	**0.39**	220	**0.34**	246	0.12	199	**0.36**

19	44302674	C	T	465	0.00	659	0.00	528	0.00	465	0.00	560	0.00	446	**0.21**
19	56284464	A	G	430	0.01	636	0.05	598	0.06	450	0.02	609	0.04	1123	0.06
21	33687409	G	C	213	0.00	225	0.00	154	0.00	195	0.12	217	0.00	113	**0.22**
22	46725284	A	T	486	0.00	745	0.00	526	0.00	643	0.00	561	0.00	1205	0.07
X	30739000	G	T	309	0.00	399	0.00	318	0.00	493	0.06	376	0.00	131	**0.49**

Supplementary Table S6. List of somatic mutations and allele frequencies for P11.

ALN: axillary lymph node; Ref: reference allele; Alt: alternative allele; RD: read depth; BAF: alternative allele frequency.

				Normal		Primary tumor		ALN metastasis		Asynchronous metastasis	
**Chr**	**Start position**	**Ref**	**Alt**	**RD**	**BAF**	**RD**	**BAF**	**RD**	**BAF**	**RD**	**BAF**
1	9416392	C	T	70	0.00	179	0.00	130	0.00	110	0.13
1	12855774	A	G	581	0.00	1380	0.05	1200	0.04	1118	0.03
1	12921214	C	G	676	0.00	1511	0.00	1274	0.00	1020	**0.16**
1	14108087	C	T	152	0.00	241	0.00	157	0.00	184	0.14
1	16578007	T	C	93	0.00	116	**0.44**	145	0.09	127	**0.22**
1	22927921	C	T	88	0.00	135	**0.36**	198	0.05	124	**0.33**
1	55167779	G	A	245	0.00	528	0.14	601	0.00	561	0.00
1	65871793	G	A	336	0.00	602	**0.17**	594	0.07	582	0.00
1	74957838	G	A	283	0.01	444	0.06	446	0.07	443	0.00
1	79095577	G	C	357	0.01	681	0.00	575	0.00	519	0.07
1	146766150	G	A	550	0.00	871	0.10	947	0.00	1158	0.00
1	151131580	C	T	362	0.00	434	**0.43**	655	0.09	541	**0.30**
1	155005648	C	T	426	0.00	929	0.06	779	0.11	977	0.00
1	157488547	C	A	281	0.01	365	0.09	440	0.00	470	0.00
1	161017939	G	A	70	0.00	227	0.04	145	0.07	184	0.00
1	208061249	G	A	66	0.00	139	0.09	103	0.00	158	0.00
1	214537889	G	A	206	0.00	186	**0.41**	314	0.06	252	**0.36**
1	217793431	C	T	444	0.00	951	0.00	831	0.00	956	0.12
1	226180662	T	G	336	0.00	373	**0.41**	492	0.09	528	**0.22**
1	233807148	G	C	222	0.00	415	0.00	315	0.07	426	0.00
1	248402975	G	A	260	0.00	487	0.00	421	0.00	450	0.08
2	7027068	G	A	422	0.00	717	0.00	686	0.05	624	0.00
2	21231975	C	T	481	0.00	940	0.05	919	0.06	1096	0.00
2	37268311	C	G	419	0.00	752	0.00	551	0.00	494	0.12
2	63272627	C	T	360	0.00	555	0.00	445	0.00	419	0.13
2	68772386	G	A	355	0.00	481	**0.16**	541	0.07	677	0.00
2	80101241	G	A	117	0.00	234	0.00	189	0.00	184	0.13
2	86327278	G	T	317	0.00	335	**0.40**	481	0.08	437	**0.25**
2	103141525	G	A	465	0.00	768	0.09	737	0.00	801	0.00
2	107039712	C	T	300	0.01	757	0.05	618	0.04	764	0.05
2	118582249	G	A	354	0.01	465	0.10	505	0.00	711	0.00
2	122005784	C	T	132	0.00	220	0.11	223	0.00	215	0.00
2	136594315	C	T	423	0.00	609	**0.28**	788	0.00	494	**0.34**

2	163000611	C	G	308	0.00	477	0.11	431	0.00	402	0.00
2	168114757	G	A	620	0.00	1000	0.00	891	0.00	652	**0.16**
2	168114936	G	A	412	0.00	651	0.00	462	0.00	387	0.14
2	168115358	G	C	374	0.00	622	0.00	545	0.00	378	**0.15**
2	170780478	C	T	765	0.00	1158	0.00	1135	0.00	877	**0.15**
2	198266834	T	C	493	0.00	435	**0.42**	574	0.08	446	**0.33**
2	201873785	C	G	575	0.00	909	0.06	767	0.10	883	0.00
2	204039992	TCCAACTTCAGAGTG	-	212	0.00	237	**0.31**	271	0.04	300	0.13
2	204591542	C	G	533	0.00	875	0.06	755	0.08	856	0.00
2	242545862	C	G	225	0.00	393	0.09	421	0.00	437	0.00
3	4878533	G	A	424	0.00	481	0.00	410	0.00	410	0.13
3	12854853	G	C	282	0.00	543	0.00	402	0.00	395	0.08
3	44674066	G	C	365	0.00	341	0.00	525	0.00	411	**0.18**
3	50214225	G	C	327	0.00	453	0.00	631	0.00	398	**0.21**
3	111793232	C	G	482	0.00	581	0.00	584	0.00	555	0.13
3	178952085	A	G	614	0.00	472	**0.75**	791	**0.19**	651	**0.59**
3	193062840	C	A	251	0.00	891	0.06	429	0.00	681	0.00
3	195507196	G	C	751	0.00	1959	0.08	1760	**0.16**	2409	0.00
3	195507218	G	C	787	0.00	2019	0.08	1847	**0.15**	2492	0.00
3	195507484	G	C	257	0.00	1025	0.08	954	0.14	1295	0.00
3	195507698	G	C	369	0.00	833	0.07	783	0.13	1026	0.00
3	195508924	G	T	237	0.00	959	0.07	746	**0.16**	1050	0.00
4	46043205	C	G	368	0.00	433	0.00	534	0.00	274	**0.18**
4	57189672	C	T	201	0.00	200	0.00	317	0.00	145	**0.22**
4	71507891	G	A	519	0.00	725	0.08	638	0.06	572	0.00
4	87683914	G	T	376	0.00	538	0.00	619	0.00	380	**0.21**
4	99397431	T	C	326	0.00	598	0.00	589	0.00	397	**0.17**
4	113507612	G	C	442	0.00	499	**0.22**	586	0.00	455	0.14
4	123329098	G	C	523	0.00	760	0.00	794	0.00	652	**0.17**
4	151773611	G	A	386	0.00	560	0.00	636	0.00	516	**0.19**
4	162306909	C	T	450	0.00	286	**0.48**	655	0.07	633	0.14
5	55237115	T	A	482	0.00	444	**0.42**	884	0.08	706	**0.28**
5	55250653	G	A	543	0.00	745	0.15	763	0.00	848	0.00
5	71492532	C	G	332	0.00	376	**0.41**	465	0.09	444	**0.31**
5	79028961	C	G	330	0.00	486	0.07	481	0.00	473	0.00
5	140083545	C	G	556	0.00	763	0.00	678	0.00	652	0.15
5	140216151	C	G	249	0.00	490	0.00	477	0.00	378	**0.16**
5	140229446	G	A	181	0.00	378	0.00	440	0.00	367	**0.15**

5	140256909	G	A	181	0.00	373	0.00	385	0.00	303	**0.21**
5	140562571	G	T	439	0.00	579	**0.30**	856	0.00	802	**0.15**
5	140562661	C	T	511	0.00	625	**0.29**	937	0.00	817	0.14
5	147040771	C	A	526	0.00	995	0.00	1020	0.00	769	**0.18**
5	173317553	C	T	344	0.00	605	0.05	392	0.08	385	0.00
5	178044362	T	A	504	0.00	505	**0.42**	795	0.07	517	**0.35**
6	70049340	T	A	318	0.00	476	0.10	505	0.00	554	0.00
6	97423877	C	T	279	0.00	259	**0.40**	391	0.09	365	**0.26**
6	117114236	C	A	429	0.00	646	0.00	632	0.00	632	0.14
6	117665339	G	T	433	0.00	591	0.12	603	0.00	671	0.00
6	124604274	C	T	857	0.00	1561	0.09	1692	0.00	2036	0.00
6	126278203	C	T	220	0.00	254	**0.43**	397	0.05	304	**0.29**
6	144093466	C	T	188	0.00	216	**0.30**	285	0.00	243	**0.18**
7	4107484	G	A	255	0.00	408	0.00	435	0.00	295	**0.19**
7	27169038	G	A	176	0.00	154	**0.37**	253	0.06	159	**0.35**
7	39504038	G	A	266	0.00	405	0.15	469	0.00	280	**0.34**
7	50595917	G	A	218	0.01	300	0.05	281	0.06	284	0.00
7	119915658	G	C	379	0.00	636	0.00	557	0.00	366	**0.23**
7	130417935	G	A	97	0.00	138	**0.27**	156	0.07	100	**0.22**
7	138344684	G	A	288	0.00	410	0.09	471	0.00	355	0.00
8	25323826	G	A	322	0.00	184	**0.19**	325	0.00	179	0.00
8	68062089	A	-	605	0.00	1108	0.00	733	0.00	599	**0.16**
8	86121594	C	G	267	0.00	490	0.04	290	0.07	343	0.00
8	103297469	G	C	419	0.00	782	0.00	616	0.00	515	0.11
8	110408352	G	A	248	0.00	349	0.00	221	0.00	232	0.09
9	15453071	G	A	556	0.00	872	0.10	1112	0.00	1108	0.00
9	91159414	C	A	370	0.00	108	**0.82**	615	0.10	386	**0.44**
9	123931922	T	C	306	0.00	296	0.00	370	0.07	402	0.00
10	17746511	C	G	314	0.00	330	**0.39**	430	0.08	410	**0.27**
10	33552577	C	T	165	0.00	191	**0.40**	258	0.05	203	**0.30**
10	73461953	G	A	327	0.01	739	0.00	696	0.00	631	0.14
10	75549732	C	T	212	0.00	438	0.07	410	0.00	418	0.00
10	79603434	G	C	356	0.00	689	**0.17**	708	0.08	884	0.00
10	102058318	C	T	214	0.00	247	**0.40**	357	0.07	335	**0.18**
10	115338409	G	A	330	0.00	628	0.13	583	0.00	625	0.00
11	10555623	C	T	690	0.00	817	0.10	775	0.00	761	0.00
11	12263906	C	T	322	0.00	340	**0.46**	607	0.06	381	**0.34**
11	65402457	C	T	460	0.00	654	**0.44**	948	0.07	740	**0.29**
11	93430735	C	T	430	0.00	84	**0.80**	574	0.10	349	**0.37**
11	93431235	C	G	531	0.00	121	**0.75**	743	0.08	488	**0.35**
11	103059317	G	T	515	0.00	69	**0.79**	565	0.08	269	**0.38**
11	123480940	G	A	271	0.01	400	0.00	516	0.00	300	**0.18**
12	6172182	G	A	211	0.00	503	0.02	398	0.08	495	0.00

12	39701482	G	A	437	0.01	775	0.05	630	0.08	763	0.00
12	48073341	G	C	205	0.00	259	0.03	183	0.05	287	0.00
12	53552324	C	G	174	0.00	327	0.00	295	0.00	267	0.11
12	72017347	C	G	264	0.00	389	0.00	261	0.08	346	0.00
12	108634180	G	A	325	0.01	426	**0.41**	593	0.07	505	**0.32**
12	109972453	G	C	223	0.00	463	0.00	415	0.00	394	0.15
12	111926308	C	T	450	0.00	703	0.11	659	0.00	721	0.00
12	118590088	C	G	203	0.00	376	0.07	313	0.09	405	0.00
13	28127448	C	T	258	0.00	256	0.06	384	0.00	346	0.00
13	103419763	C	A	271	0.00	266	0.00	339	0.00	221	**0.22**
14	65220495	G	T	257	0.00	224	**0.53**	475	0.10	322	**0.33**
14	67807157	C	G	644	0.00	837	0.11	961	0.00	958	0.00
15	41071787	G	A	147	0.00	233	0.09	157	0.01	174	0.00
15	41688993	C	G	322	0.00	495	0.08	501	0.00	434	0.00
15	62939594	G	C	356	0.01	668	**0.16**	668	0.06	810	0.00
15	91304097	G	C	289	0.00	515	0.08	368	0.00	380	0.01
16	1278703	G	T	145	0.00	452	0.00	374	0.00	451	0.13
16	84199538	G	C	360	0.00	256	0.10	369	0.09	338	0.00
16	88502012	G	C	50	0.00	56	**0.22**	109	0.00	106	0.00
16	88502288	G	A	143	0.00	151	**0.18**	264	0.00	238	0.00
17	7578203	C	T	437	0.00	121	**0.79**	644	0.09	376	**0.37**
17	13399603	C	T	167	0.01	229	0.00	288	0.00	201	**0.20**
17	27014467	G	A	141	0.01	263	**0.25**	347	0.00	213	**0.30**
17	33510574	G	C	152	0.00	230	0.09	216	0.00	280	0.00
17	38953457	G	A	286	0.00	485	**0.20**	462	0.11	581	0.00
17	73808362	G	A	106	0.00	245	0.00	216	0.00	198	0.15
17	74395040	G	A	243	0.00	605	0.00	552	0.00	510	0.14
18	10742567	C	A	548	0.00	486	0.14	937	0.00	893	0.00
18	71930652	C	G	378	0.00	923	0.04	587	0.08	547	0.00
19	9073848	G	A	231	0.00	413	0.07	411	0.00	477	0.00
19	10418951	C	T	162	0.00	305	**0.42**	416	0.06	369	**0.27**
19	17370132	C	T	190	0.00	485	0.00	417	0.00	410	0.12
19	17370231	C	G	238	0.00	590	0.00	489	0.00	495	0.12
19	17370816	C	G	123	0.01	304	0.00	240	0.00	220	0.10
19	22157223	C	T	374	0.00	416	0.00	419	0.11	501	0.00
19	37100839	C	T	370	0.00	355	**0.38**	532	0.07	382	**0.41**
19	45728161	C	T	229	0.00	336	0.11	357	0.00	468	0.00
19	49639166	G	A	66	0.00	143	0.00	114	0.00	107	0.11
19	55105939	C	T	192	0.00	376	0.00	321	0.00	284	0.14
19	56175087	A	G	418	0.00	928	0.00	748	0.00	795	0.12
19	56952557	T	A	423	0.00	726	0.00	740	0.00	713	0.13
20	3128912	G	C	299	0.00	479	0.00	389	0.00	368	0.15
20	34025432	C	T	121	0.00	170	**0.35**	210	0.06	213	**0.23**

20	48130881	G	A	355	0.00	759	0.00	775	0.00	820	0.13
20	57429238	A	T	295	0.00	738	0.11	750	0.00	764	0.00
20	58330242	G	A	418	0.00	472	**0.41**	678	0.06	598	**0.27**
21	34929479	G	A	185	0.00	137	**0.54**	241	0.07	184	**0.21**
21	44485526	C	T	291	0.01	320	**0.53**	530	0.07	480	**0.17**
22	20104408	C	T	119	0.00	302	0.13	246	0.00	229	0.00
22	21066893	C	T	84	0.00	209	0.00	195	0.00	189	0.14
22	29445460	C	G	77	0.00	172	0.02	116	0.13	143	0.00
22	36116702	G	A	262	0.01	489	0.00	462	0.00	402	**0.16**
22	47086067	C	T	226	0.00	454	0.14	431	0.00	416	0.00
X	102337762	C	T	327	0.00	261	**0.23**	426	0.00	418	0.00
X	153640464	T	A	308	0.00	417	**0.24**	876	0.00	706	0.00
1	152282857	C	T	230	0.00	636	0.00	469	0.00	482	0.14
3	195513680	A	T	190	0.00	717	0.07	747	0.03	846	0.04
5	140516858	C	G	120	0.00	223	0.10	243	0.00	276	0.00
7	82586170	A	G	382	0.00	478	0.09	563	0.00	484	0.00
9	37493637	G	A	389	0.00	533	0.00	528	0.00	454	0.14
10	104596925	G	A	162	0.00	415	0.09	390	0.00	366	0.00
11	6898196	C	G	253	0.00	363	0.06	416	0.00	412	0.00
12	12814733	G	T	314	0.00	557	0.06	513	0.00	618	0.00
12	31243026	C	A	78	0.00	157	0.05	151	0.01	138	0.01
15	51791529	G	T	491	0.00	636	0.06	669	0.07	857	0.00
16	31139437	G	A	393	0.00	887	0.13	922	0.00	1021	0.00
18	13734485	C	T	321	0.00	2156	0.06	521	0.00	472	0.00
1	12855644	A	G	308	0.00	953	0.06	860	0.03	798	0.04
1	17264148	G	T	248	0.00	522	0.11	478	0.00	479	0.00
1	21133953	C	T	83	0.00	147	0.00	102	0.00	61	**0.21**
1	23885621	G	A	225	0.00	538	0.00	490	0.00	339	**0.17**
1	24409183	C	T	249	0.00	603	0.00	442	0.00	356	**0.17**
1	26386768	G	T	195	0.00	231	**0.37**	344	0.05	207	**0.29**
1	38338809	G	C	307	0.01	589	0.07	606	0.00	513	0.00
1	53542907	G	T	483	0.01	703	**0.45**	1121	0.09	955	**0.19**
1	64059204	G	A	350	0.00	487	**0.39**	705	0.07	413	**0.30**
1	145295460	G	A	771	0.00	1394	0.09	1371	0.00	1446	0.00
1	150414424	G	A	345	0.00	503	0.09	341	0.00	401	0.00
1	160267149	C	T	485	0.00	738	0.03	596	0.08	746	0.00
2	27706234	C	A	375	0.01	564	0.00	384	0.00	363	0.12
2	65540968	C	T	174	0.00	351	0.00	313	0.00	330	0.15
2	74641552	G	A	115	0.00	219	0.08	182	0.06	263	0.00
2	85820199	C	T	331	0.00	836	0.04	582	0.10	774	0.00
2	113333075	C	T	425	0.00	714	0.00	678	0.00	776	0.11
2	242192385	C	T	119	0.00	295	0.00	221	0.06	227	0.00
3	46307387	G	A	291	0.00	253	0.00	391	0.00	288	**0.17**

4	66467417	C	A	92	0.00	123	0.00	158	0.00	129	0.07
4	153562088	G	A	281	0.00	202	**0.47**	341	0.12	287	0.15
5	6620341	C	T	125	0.01	163	0.00	159	0.00	162	**0.16**
5	140306933	C	T	185	0.00	216	**0.29**	319	0.00	232	**0.19**
6	108582093	C	G	166	0.00	296	0.12	326	0.00	328	0.00
7	43477679	C	A	362	0.01	636	0.00	639	0.00	515	**0.18**
7	43916353	C	T	261	0.00	425	0.11	450	0.00	441	0.00
7	93090184	G	T	356	0.00	468	0.05	560	0.00	581	0.00
7	111487112	C	T	573	0.00	401	**0.47**	853	0.07	685	**0.19**
8	23563980	C	G	141	0.00	172	0.08	339	0.00	144	0.00
9	32542006	C	T	545	0.00	1026	0.08	795	0.00	1027	0.00
9	131360684	G	C	355	0.00	332	0.10	447	0.06	408	0.00
9	140007546	G	A	400	0.01	94	**0.81**	653	0.10	330	**0.40**
10	43088106	G	C	436	0.00	644	0.00	597	0.00	591	0.13
10	81373501	C	T	138	0.00	227	0.00	203	0.00	201	**0.17**
11	6646447	G	A	117	0.00	184	0.00	147	0.07	158	0.00
11	55861458	G	T	268	0.00	287	**0.27**	346	0.01	269	**0.33**
11	124180285	G	T	228	0.00	287	0.10	257	0.10	210	0.00
12	48440187	G	A	402	0.00	567	0.10	537	0.00	573	0.00
12	131466598	C	T	190	0.00	416	0.08	343	0.00	449	0.00
13	49039214	G	C	171	0.00	116	0.06	199	0.00	193	0.01
14	24546188	G	A	79	0.00	123	**0.25**	125	0.05	124	0.00
14	56645167	G	A	178	0.01	274	0.00	270	0.00	218	0.14
14	74086147	C	T	440	0.00	609	0.14	754	0.00	754	0.00
14	90651056	C	T	393	0.00	665	0.13	964	0.00	912	0.00
15	23811550	T	A	165	0.00	426	0.00	362	0.00	333	**0.18**
15	35045438	G	A	174	0.00	421	0.00	360	0.00	282	**0.18**
15	52486210	C	T	309	0.00	583	0.11	572	0.00	615	0.00
16	31539490	C	T	293	0.00	338	**0.41**	465	0.10	453	**0.27**
17	8051519	G	A	400	0.00	472	0.06	735	0.00	679	0.00
17	55183170	A	G	409	0.00	634	**0.15**	547	0.09	677	0.00
17	59853816	T	C	531	0.00	770	0.11	733	0.00	887	0.00
19	35512451	G	A	200	0.00	397	0.00	379	0.00	349	0.14
19	40704352	C	T	300	0.00	314	**0.48**	418	0.09	430	**0.17**
19	45821086	G	C	385	0.00	1001	0.00	845	0.00	706	0.13
19	56244036	C	T	325	0.00	892	0.05	626	0.09	758	0.00
20	4162792	C	T	116	0.00	290	0.05	255	0.10	300	0.00
20	30457386	G	C	359	0.00	1019	0.00	1025	0.00	1106	0.11
20	62192819	C	T	153	0.00	248	**0.45**	345	0.08	278	**0.33**
21	38117319	C	T	414	0.00	686	**0.32**	828	0.09	611	**0.31**
22	29445390	C	G	94	0.00	199	0.05	135	0.09	190	0.00
22	35734736	C	T	100	0.01	244	0.00	207	0.00	177	**0.17**
22	37492072	G	A	341	0.00	471	**0.40**	613	0.10	500	**0.29**

22	39381990	G	A	357	0.00	1022	0.00	858	0.09	1003	0.00
X	43590619	G	C	371	0.00	395	0.11	521	0.00	490	0.00
X	46434206	G	A	189	0.00	286	0.00	377	0.00	274	**0.20**

Supplementary Table S7. List of somatic mutations and allele frequencies for P15.

Ref: reference allele; Alt: alternative allele; RD: read depth; BAF: alternative allele frequency.

				Normal		Primary tumor				Asynchronous metastasis	
**Chr**	**Start position**	**Ref**	**Alt**	**RD**	**BAF**	**RD**	**AD**	**DP**	**BAF**	**RD**	**BAF**
1	46978038	C	T	171	0.00	85	207	292	**0.71**	382	0.06
1	228336153	C	G	83	0.00	146	87	233	**0.37**	189	0.02
2	128262275	C	T	311	0.00	868	50	918	0.05	944	0.00
4	187628104	C	A	171	0.00	332	38	370	0.10	329	0.00
5	169673042	AGAA	-	357	0.00	778	153	931	**0.16**	773	0.00
6	4099327	C	A	283	0.00	406	194	600	**0.32**	389	0.03
6	74228891	-	G	231	0.00	369	228	597	**0.38**	521	0.06
6	123101438	G	A	272	0.00	454	54	508	0.11	509	0.00
7	21934326	G	T	361	0.00	446	194	640	**0.30**	581	0.05
8	10555330	G	A	134	0.00	110	68	178	**0.38**	262	0.04
9	137017107	G	A	143	0.00	204	65	269	**0.24**	291	0.00
10	75526139	G	A	186	0.00	211	108	319	**0.34**	266	0.04
11	111228436	-	T	132	0.01	127	66	193	**0.34**	274	0.06
11	118235870	CAAA	-	95	0.00	60	26	86	**0.30**	129	0.00
12	6182868	A	G	100	0.00	167	95	262	**0.36**	237	0.06
12	58217489	C	T	138	0.01	219	145	364	**0.40**	303	0.06
15	45270692	A	G	166	0.00	182	0	182	0.00	123	0.08
15	59175976	C	G	168	0.00	228	91	319	**0.29**	286	0.07
16	31425892	G	T	181	0.00	435	80	515	**0.16**	280	0.00
17	7979527	C	T	84	0.00	86	71	157	**0.45**	123	0.10
17	61972843	G	A	132	0.00	185	117	302	**0.39**	290	0.07
18	7043334	C	G	414	0.00	546	407	953	**0.43**	841	0.05
18	22806228	G	T	261	0.00	386	249	635	**0.39**	574	0.06
18	50278483	A	G	261	0.00	493	0	493	0.00	361	0.05
20	44672550	G	A	149	0.01	419	1044	1463	**0.71**	310	**0.31**
20	57611558	TCAGTCCT	-	158	0.00	1610	99	1709	0.06	471	0.03
22	42910779	A	T	204	0.00	413	258	671	**0.38**	603	0.05
X	103294850	C	G	65	0.00	80	26	106	**0.25**	96	0.00
X	105189978	G	T	335	0.00	477	135	612	**0.22**	550	0.00
7	137773384	C	A	206	0.00	342	20	362	0.06	345	0.00
15	80743272	C	T	269	0.01	652	52	704	0.07	732	0.00
2	189868848	G	A	87	0.00	91	25	116	**0.22**	114	0.00
3	9979774	C	T	140	0.01	151	106	257	**0.41**	206	0.05
4	110865202	C	G	232	0.00	250	135	385	**0.35**	309	0.03

5	9190575	G	A	377	0.00	525	273	798	**0.34**	724	0.03
5	140573991	G	C	57	0.00	194	18	212	0.08	142	0.06
7	151945313	A	G	465	0.01	2778	180	2958	0.06	2882	0.05
9	43626815	G	A	93	0.01	255	25	280	0.09	191	0.07
10	93744	T	C	531	0.01	3144	173	3317	0.05	2770	0.03
10	5014488	C	T	67	0.00	252	24	276	0.09	198	0.03
10	51465097	G	A	63	0.00	735	61	796	0.08	577	0.09
11	68682452	G	A	176	0.00	230	146	376	**0.39**	335	0.04
21	46011298	G	A	100	0.02	170	14	184	0.08	153	0.07
X	3228936	G	A	176	0.00	245	156	401	**0.39**	314	0.06

Supplementary Table S8. List of somatic mutations and allele frequencies for P46.

ALN: axillary lymph node; Ref: reference allele; Alt: alternative allele; RD: read depth; BAF: alternative allele frequency.

	P 46			Normal		DCIS		Primary tumor		ALN metastasis	
Chr	Start position	Ref	Alt	RD	**BAF**	RD	**BAF**	RD	**BAF**	RD	**BAF**
1	6150470	G	C	351	0.00	500	0.04	393	0.15	424	**0.24**
1	12516111	G	C	431	0.00	524	0.11	536	0.00	530	0.00
1	24432473	C	T	169	0.00	137	**0.23**	128	**0.16**	117	**0.22**
1	46049852	G	A	238	0.00	100	0.08	113	**0.20**	73	**0.40**
1	53990545	G	A	350	0.00	379	**0.23**	326	**0.25**	258	**0.36**
1	59148013	G	C	415	0.00	409	0.04	367	**0.18**	200	**0.39**
1	74671116	G	A	360	0.00	271	**0.23**	283	**0.23**	146	**0.38**
1	90482919	G	-	852	0.00	724	**0.20**	801	**0.23**	494	**0.34**
1	145296448	T	A	473	0.00	1864	0.05	1844	0.07	1632	0.05
1	150667263	C	T	516	0.00	439	0.13	588	0.01	472	0.00
1	152285414	G	C	209	0.00	834	**0.19**	807	**0.18**	574	**0.24**
1	152325342	C	G	125	0.00	235	**0.19**	212	**0.19**	148	**0.29**
1	155034383	G	T	281	0.00	471	0.05	429	**0.16**	326	**0.25**
1	156280855	C	G	368	0.00	586	0.15	685	0.01	635	0.00
1	158450340	C	T	493	0.00	639	0.03	549	0.14	380	**0.24**
1	161496182	A	T	287	0.00	391	0.03	305	**0.17**	255	**0.30**
1	173915664	G	A	403	0.01	350	**0.19**	454	0.15	317	**0.25**
1	184764838	C	G	397	0.00	913	0.02	661	0.14	511	**0.25**
1	186283131	C	T	483	0.00	297	0.01	321	0.09	233	**0.18**
1	197169035	G	A	469	0.00	361	0.05	346	0.14	262	**0.27**
1	205241220	T	C	262	0.00	409	0.11	507	0.00	413	0.00
1	220145434	G	A	445	0.00	567	0.08	692	0.00	637	0.00
1	236751250	C	G	645	0.00	513	**0.17**	627	**0.16**	486	**0.25**
2	23977614	A	G	319	0.00	162	**0.22**	217	**0.18**	159	**0.25**
2	29297015	C	A	227	0.00	356	**0.15**	313	0.14	273	**0.20**
2	29416462	G	T	233	0.00	184	**0.23**	216	0.15	166	**0.23**
2	44565513	C	A	629	0.00	589	0.02	547	0.15	411	**0.29**
2	71337101	C	G	406	0.00	309	0.04	270	**0.17**	217	**0.26**
2	71371600	C	T	617	0.00	750	0.04	697	**0.15**	531	**0.26**
2	88387524	C	T	676	0.00	781	0.03	570	**0.17**	488	**0.29**

2	97270109	G	C	157	0.00	200	**0.19**	204	**0.23**	150	**0.25**
2	98164186	C	T	430	0.00	219	**0.17**	266	**0.18**	188	**0.20**
2	112638389	T	C	515	0.00	495	**0.18**	543	**0.17**	394	**0.27**
2	114257443	A	C	125	0.00	304	0.06	275	0.04	256	0.03
2	135711268	G	C	523	0.00	506	0.04	491	**0.18**	391	**0.19**
2	152289706	G	T	413	0.00	377	0.05	405	**0.19**	247	**0.29**
2	152374876	G	C	462	0.00	670	0.05	619	**0.17**	427	**0.26**
2	157406158	C	G	227	0.00	392	0.03	365	0.14	262	**0.24**
2	158275044	G	C	782	0.00	425	**0.18**	414	**0.17**	336	**0.24**
2	166183453	G	T	439	0.00	707	0.10	586	0.01	576	0.00
2	170493353	G	C	629	0.00	489	0.11	680	0.01	476	0.00
2	179510706	G	-	377	0.00	355	0.13	434	0.02	350	0.00
2	179548791	C	G	255	0.00	247	0.02	216	**0.18**	156	**0.24**
2	182542737	G	C	279	0.00	408	0.06	341	0.00	308	0.00
2	202552076	C	T	116	0.00	79	**0.29**	109	0.03	89	0.00
2	210856936	C	A	265	0.00	443	0.02	313	0.10	290	**0.21**
2	234243710	C	G	229	0.00	293	0.07	257	0.00	210	0.00
2	234621681	G	T	289	0.00	401	0.02	271	0.11	204	**0.24**
2	234669166	C	T	482	0.00	599	**0.49**	596	**0.35**	357	**0.55**
2	240969158	G	T	457	0.00	450	0.08	591	0.00	499	0.00
3	33638227	C	G	297	0.00	167	0.07	218	0.00	181	0.00
3	53839140	C	G	199	0.00	368	0.15	368	0.01	261	0.00
3	64599049	G	A	365	0.00	338	0.06	271	0.00	247	0.00
3	77626741	G	C	289	0.00	337	0.03	312	**0.18**	216	**0.28**
3	113684043	C	G	393	0.00	498	0.03	514	0.11	450	**0.15**
3	126202249	C	G	463	0.00	1114	0.04	790	**0.22**	770	**0.28**
3	132389843	C	G	715	0.00	656	0.10	959	0.00	870	0.00
3	151163758	C	G	515	0.00	1186	0.02	1152	0.12	1063	0.14
3	169540551	C	G	538	0.00	318	0.14	273	**0.23**	276	**0.30**
3	172674584	C	T	411	0.00	575	**0.15**	759	0.10	591	**0.19**
3	178916924	CAGTAG	-	967	0.00	530	**0.42**	623	**0.46**	390	**0.65**
3	179291217	C	G	687	0.00	715	0.02	823	0.11	755	**0.16**
3	195780321	C	T	532	0.00	1099	0.06	938	**0.20**	834	**0.33**
3	195791270	C	G	149	0.00	134	0.04	145	**0.22**	90	**0.33**
4	7043264	C	T	494	0.00	372	0.06	368	**0.18**	226	**0.33**
4	48165756	C	T	658	0.00	595	0.07	706	0.00	383	0.00
4	106317482	G	C	459	0.00	255	0.06	281	**0.23**	117	**0.42**
4	109822324	T	C	194	0.00	118	**0.22**	122	**0.23**	62	**0.33**
4	129782960	C	G	296	0.00	465	0.03	352	**0.19**	236	**0.36**
4	130027752	-	T	810	0.00	640	**0.31**	780	**0.24**	511	**0.32**
4	151505032	-	ACACCC	1170	0.00	699	0.07	697	**0.21**	450	**0.36**
5	26881664	C	T	540	0.00	1042	**0.15**	888	**0.32**	790	**0.37**
5	33455774	G	T	534	0.00	565	0.13	567	0.14	651	**0.15**

5	40981620	G	C	329	0.00	792	0.12	698	0.13	673	0.15
5	44811182	C	G	604	0.00	573	0.03	566	0.14	511	**0.17**
5	52954410	C	T	445	0.00	566	0.11	641	0.12	589	**0.25**
5	79732913	G	T	465	0.00	689	0.06	720	0.00	713	0.00
5	130840440	C	T	417	0.00	292	**0.19**	253	**0.19**	144	**0.34**
5	140710657	G	C	335	0.00	411	0.03	311	**0.20**	223	**0.30**
5	142435604	G	A	626	0.00	582	**0.33**	666	**0.23**	451	**0.35**
5	150945353	G	C	377	0.00	682	0.02	391	**0.19**	276	**0.35**
5	168671716	C	G	267	0.00	109	**0.36**	158	**0.16**	79	**0.41**
6	12122261	G	C	316	0.00	382	**0.16**	394	0.13	314	**0.22**
6	12294615	G	C	473	0.00	694	0.01	858	0.09	560	**0.18**
6	17637542	G	C	350	0.00	339	0.13	370	0.13	313	**0.18**
6	26156937	C	A	140	0.00	74	0.09	79	0.14	93	**0.15**
6	27783109	G	C	533	0.00	423	0.03	415	0.12	287	**0.22**
6	30653372	C	G	317	0.00	713	0.04	570	**0.15**	606	**0.17**
6	32017844	A	G	451	0.00	467	**0.17**	431	0.13	407	**0.23**
6	32790075	C	T	869	0.00	749	0.08	1022	0.00	913	0.00
6	32945918	G	T	210	0.00	159	0.12	228	0.14	163	**0.16**
6	33245680	G	A	264	0.00	322	0.14	250	**0.20**	308	**0.18**
6	33288573	G	A	530	0.00	518	**0.15**	488	0.15	408	**0.23**
6	36943459	C	G	331	0.00	547	0.02	510	0.10	414	**0.19**
6	42796307	C	G	483	0.00	831	0.02	713	**0.17**	587	**0.23**
6	49425547	C	T	355	0.00	394	0.13	434	0.14	381	**0.20**
6	53379122	C	G	401	0.00	418	0.04	457	0.10	403	**0.19**
6	56437730	G	A	434	0.00	509	0.13	566	0.13	469	**0.23**
6	70071207	G	A	634	0.00	875	**0.16**	937	**0.16**	782	**0.20**
6	70990717	G	T	152	0.00	296	0.12	285	0.01	352	0.00
6	73751765	G	C	523	0.01	673	0.14	818	**0.15**	593	**0.22**
6	80746257	G	C	451	0.00	480	0.11	586	0.01	581	0.00
6	84930880	C	T	659	0.00	676	0.09	769	0.13	697	**0.19**
6	87725534	G	A	431	0.00	629	0.03	607	0.14	536	**0.19**
6	90453342	C	G	376	0.01	489	0.03	464	0.14	451	**0.19**
6	116720684	C	G	347	0.00	428	0.02	424	0.10	400	0.15
6	128302332	C	G	361	0.00	617	0.03	594	0.10	535	**0.18**
6	128505754	C	G	374	0.00	599	0.10	636	**0.15**	516	**0.17**
6	133844306	A	G	448	0.00	963	0.02	834	0.09	984	0.10
6	135787091	T	C	378	0.00	526	0.11	630	0.09	568	0.11
6	136597038	G	A	525	0.00	742	0.03	717	0.11	633	**0.17**
6	136710508	C	T	231	0.00	264	0.03	271	0.13	257	0.13
6	136990456	T	C	532	0.00	904	0.06	989	0.10	1013	0.10
6	139159581	C	A	329	0.00	669	0.02	798	0.08	706	0.13
6	139487726	C	G	286	0.00	608	0.01	504	0.10	572	0.09
6	139568989	C	G	612	0.00	1146	0.09	1282	0.08	1278	0.11

6	143074552	G	C	766	0.00	868	0.09	973	0.08	917	0.11
6	146239311	G	A	569	0.00	713	0.02	1012	0.07	941	0.12
6	146993435	G	A	287	0.00	517	0.02	710	0.07	702	0.11
6	152554975	C	G	391	0.00	626	0.11	742	0.09	735	0.09
6	155578001	G	T	689	0.00	1474	0.00	1587	0.01	1740	0.07
6	155578045	C	A	604	0.00	1437	0.00	1644	0.01	1737	0.06
6	155751994	C	T	435	0.00	2151	0.01	2470	0.05	2851	0.07
6	161527605	G	C	517	0.00	1492	0.07	2091	0.06	2008	0.07
7	6182608	G	A	387	0.00	582	0.03	628	0.14	390	**0.24**
7	6561128	G	C	332	0.00	449	0.11	474	0.00	427	0.00
7	11676470	C	G	384	0.00	440	0.08	507	0.00	365	0.00
7	21945150	G	C	432	0.00	681	0.01	439	0.13	426	**0.22**
7	34724246	G	C	241	0.00	237	0.15	242	0.13	190	**0.23**
7	91632268	G	A	531	0.00	416	**0.20**	449	**0.21**	297	**0.37**
7	91690613	G	A	637	0.00	532	0.14	857	0.01	515	0.00
7	94228205	C	G	475	0.00	330	**0.19**	391	**0.23**	208	**0.40**
7	99306707	G	A	221	0.00	231	**0.22**	202	**0.20**	136	**0.30**
7	104703929	C	G	318	0.00	322	0.06	381	0.00	274	0.00
7	117432354	C	T	298	0.00	467	0.01	422	0.08	367	0.00
8	17838154	G	C	420	0.00	223	0.05	242	**0.21**	67	**0.57**
8	22475022	G	C	314	0.00	258	**0.22**	301	0.01	215	0.00
8	42036512	G	C	294	0.00	371	0.03	379	0.08	358	0.12
8	42050647	G	C	328	0.00	408	0.02	428	0.10	391	0.14
8	54906316	C	G	331	0.00	381	0.01	371	**0.18**	228	**0.30**
8	55539174	C	G	467	0.00	474	**0.16**	608	0.00	522	0.00
8	68152455	G	C	439	0.00	443	0.14	640	0.00	483	0.00
8	124346447	C	A	477	0.00	590	0.04	619	0.11	651	**0.17**
8	125527994	G	T	763	0.00	946	**0.15**	1043	**0.23**	853	**0.34**
8	144652781	C	G	607	0.00	941	**0.16**	997	**0.16**	1115	**0.19**
9	33472295	C	G	267	0.00	397	0.15	367	0.12	376	**0.19**
9	74355039	C	G	314	0.00	492	0.04	496	0.14	414	**0.21**
9	99522522	G	T	680	0.00	1102	0.05	1205	0.00	1181	0.00
9	125639795	C	T	632	0.00	1078	0.02	993	0.10	759	**0.21**
9	139658186	C	T	234	0.00	139	**0.43**	136	**0.32**	107	**0.45**
9	139840549	G	A	441	0.00	444	0.03	322	0.13	295	**0.24**
9	140151397	C	T	313	0.00	314	0.11	275	**0.15**	270	**0.20**
10	5967338	C	G	267	0.00	375	0.04	379	**0.17**	329	0.14
10	8007253	G	A	501	0.00	858	0.06	1200	0.00	1416	0.00
10	17271827	G	C	351	0.00	437	0.12	516	0.11	594	0.13
10	18803274	G	C	465	0.00	509	0.02	727	0.12	519	**0.16**
10	26417386	T	A	390	0.00	552	0.06	730	0.06	786	0.09
10	26830582	G	C	582	0.00	917	0.02	1155	0.08	1226	0.10
10	28899733	C	G	623	0.00	976	0.08	1160	0.10	1070	0.14

10	28899733	C	T	631	0.00	1234	0.11	1452	0.11	1274	0.12
10	28903611	C	T	648	0.00	1344	0.11	1550	0.11	1391	0.11
10	37506650	GAG	-	910	0.00	646	0.12	1246	0.11	905	**0.18**
10	53667328	G	A	414	0.00	395	0.05	380	**0.18**	230	**0.31**
10	60577352	-	G	345	0.00	350	**0.26**	402	**0.20**	208	**0.41**
10	61665978	C	T	196	0.00	196	0.05	172	**0.18**	107	**0.39**
10	70404729	C	G	676	0.00	513	0.03	518	**0.19**	273	**0.37**
10	99214527	G	C	243	0.00	230	0.05	231	**0.23**	116	**0.38**
10	105939634	C	G	487	0.00	635	0.02	543	0.11	471	0.01
10	129846082	C	G	389	0.00	431	0.05	349	**0.22**	249	**0.36**
11	788159	G	A	243	0.00	371	0.05	168	**0.29**	68	**0.60**
11	18111013	C	T	295	0.00	339	0.03	288	**0.20**	161	**0.35**
11	27378985	C	G	611	0.00	436	0.04	388	**0.25**	231	**0.41**
11	48510647	C	G	295	0.00	337	0.06	223	**0.19**	131	**0.38**
11	71724307	C	G	151	0.00	177	0.02	140	0.08	136	**0.17**
11	73625460	G	A	329	0.00	538	0.04	600	0.08	541	**0.18**
11	74345660	G	C	388	0.00	534	0.08	661	0.00	605	0.00
11	76996233	C	G	553	0.00	725	0.02	570	0.14	527	**0.18**
11	103029408	C	G	464	0.00	302	0.03	259	**0.17**	186	**0.31**
11	108043757	G	A	324	0.00	251	0.05	261	0.15	160	**0.39**
11	123900793	C	G	190	0.00	234	**0.15**	251	0.01	213	0.00
11	126135714	C	G	270	0.00	366	0.15	445	0.01	447	0.00
12	2908310	G	C	280	0.00	339	**0.28**	296	**0.23**	233	**0.35**
12	8077019	G	C	447	0.00	462	**0.25**	539	**0.20**	280	**0.36**
12	9259138	C	T	343	0.00	494	0.00	412	0.00	297	0.11
12	27180324	G	A	631	0.00	1289	0.02	1124	0.11	1008	**0.17**
12	32786542	G	C	264	0.00	515	0.01	599	0.10	513	0.14
12	41966808	G	C	335	0.00	917	0.04	774	0.09	740	0.14
12	42729736	G	C	583	0.00	878	0.07	1122	0.00	1032	0.00
12	45173499	G	C	488	0.00	1014	0.02	896	0.08	990	0.09
12	47471653	A	G	688	0.00	2320	0.05	2165	0.05	2338	0.06
12	57537563	G	A	77	0.00	159	0.06	154	0.13	144	0.14
12	58140016	C	G	706	0.00	832	0.03	767	0.14	675	**0.20**
12	58201238	G	C	380	0.00	543	0.07	511	0.00	538	0.00
12	64814030	G	A	202	0.00	464	**0.16**	559	0.13	605	**0.16**
12	72955982	G	A	669	0.00	1656	0.02	1755	0.09	1784	0.12
12	76425090	C	G	274	0.01	707	0.00	838	0.01	883	0.09
12	77203578	C	T	489	0.00	891	0.02	794	0.10	827	0.15
12	86373975	C	G	455	0.00	875	0.02	834	0.11	832	0.14
12	95603492	G	A	416	0.00	556	0.06	714	0.01	694	0.00
12	105260249	G	A	358	0.00	259	0.02	342	0.06	283	0.13
12	105534156	C	G	653	0.00	957	0.03	1053	0.12	1014	0.14
12	106821074	G	C	574	0.00	835	0.05	1131	0.00	996	0.00

12	123047215	T	G	741	0.00	1397	0.12	1854	0.13	1736	0.14
12	124303551	C	G	359	0.00	986	0.02	884	0.10	904	0.14
12	132629472	G	T	391	0.00	925	0.02	991	0.09	1127	0.13
12	133220503	C	G	321	0.00	1144	0.02	1059	0.10	1048	0.14
13	34399960	G	C	585	0.00	522	**0.28**	745	**0.28**	584	**0.37**
13	36905672	C	A	384	0.00	1037	0.01	1115	0.07	1081	0.08
13	41333148	T	C	233	0.00	172	**0.22**	195	**0.22**	97	**0.40**
13	43500517	T	A	441	0.00	361	**0.25**	361	**0.24**	251	**0.36**
13	52707919	C	T	309	0.00	240	0.06	252	**0.18**	119	**0.42**
13	53313788	C	T	275	0.00	421	0.05	391	0.01	309	0.00
13	77785332	C	G	619	0.00	342	**0.41**	326	**0.24**	211	**0.38**
13	96579335	G	A	342	0.00	236	0.05	231	**0.20**	160	**0.29**
13	96601606	G	A	229	0.00	103	**0.20**	127	**0.20**	92	**0.21**
13	100635089	C	G	250	0.00	242	0.04	244	**0.21**	163	**0.36**
14	101349370	C	G	115	0.00	174	0.05	110	**0.20**	65	**0.36**
15	40683705	G	T	429	0.00	469	0.03	473	**0.19**	212	**0.39**
15	51507930	C	T	550	0.00	583	**0.20**	610	**0.17**	523	**0.26**
15	63089477	G	A	136	0.00	125	0.02	118	0.13	76	**0.30**
15	75652979	G	A	73	0.00	87	0.02	74	**0.20**	56	**0.40**
15	82336165	G	A	600	0.00	425	0.01	423	0.03	225	**0.35**
15	84685134	G	C	315	0.00	344	0.03	270	**0.17**	159	**0.39**
15	89073905	G	C	303	0.00	318	0.02	246	**0.17**	176	**0.29**
15	89453088	C	T	393	0.00	738	0.09	771	0.00	663	0.00
16	4910966	G	C	120	0.00	168	0.03	170	0.13	126	**0.22**
16	5140312	C	G	331	0.00	655	0.06	656	0.06	571	0.06
16	8736061	G	A	400	0.00	620	0.04	618	**0.17**	473	**0.28**
16	11945311	G	C	162	0.01	164	0.01	207	**0.16**	168	**0.24**
16	55727968	G	C	348	0.00	389	**0.20**	418	**0.18**	257	**0.36**
16	61747806	G	C	523	0.00	593	**0.31**	592	**0.30**	498	**0.39**
16	67006596	G	C	132	0.00	275	0.14	285	0.00	267	0.00
16	67178755	C	G	223	0.01	370	0.06	430	0.00	327	0.00
16	68358733	G	C	248	0.01	278	0.03	270	**0.17**	169	**0.28**
16	86602303	C	A	234	0.00	356	0.03	253	**0.17**	224	**0.26**
16	90106831	G	C	313	0.00	372	0.03	303	**0.21**	187	**0.29**
17	10305036	C	G	427	0.00	578	0.05	461	**0.19**	259	**0.29**
17	11984833	-	TATT	328	0.00	100	**0.44**	145	**0.32**	33	**0.76**
17	19319105	C	G	330	0.00	1069	0.02	883	0.12	933	**0.15**
17	19861735	C	G	527	0.00	1446	0.01	1509	0.06	1573	0.10
17	42399135	C	T	333	0.00	290	0.05	186	**0.20**	127	**0.37**
17	42477211	C	T	231	0.00	302	0.03	326	**0.18**	156	**0.38**
17	48694894	C	A	275	0.00	328	0.12	277	0.15	272	0.12
17	63534409	G	A	350	0.00	657	**0.18**	597	**0.16**	566	**0.23**
17	71468283	A	C	276	0.00	307	0.15	261	0.14	258	**0.17**

17	74392387	C	G	289	0.00	549	0.02	449	0.13	406	**0.20**
17	80042741	C	T	295	0.00	450	**0.23**	359	**0.17**	430	**0.22**
18	2606483	G	A	786	0.00	294	0.05	291	**0.19**	189	**0.37**
18	19095513	G	C	345	0.00	549	0.02	513	**0.16**	366	**0.25**
18	32833605	G	C	388	0.00	267	**0.20**	336	**0.16**	298	**0.25**
18	51820508	G	T	343	0.00	291	0.01	292	0.11	260	0.12
19	1820367	C	G	296	0.00	419	**0.20**	433	**0.29**	354	**0.41**
19	2834682	G	C	149	0.00	148	0.06	129	**0.22**	64	**0.50**
19	5621365	C	G	512	0.01	545	0.14	552	0.01	445	0.00
19	8145962	G	T	273	0.00	450	0.01	395	0.12	328	**0.17**
19	8591716	C	G	568	0.00	573	0.02	509	0.10	436	**0.20**
19	9801422	C	G	221	0.00	234	0.10	230	**0.18**	209	**0.19**
19	12691855	G	C	440	0.00	493	0.04	555	0.13	529	**0.17**
19	15280938	C	G	164	0.00	454	**0.20**	489	0.02	482	0.00
19	15760796	C	G	316	0.01	773	0.01	726	0.01	593	0.11
19	20117467	C	G	603	0.00	561	0.03	592	0.12	355	**0.28**
19	37309910	C	T	573	0.00	948	0.03	502	**0.20**	343	**0.32**
19	38380719	C	A	319	0.00	597	0.14	783	0.12	728	0.14
19	38959760	G	A	171	0.00	303	**0.16**	334	0.11	375	0.13
19	40364363	G	C	422	0.00	629	0.00	441	0.02	254	**0.29**
19	41019431	C	T	519	0.00	717	0.07	409	0.00	326	0.00
19	45448421	C	A	362	0.00	457	0.08	567	0.00	472	0.00
19	46838234	G	T	248	0.00	353	**0.18**	490	0.02	401	0.00
19	48919307	G	A	558	0.00	484	**0.26**	438	**0.23**	300	**0.39**
19	49224168	C	T	102	0.00	70	0.03	52	**0.19**	39	**0.29**
19	50204090	C	G	174	0.00	252	0.02	202	0.14	156	0.03
19	51645894	G	A	228	0.00	296	0.03	255	**0.22**	135	**0.46**
19	54613474	G	A	457	0.00	872	0.11	980	0.00	964	0.00
19	54973574	C	G	249	0.00	409	0.12	507	0.01	481	0.00
19	55756544	C	T	382	0.00	581	0.03	442	0.14	429	**0.17**
20	5170827	C	T	335	0.00	383	**0.20**	425	**0.19**	306	**0.29**
20	30365363	C	G	386	0.00	544	0.14	688	0.12	441	**0.22**
20	35627291	C	G	411	0.00	654	0.02	585	0.12	499	**0.19**
20	57276205	C	G	84	0.00	90	0.08	120	0.00	126	0.01
20	57769246	C	G	284	0.00	697	0.02	541	0.12	535	**0.20**
21	34927032	C	T	642	0.00	552	0.04	552	0.12	512	**0.17**
21	36171710	-	T	436	0.00	709	0.13	733	0.13	668	**0.17**
21	36252965	-	CAC	448	0.00	401	**0.29**	545	**0.30**	388	**0.33**
21	40778461	C	G	486	0.00	397	0.15	464	0.09	405	**0.19**
21	43291672	C	G	321	0.00	665	0.02	492	0.13	389	**0.21**
22	30765478	G	T	341	0.00	628	0.02	474	0.11	448	**0.17**
22	30822725	G	A	173	0.00	324	**0.18**	335	0.14	273	**0.28**
22	32754218	G	T	212	0.00	626	0.01	430	**0.16**	425	**0.17**

22	39001354	C	G	331	0.00	381	0.13	471	0.01	423	0.00
22	39441166	G	A	583	0.01	1158	**0.20**	1079	**0.20**	1002	**0.26**
22	50588043	G	C	304	0.00	533	0.12	664	0.00	603	0.00
X	10442701	C	T	457	0.00	695	0.06	1334	0.00	1481	0.00
X	24906131	G	C	462	0.00	838	0.02	921	0.08	973	0.12
X	32382724	G	A	536	0.00	509	0.07	475	**0.19**	278	**0.33**
X	38156605	C	T	334	0.00	334	0.04	374	**0.17**	203	**0.36**
X	54276472	G	C	347	0.00	392	0.08	583	0.00	515	0.00
X	70756079	G	T	428	0.00	609	0.05	778	0.14	549	**0.26**
X	78010805	C	A	425	0.00	384	0.02	286	0.11	199	0.01
X	107938125	C	G	248	0.00	296	**0.25**	251	**0.22**	155	**0.33**
X	118257551	C	T	436	0.00	927	0.14	894	0.14	731	**0.20**
X	128621106	C	G	368	0.00	397	0.03	308	0.10	169	**0.33**
X	148037565	C	T	275	0.00	340	0.02	323	0.12	237	**0.30**
X	154091387	C	T	405	0.00	563	**0.32**	439	**0.38**	271	**0.54**
1	11562830	G	T	119	0.00	214	**0.16**	180	**0.22**	162	**0.24**
1	37291371	G	C	166	0.00	288	0.06	233	**0.19**	160	**0.38**
1	67792439	A	C	363	0.00	281	**0.23**	318	**0.22**	150	**0.41**
1	114949579	C	T	466	0.01	311	**0.22**	378	**0.20**	238	**0.31**
1	155629738	G	C	197	0.00	285	**0.18**	303	**0.19**	224	**0.22**
1	204379375	C	T	378	0.00	422	0.12	546	0.00	481	0.00
1	214571293	C	T	679	0.01	675	**0.17**	754	**0.17**	584	**0.24**
2	48573736	C	G	658	0.00	741	0.03	713	0.11	558	**0.21**
2	68692258	C	T	356	0.00	241	0.07	258	**0.15**	184	**0.26**
2	74448595	C	G	312	0.01	327	**0.20**	328	**0.17**	237	**0.28**
2	100065832	C	A	518	0.00	827	0.02	806	0.13	730	**0.20**
2	131521422	G	A	327	0.00	421	0.14	427	0.00	572	0.00
2	179498586	G	C	445	0.00	674	0.01	552	0.02	451	0.11
2	234638424	C	G	416	0.00	1004	0.02	628	**0.16**	455	**0.21**
3	17550044	C	T	298	0.00	266	**0.17**	281	**0.18**	120	**0.34**
3	66431013	G	C	398	0.00	413	**0.16**	378	**0.18**	190	**0.33**
3	69987147	G	A	390	0.00	201	**0.19**	144	0.01	143	0.00
3	128616533	T	A	299	0.00	523	**0.16**	558	**0.15**	539	**0.19**
3	167159970	G	C	242	0.00	239	0.04	235	**0.25**	176	**0.34**
3	185985544	G	C	269	0.00	563	**0.15**	638	0.15	537	**0.18**
4	62758580	G	A	225	0.00	164	**0.15**	233	0.00	175	0.00
4	66217163	C	A	278	0.00	863	0.02	858	0.08	864	0.07
4	68797749	C	T	292	0.00	1008	0.00	975	0.01	956	0.06
4	155665566	G	A	263	0.01	245	0.04	196	**0.23**	127	**0.34**
4	185941474	G	C	186	0.00	246	0.05	276	0.00	246	0.00
5	21752007	C	T	188	0.00	420	0.01	479	0.02	457	0.10
5	86708579	C	G	312	0.00	264	0.03	265	**0.21**	174	**0.28**
5	140306606	G	C	325	0.00	398	0.02	280	**0.21**	156	**0.41**

5	141381648	G	C	284	0.01	250	0.01	182	**0.18**	109	**0.32**
5	147281235	C	T	300	0.00	310	0.04	230	**0.20**	150	**0.32**
5	171520439	C	T	174	0.00	204	0.06	150	**0.21**	101	**0.37**
5	176295612	G	C	221	0.00	366	0.03	189	**0.20**	150	**0.39**
6	32945708	G	A	172	0.00	154	0.02	157	0.13	136	**0.20**
6	76624648	C	G	275	0.00	455	0.03	390	**0.15**	330	**0.18**
6	83754323	C	G	275	0.00	393	0.02	410	0.08	353	**0.21**
6	97562220	G	C	126	0.00	153	**0.15**	223	0.00	164	0.00
6	117710989	C	A	268	0.00	387	0.11	419	0.13	363	**0.18**
6	160482607	C	A	462	0.00	1022	0.06	1087	0.00	1105	0.00
7	7283171	C	G	653	0.00	650	0.04	577	**0.16**	424	**0.24**
7	18066623	G	C	248	0.00	348	0.03	347	0.15	215	**0.30**
7	21901510	G	C	325	0.00	480	0.04	343	**0.19**	334	**0.21**
7	106509129	C	T	342	0.00	265	0.07	168	**0.28**	122	**0.36**
7	120979151	C	A	409	0.00	355	0.04	378	**0.16**	195	**0.33**
7	127252006	G	C	73	0.00	112	0.02	97	**0.21**	60	**0.33**
7	143748299	G	C	70	0.00	113	0.03	82	**0.21**	48	**0.36**
8	57876375	G	C	409	0.00	692	0.02	609	0.15	424	**0.27**
8	107773435	C	T	515	0.00	830	0.02	792	0.10	691	0.14
8	143866744	C	T	161	0.00	170	**0.16**	194	0.13	205	**0.18**
9	34616031	G	C	343	0.00	617	0.05	560	0.15	483	**0.20**
9	107360846	G	C	318	0.00	523	0.15	505	0.12	411	**0.24**
9	116136467	C	G	76	0.01	132	0.01	94	**0.17**	118	0.13
9	124929126	G	A	237	0.00	668	0.05	577	0.00	615	0.00
10	1149768	C	T	936	0.00	994	0.06	1245	0.00	1177	0.00
10	5248310	G	T	104	0.00	145	0.05	162	0.00	138	0.00
10	14950552	G	A	567	0.00	1105	0.02	1162	0.08	1231	0.10
10	15255691	C	A	390	0.00	966	0.01	974	0.07	1114	0.09
10	18840793	C	G	410	0.00	626	0.13	889	0.00	832	0.00
10	68940104	G	A	388	0.00	433	0.02	346	**0.18**	203	**0.38**
10	124358488	C	G	344	0.00	615	0.00	540	0.03	321	**0.30**
10	129901667	C	G	322	0.00	387	0.09	457	0.00	302	0.00
11	209940	G	A	336	0.00	426	0.03	223	**0.22**	78	**0.60**
11	209941	G	T	327	0.00	418	0.03	218	**0.22**	78	**0.60**
11	49221947	G	A	485	0.00	291	**0.25**	326	**0.18**	234	**0.30**
11	59421497	C	A	444	0.00	218	**0.40**	245	**0.46**	80	**0.75**
11	72295658	C	A	284	0.00	1094	0.03	898	0.12	1069	**0.18**
11	82868503	G	C	412	0.00	143	0.04	127	0.09	168	**0.16**
11	101362454	-	T	501	0.00	606	0.06	619	0.00	521	0.00
12	1023215	G	C	216	0.00	270	0.15	329	0.00	227	0.00
12	4705842	G	C	474	0.00	638	0.04	469	**0.21**	268	**0.39**
12	51347841	C	G	565	0.00	2151	0.01	2269	0.06	2342	0.09
12	51495752	C	T	403	0.00	843	0.01	685	0.10	730	0.12

12	51634752	C	G	235	0.00	522	0.02	643	0.08	610	0.10
12	57602598	C	T	213	0.00	264	**0.16**	264	**0.17**	241	**0.22**
12	101745836	G	C	517	0.00	992	0.02	994	0.08	895	0.13
13	32953632	C	G	496	0.00	476	0.04	524	0.11	476	0.14
14	105419879	C	G	422	0.00	448	**0.26**	448	**0.24**	305	**0.33**
15	43814435	C	T	258	0.00	409	0.10	406	0.00	353	0.00
15	49127094	C	A	191	0.00	183	0.02	156	**0.22**	96	**0.38**
15	54306622	G	A	475	0.00	622	0.01	519	0.14	367	**0.24**
16	2126076	C	G	155	0.00	411	0.01	317	**0.16**	272	**0.22**
16	8722756	G	T	268	0.00	471	0.05	462	**0.19**	341	**0.27**
16	31092215	C	G	167	0.00	225	**0.18**	226	**0.18**	185	**0.26**
16	70889175	C	T	184	0.00	701	0.01	566	0.12	569	**0.16**
17	7578263	G	A	399	0.00	413	**0.47**	385	**0.47**	139	**0.76**
17	7798764	-	C	137	0.01	154	0.01	141	**0.18**	76	**0.34**
17	26708353	G	A	351	0.00	288	0.05	179	**0.21**	136	**0.33**
17	46862401	G	C	236	0.00	536	0.14	501	0.09	457	**0.19**
17	74080340	C	T	250	0.00	438	0.02	399	0.08	429	**0.20**
17	74468028	G	A	473	0.00	921	**0.16**	784	**0.16**	900	**0.19**
17	74729222	C	T	479	0.00	556	0.14	611	0.14	597	**0.17**
18	44109147	C	G	204	0.00	852	0.01	754	0.04	785	0.11
18	48591846	G	C	486	0.01	1110	0.03	1136	0.13	1050	**0.17**
18	50976882	G	C	377	0.00	593	0.00	688	0.02	509	0.13
19	37149226	C	G	474	0.00	422	**0.15**	324	**0.19**	201	**0.36**
19	38126246	G	A	432	0.00	648	0.06	669	0.00	677	0.00
19	40376946	G	A	128	0.00	151	0.07	103	0.03	70	0.10
19	54625919	C	G	277	0.00	590	0.05	675	0.00	628	0.00
19	54759980	C	G	208	0.00	576	**0.19**	520	**0.20**	547	**0.19**
19	55450836	C	T	256	0.00	535	0.13	579	0.01	560	0.00
19	57326172	G	T	344	0.00	450	**0.40**	401	**0.42**	343	**0.49**
20	37400403	C	T	432	0.00	519	**0.17**	441	**0.15**	451	**0.19**
20	43629817	G	A	569	0.01	1072	0.03	948	0.13	788	**0.22**
21	10951380	G	T	335	0.00	141	0.13	151	0.10	94	**0.24**
22	22161983	C	G	476	0.00	636	0.02	635	0.13	581	**0.20**
22	22899240	G	A	209	0.00	373	**0.15**	304	0.09	299	0.12
22	39176944	G	C	152	0.00	227	**0.19**	243	0.12	205	**0.24**
22	42783071	C	T	427	0.00	565	**0.16**	506	**0.16**	557	**0.21**
22	45802352	C	G	596	0.00	610	0.12	600	0.13	539	**0.19**
X	12904612	G	C	397	0.00	434	0.05	380	**0.20**	234	**0.40**
X	57146462	C	T	96	0.00	194	0.03	186	**0.19**	89	**0.41**
X	63412007	T	G	150	0.00	209	0.14	183	0.01	127	0.00
X	64936704	G	T	167	0.00	185	**0.24**	136	**0.24**	35	**0.62**
X	65392277	A	G	340	0.00	526	0.06	366	**0.29**	101	**0.65**
X	102885082	G	C	92	0.00	148	0.08	135	0.00	108	0.00

X	147063094	C	T	451	0.00	481	**0.38**	301	**0.42**	228	**0.55**
1	110557452	G	A	428	0.00	319	**0.16**	339	**0.22**	159	**0.32**
1	147121984	C	T	209	0.00	369	0.03	283	**0.19**	215	**0.29**
1	152277822	T	C	187	0.01	707	**0.21**	644	**0.22**	525	**0.23**
1	155911572	G	A	181	0.00	300	0.08	311	0.00	316	0.00
1	157490895	C	T	370	0.00	654	0.06	770	0.00	780	0.00
1	159284102	G	A	244	0.00	538	0.06	546	0.00	470	0.00
1	167334732	G	A	340	0.00	371	**0.20**	429	**0.17**	288	**0.27**
1	197390707	C	T	410	0.00	366	**0.18**	410	**0.18**	302	**0.27**
1	203098233	C	T	376	0.00	960	0.03	781	**0.18**	664	**0.25**
1	230845796	C	T	197	0.00	408	0.14	393	0.01	443	0.00
2	3651951	G	A	441	0.00	1163	0.13	1136	0.11	1169	0.13
2	26152328	G	A	180	0.00	280	0.03	252	**0.17**	194	**0.22**
2	97267977	G	C	283	0.00	556	0.10	615	0.00	516	0.00
2	170417064	G	T	683	0.00	975	0.03	836	0.13	608	**0.23**
2	173885386	G	A	506	0.00	890	0.02	621	**0.15**	425	**0.28**
2	205989070	C	T	368	0.01	500	**0.17**	427	**0.22**	403	**0.25**
2	235943745	G	A	420	0.00	525	**0.31**	524	**0.17**	435	**0.27**
3	9962640	C	T	62	0.00	69	**0.21**	55	**0.28**	53	**0.35**
3	113169342	G	A	229	0.00	171	0.15	160	**0.25**	147	**0.37**
3	113375798	C	T	472	0.00	1041	0.02	1102	0.11	928	**0.18**
3	130103987	C	A	292	0.00	499	0.13	582	0.12	536	**0.18**
3	130733159	C	G	366	0.00	623	**0.16**	639	**0.20**	586	**0.29**
3	170843782	C	T	424	0.00	452	**0.18**	499	0.14	572	**0.16**
3	179051133	G	A	578	0.00	654	0.02	808	0.10	695	0.13
4	71895094	G	A	507	0.00	1056	0.01	1077	0.05	1116	0.07
4	128743929	G	A	443	0.00	464	0.03	355	**0.20**	207	**0.34**
4	190884260	G	A	861	0.00	865	0.03	855	**0.17**	509	**0.34**
5	5146403	G	C	299	0.00	444	**0.20**	365	**0.43**	402	**0.46**
5	55155408	C	G	258	0.00	453	0.10	545	0.01	561	0.00
5	149431566	C	G	141	0.00	190	0.02	130	**0.18**	83	**0.34**
5	167671691	G	T	425	0.00	277	**0.16**	272	**0.24**	147	**0.34**
6	26027403	G	A	356	0.00	186	**0.18**	244	**0.17**	202	**0.23**
6	29598278	G	A	375	0.00	383	0.03	295	**0.15**	239	**0.22**
6	56919460	G	C	885	0.00	1700	0.00	1978	0.01	1675	0.06
6	76373194	G	A	479	0.00	331	0.07	373	0.13	268	**0.18**
6	102130457	C	T	670	0.00	1326	0.02	1237	0.12	1095	**0.18**
6	116289799	C	T	593	0.00	994	0.02	1028	0.10	935	**0.17**
6	129714373	A	G	490	0.00	853	0.08	1050	0.10	1076	0.12
6	129835667	G	A	467	0.00	1214	0.01	1394	0.09	1209	0.14
6	137147538	C	T	210	0.00	230	**0.18**	339	**0.24**	258	**0.25**
6	145142071	C	T	561	0.00	1274	0.02	1576	0.08	1482	0.11
6	155606304	G	A	412	0.00	946	0.07	1217	0.05	1261	0.07

7	31008460	C	T	252	0.00	582	0.13	643	0.01	609	0.00
7	42018264	G	A	213	0.01	391	0.02	339	0.11	247	**0.25**
7	55863654	C	T	277	0.01	168	**0.16**	206	**0.18**	134	**0.30**
7	86569402	G	C	319	0.01	225	0.02	183	0.13	116	**0.33**
7	100646018	G	A	141	0.01	339	0.13	339	**0.16**	233	0.13
8	61707581	G	A	696	0.00	288	0.02	420	0.10	263	**0.24**
8	97156803	G	A	399	0.00	728	**0.23**	606	**0.25**	556	**0.41**
8	101936198	C	G	577	0.00	571	0.14	553	**0.27**	425	**0.38**
8	144406175	G	A	290	0.00	225	**0.15**	267	0.01	301	0.00
8	145615877	G	A	94	0.00	108	0.11	182	0.00	176	0.00
8	146068040	G	A	407	0.00	316	**0.44**	296	**0.53**	190	**0.71**
9	38395865	C	T	272	0.00	569	0.04	494	**0.16**	396	**0.19**
9	39144334	G	A	102	0.00	295	0.03	284	0.14	198	**0.23**
9	125512408	C	T	158	0.01	507	0.08	471	0.07	354	0.09
10	11805336	G	A	430	0.00	1591	0.01	1471	0.05	1935	0.07
10	17142095	G	A	335	0.00	437	0.03	507	0.10	437	0.11
10	25755603	C	A	282	0.00	326	**0.16**	392	0.10	380	**0.17**
10	27382620	C	T	494	0.00	1040	0.02	1095	0.10	1107	0.12
10	51465097	G	A	57	0.00	346	**0.17**	405	**0.19**	259	**0.20**
10	99683057	G	A	536	0.00	531	**0.24**	515	**0.25**	352	**0.41**
11	619711	C	T	302	0.00	242	0.06	141	**0.29**	54	**0.66**
11	33053922	C	G	404	0.00	538	0.01	622	0.08	584	0.14
11	57582951	G	A	386	0.00	397	**0.28**	486	0.00	389	0.00
11	113860446	C	T	105	0.00	152	0.08	156	0.00	148	0.00
11	121485605	C	G	301	0.00	519	**0.27**	424	**0.25**	278	**0.39**
12	331737	G	A	140	0.00	261	0.01	205	0.15	122	**0.39**
12	14775089	C	T	320	0.00	429	0.04	284	**0.22**	191	**0.38**
12	40692254	C	T	535	0.00	725	0.03	742	0.09	617	**0.17**
12	52407933	G	A	97	0.00	265	0.08	372	0.01	377	0.00
12	52867105	G	A	50	0.00	68	0.08	72	**0.16**	52	**0.22**
12	78415530	A	T	438	0.00	915	0.02	905	0.08	911	0.11
12	102038506	G	C	410	0.00	844	0.02	1049	0.07	864	0.14
12	104481804	C	T	423	0.00	660	0.03	657	0.13	610	0.14
12	109898516	C	T	615	0.00	1340	0.02	1118	0.10	1361	0.13
12	123661252	C	T	506	0.00	830	0.02	823	0.08	823	**0.16**
12	123810711	G	A	584	0.00	1027	0.03	1179	0.09	1100	0.14
12	133781077	C	T	561	0.00	756	0.02	694	0.12	822	0.12
13	25482206	C	T	687	0.00	1249	0.02	1264	0.10	1200	0.12
13	29608198	G	A	340	0.00	1196	0.02	1226	0.09	1109	0.11
13	61987083	G	A	490	0.00	1549	0.01	1372	0.07	1314	0.11
13	113852561	C	T	307	0.00	171	0.08	174	**0.21**	46	**0.60**
14	20586093	A	G	403	0.00	646	0.04	474	**0.21**	283	**0.38**
14	47120652	G	T	190	0.00	190	**0.23**	159	**0.23**	81	**0.34**

14	51383438	C	A	328	0.00	379	0.05	301	**0.21**	182	**0.37**
15	77472727	C	T	659	0.00	922	0.00	977	0.00	603	0.08
15	78301381	G	A	317	0.01	352	0.04	312	**0.17**	160	**0.36**
16	20975381	C	T	349	0.00	820	0.02	779	**0.16**	573	**0.26**
16	56397994	G	C	556	0.00	1200	0.01	921	0.10	943	0.14
16	68225635	T	C	682	0.00	686	**0.19**	708	**0.18**	510	**0.28**
16	84806198	C	T	598	0.00	1048	0.13	1056	**0.16**	768	**0.25**
16	89249964	G	C	169	0.00	233	0.08	199	0.00	179	0.00
17	5307486	C	T	552	0.00	583	0.03	551	**0.19**	334	**0.31**
18	32920372	G	C	148	0.00	112	0.03	74	**0.23**	59	**0.30**
18	65180727	C	T	441	0.00	385	0.04	497	**0.18**	264	**0.33**
19	11024645	C	T	410	0.00	628	0.04	498	**0.16**	289	**0.35**
19	34791750	C	A	262	0.00	458	0.09	355	**0.39**	268	**0.52**
19	42866658	G	T	579	0.00	739	0.04	482	**0.20**	361	**0.36**
19	50979162	G	T	655	0.00	594	**0.17**	624	0.01	556	0.00
19	53384377	G	A	278	0.02	178	**0.22**	167	**0.22**	104	**0.35**
19	56244150	C	T	491	0.00	1067	0.15	1134	0.13	997	**0.16**
20	19977369	G	A	348	0.00	494	0.04	459	**0.17**	312	**0.24**
20	31372629	C	G	530	0.00	1942	0.03	1650	0.15	1569	**0.20**
20	43129048	G	A	185	0.01	236	0.02	225	0.13	226	**0.18**
20	56227112	G	C	407	0.00	566	**0.17**	574	0.14	551	**0.23**
21	34142159	C	T	1076	0.00	1355	0.09	1672	0.01	1576	0.00
22	39884105	C	G	595	0.00	720	0.02	532	**0.15**	448	**0.22**
X	27998036	G	T	192	0.01	174	0.12	244	0.01	162	0.00
X	48369849	C	T	183	0.00	482	0.05	388	**0.20**	276	**0.34**
X	122772861	C	T	509	0.00	930	0.03	879	0.15	833	**0.20**
X	125954720	C	T	210	0.00	315	**0.22**	267	**0.20**	172	**0.39**
X	153199893	G	C	130	0.00	214	0.04	165	**0.20**	176	**0.19**

Supplementary Table S9. List of somatic mutations and allele frequencies for P123.

ALN: axillary lymph node; Ref: reference allele; Alt: alternative allele; RD: read depth; BAF: alternative allele frequency.

				Normal		DCIS		Primary tumor		ALN metastasis	
**Chr**	**Start position**	**Ref**	**Alt**	**RD**	**BAF**	**RD**	**BAF**	**RD**	**BAF**	**RD**	**BAF**
1	46649948	C	T	297	0.00	127	0.46	111	0.53	195	0.15
1	93826121	G	C	316	0.00	161	0.36	143	0.45	283	0.13
1	155220084	G	A	202	0.00	160	0.16	162	0.15	105	0.06
1	159015175	C	G	105	0.00	194	0.21	173	0.24	191	0.12
2	32740611	T	A	408	0.00	393	0.25	252	0.41	438	0.07
2	68717387	C	G	590	0.00	411	0.29	401	0.36	537	0.04
2	85777095	G	A	451	0.00	281	0.28	341	0.29	379	0.06
2	111907653	-	GCCCATA	595	0.00	469	0.24	435	0.30	609	0.05
2	167141186	C	T	515	0.00	311	0.22	288	0.22	333	0.10
3	113377477	G	A	837	0.00	707	0.30	748	0.32	864	0.12
4	79442782	G	C	383	0.01	306	0.30	345	0.30	351	0.09
4	100458863	C	T	339	0.00	277	0.27	240	0.31	305	0.05
4	113484304	G	C	192	0.00	166	0.24	151	0.26	172	0.06
5	10423918	G	C	575	0.00	372	0.32	424	0.33	609	0.07
5	37227700	C	G	1003	0.00	765	0.16	711	0.27	844	0.11
5	37227736	C	T	1013	0.00	797	0.16	726	0.29	887	0.11
6	30916561	A	T	208	0.00	126	0.20	177	0.27	148	0.06
6	32046852	C	T	179	0.01	67	0.34	60	0.35	80	0.07
6	132967009	A	T	526	0.00	450	0.27	385	0.30	507	0.07
7	2473380	G	A	133	0.00	106	0.26	105	0.36	106	0.11
7	48312055	C	T	674	0.00	484	0.31	473	0.35	714	0.09
7	48312619	C	T	437	0.00	336	0.28	310	0.31	422	0.07
7	48314304	C	T	535	0.00	398	0.33	408	0.34	601	0.06
7	94259049	G	C	432	0.00	348	0.24	354	0.32	373	0.05
8	28384677	G	A	315	0.00	134	0.36	108	0.51	227	0.07
8	67752482	AGTA	-	868	0.00	789	0.24	872	0.25	892	0.09
8	127569333	G	A	690	0.00	436	0.27	461	0.34	378	0.13
9	91159390	C	G	825	0.00	480	0.30	433	0.40	566	0.12
9	108145551	G	C	408	0.00	248	0.28	225	0.31	413	0.10
10	24508801	C	G	434	0.00	358	0.21	387	0.27	555	0.07
11	82571051	C	T	908	0.00	728	0.30	669	0.28	909	0.10
12	977908	C	A	256	0.00	245	0.15	211	0.27	258	0.09
12	6626989	G	C	89	0.00	76	0.23	111	0.21	93	0.12
12	57918089	G	A	448	0.00	282	0.17	314	0.26	315	0.12

12	80982114	G	A	423	0.00	295	0.31	282	0.37	382	0.09
12	86276100	G	A	681	0.00	329	0.33	364	0.34	427	0.11
12	105538179	C	T	802	0.00	583	0.31	567	0.34	716	0.09
14	21838648	C	T	406	0.00	313	0.28	318	0.32	427	0.05
14	35579793	C	A	362	0.00	315	0.24	285	0.24	398	0.10
14	50605333	C	T	615	0.00	372	0.22	381	0.22	428	0.12
14	51721183	G	C	619	0.00	335	0.30	463	0.30	446	0.06
15	66215259	C	T	546	0.00	290	0.25	298	0.31	346	0.08
15	101555577	A	G	517	0.00	506	0.22	459	0.25	560	0.10
16	2348465	C	G	166	0.00	164	0.32	174	0.35	149	0.08
16	2827056	C	G	370	0.00	228	0.28	230	0.38	218	0.11
17	59984916	G	A	210	0.00	255	0.23	225	0.31	224	0.10
17	76456055	C	A	869	0.00	501	0.26	566	0.32	512	0.11
19	3733901	C	G	722	0.00	330	0.39	355	0.43	579	0.11
20	17434414	G	A	505	0.00	244	0.28	262	0.27	280	0.11
20	62366030	C	T	654	0.00	409	0.28	480	0.31	448	0.10
21	30332898	G	C	620	0.00	529	0.27	497	0.27	647	0.10
X	12735869	C	T	979	0.00	521	0.29	562	0.31	549	0.11
X	43652716	G	A	511	0.00	525	0.28	537	0.30	647	0.09
X	54842006	C	T	328	0.00	222	0.24	301	0.26	215	0.13
X	73811476	C	G	491	0.00	499	0.29	425	0.31	558	0.08
X	129546770	G	A	317	0.00	138	0.23	169	0.28	134	0.06
4	146824038	G	A	212	0.00	156	0.09	173	0.06	120	0.00
5	145610390	G	A	342	0.00	353	0.06	425	0.04	388	0.01
7	129666107	C	G	351	0.00	435	0.12	453	0.14	424	0.03
7	136700102	T	C	261	0.00	316	0.08	314	0.17	291	0.03
8	38913169	C	G	760	0.00	1201	0.11	1141	0.09	988	0.04
X	96139940	G	C	374	0.00	345	0.23	326	0.26	437	0.02
6	30864460	C	T	439	0.00	320	0.02	351	0.08	248	0
9	70177977	G	T	66	0.00	68	0.15	59	0.13	67	0.08
14	95058476	C	T	382	0.00	249	0.23	229	0.25	256	0.09
1	26370796	C	T	215	0.00	70	0.53	88	0.46	142	0.08
2	233899041	C	T	516	0.00	250	0.21	287	0.25	291	0.09
4	1018787	C	T	103	0.01	105	0.26	117	0.31	109	0.06
4	36212143	C	T	616	0.00	493	0.21	385	0.29	522	0.07
5	33947301	G	A	251	0.00	292	0.23	297	0.27	339	0.09
5	156899767	C	T	348	0.00	294	0.11	357	0.12	300	0.03
6	52995607	C	T	276	0.00	325	0.26	346	0.30	345	0.10
7	99260482	C	T	297	0.00	317	0.11	319	0.12	359	0.02
9	96021359	C	T	430	0.00	293	0.23	290	0.25	302	0.03
11	7960381	T	A	258	0.00	246	0.25	264	0.30	295	0.11
11	62985192	G	A	502	0.00	422	0.30	461	0.25	486	0.08
11	68183842	G	A	299	0.00	180	0.26	190	0.37	158	0.12

11	105881195	C	T	429	0.00	374	0.25	374	0.24	417	0.11
12	6637944	G	A	592	0.00	229	0.33	292	0.33	307	0.11
12	113442876	G	A	527	0.00	454	0.23	505	0.24	475	0.12
14	20181989	G	C	77	0.00	94	0.12	76	0.08	73	0.10
16	22132351	C	T	671	0.00	873	0.10	1067	0.07	854	0.04
17	74005512	C	T	626	0.00	448	0.28	502	0.30	466	0.08
19	4512187	G	A	121	0.00	113	0.02	191	0.03	142	0.05
21	43319448	G	A	735	0.00	506	0.20	464	0.30	571	0.08
X	128657216	G	A	654	0.00	422	0.20	438	0.24	375	0.02
X	147733568	G	C	676	0.00	517	0.30	617	0.27	651	0.10

Supplementary Table S10. Genes affected by mutations exclusively in the metastases of the studied patients.

**Genes affected by mutations exclusively in metastases**				
**P4**	**P8**	**P11**	**P15**	**P46**
**SZT2**	NBAS	SPSB1	C15orf43	TIAM2
**ROR1**	OTOF	PRAMEF2	DCC	TIAM2
**ACADM**	NCKAP5	PRDM2		A2M
**ST6GALNAC3**	FAT4	IFI44L		PHLDA1
**LAMB3**	VCAN	GPATCH2		CYP4F3
**DUSP10**	C6orf52	KCNK1		FCGBP
**AGT**	ARID1B	OR2M4		TTN
**GCFC2**	POTEA	RSAD2		TMPRSS11A
**ZAP70**	ANKRD20A4	HEATR5B		CDH12
**SCN1A**	PAPPA	EHBP1		DCC
**UNC80**	OR1K1	CTNNA2		
**WDR52**	LBX1	XIRP2		
**XRN1**	HN1L	XIRP2		
**PAQR9**	GSG1L	XIRP2		
**IL1RAP**	NLK	UBR3		
**BRD8**	DOCK6	ITPR1		
**KIAA0319**	ZNFX1	CAND2		
**ABCA13**	PRAF2	ZNF197		
**PLAG1**	DACH2	SEMA3F		
**CYHR1**		TMPRSS7		
**TRPM3**		GABRG1		
**CPXM2**		KIAA1211		
**CABP4**		PTPN13		
**MYO7A**		TSPAN5		
**SCNN1A**		ADAD1		
**GUCY2C**		LRBA		
**KRT76**		ZMAT2		
**ISLR2**		PCDHA7		
**CREBBP**		PCDHA9		
**CDH5**		PCDHA12		
**BCL6B**		JAKMIP2		
**GSDMA**		BAI3		
**KIF18B**		GPRC6A		
**SLC14A1**		SDK1		
**GTF2F1**		KCND2		
**NWD1**		CSPP1		
**MPV17L2**		UBR5		
**PSG3**		PKHD1L1		
**PPP2R1A**		CNTRL		
**VN1R2**		CDH23		
**STAU1**		GRAMD1B		
**LTN1**		VWF		
**SBF1**		CSAD		
**MAP3K15**		ZFC3H1		
**CXorf64**		UBE3B		
**ZNF185**		TEX30		
		TPSB2		
		HS3ST3A1		
		UNK		
		UBE2O		
		USHBP1		
		USHBP1		
		USHBP1		
		ZNF208		
		PPFIA3		
		LILRA1		
		U2AF2		
		ZNF667		

		FASTKD5		
		PTGIS		
		PI4KA		
		ZNRF3		
		APOL5		
		FLG		
		POLR1E		
